# Supplementary material for: Cohort study of the characteristics and outcomes in patients with COVID-19 and in-hospital cardiac arrest
Source: BMJ Open. 2021 Nov 29;11(11):e054943. doi: 10.1136/bmjopen-2021-054943 (PMC8635887; doi:10.1136/bmjopen-2021-054943)
Supplement: Supplementary data [file bmjopen-2021-054943supp001.pdf]

Supplementary figures and tables

Characteristics and Outcomes in Patients with COVID-19 and In-Hospital Cardiac Arrest

Supplementary Table 1: Characteristics of COVID+ patients with IHCA in relation to sex.

Supplementary Table 1: Characteristics of 181 COVID+ patients with IHCA during the COVID-19 pandemic in relation to sex. One COVID+ patient had missing data on sex.

variables	Men	Women	SMD
**n**	113	68	
**Demographics:**			
Age - mean (SD)	71.39 (10.75)	70.35 (14.87)	0.080
**Location of cardiac arrest - n (%):**			0.249
Coronary care unit	7 (6.2)	7 (10.3)	
Intensive care unit	15 (13.3)	10 (14.7)	
Operational room	0 (0.0)	0 (0.0)	
Emergency room	17 (15.0)	11 (16.2)	
Outpatient lab, radiology	4 (3.5)	3 (4.4)	
Cathlab	6 (5.3)	2 (2.9)	
Intermediate care unit	11 (9.7)	4 (5.9)	
Regular ward	52 (46.0)	30 (44.1)	
Other	1 (0.9)	1 (1.5)	
**Critical times - median (IQR):**			
Time to alert – median (IQR)	1.00 [1.00, 1.00]	1.00 [1.00, 1.00]	0.256
Time to CPR - median (IQR)	0.00 [0.00, 0.00]	0.00 [0.00, 1.00]	0.031
Time to defibrillation - median	2.00 [1.00, 5.00]	2.00 [1.00, 2.00]	0.009
**Comorbidities - n (%):**			
MI, ongoing - n (%)	5 (7.6)	7 (21.2)	0.396
MI, previous - n (%)	11 (16.4)	2 (4.7)	0.391
Stroke, ongoing - n (%)	4 (5.8)	0 (0.0)	0.351
Stroke, previous - n (%)	5 (7.0)	2 (4.7)	0.102
Cancer, any - n (%)	13 (18.8)	6 (14.0)	0.132
Diabetes - n (%)	24 (33.3)	12 (27.9)	0.118
Heart failure - n (%)	23 (33.8)	13 (32.5)	0.028
Ejection fraction (%) - mean (SD)	44.84 (12.22)	49.31 (10.56)	0.392
EF <50% - n (%)	19 (51.4)	7 (43.8)	0.153
**Kidney function category - n (%):**			0.357
eGFR <30	16 (22.9)	6 (15.0)	
eGFR 30–59	17 (24.3)	15 (37.5)	
eGFR 60–89	18 (25.7)	7 (17.5)	
eGFR ≥90	19 (27.1)	12 (30.0)	
No kidney failure (eGFR ≥60)	37 (52.9)	19 (47.5)	0.107
eGFR (ml/min/m2) - mean (SD)	72.72 (65.75)	68.70 (45.34)	0.071
**Cause of arrest - n (%):**			0.920
Hemorrhage	1 (1.5)	1 (2.9)	
Myocardial infarction/ischemia	7 (10.6)	8 (23.5)	
Other	18 (27.3)	12 (35.3)	
Primary arrhythmia	3 (4.5)	5 (14.7)	
Respiratory insufficiency	17 (25.8)	7 (20.6)	

Sepsis / infection	18 (27.3)	1 (2.9)	
Stroke / thromboembolism	2 (3.0)	0 (0.0)	
**Early interventions - n (%):**			
Witnessed arrest - n (%)	86 (76.8)	53 (79.1)	0.056
ECG monitoring - n (%)	56 (50.5)	33 (50.0)	0.009
CPR before AGA - n (%)	90 (92.8)	55 (94.8)	0.085
Defibrillated before AGA - n (%)	13 (13.8)	5 (8.9)	0.155
Ventilated before AGA- n (%)	49 (56.3)	25 (53.2)	0.063
Shockable rhythm - n (%)	22 (20.8)	7 (11.5)	0.254
Defibrillated, any - n (%)	29 (26.4)	11 (16.9)	0.231
Intubated - n (%)	61 (57.0)	38 (58.5)	0.029
Adrenaline given - n (%)	76 (70.4)	48 (76.2)	0.132
Antiarrhythmics - n (%)	11 (10.4)	6 (9.7)	0.023
Mechanical compressions - n (%)	12 (10.9)	5 (8.1)	0.097
Active temperature control - n (%)	2 (6.1)	3 (20.0)	0.423
**Status at rescue team arrival - n**			
Consciousness - n (%)	11 (11.3)	6 (10.7)	0.020
Breathing - n (%)	18 (18.6)	11 (19.6)	0.028
Pulse - n (%)	22 (22.7)	13 (23.2)	0.013
**Follow-Up data - n (%):**			
Angiography - n (%)	4 (12.1)	4 (26.7)	0.374
PCI - n (%)	2 (6.1)	2 (13.3)	0.248
Pacemaker implanted - n (%)	0 (0.0)	2 (13.3)	0.555
ICD implanted - n (%)	0 (0.0)	1 (6.7)	0.378
ROSC - n (%)	40 (35.4)	24 (35.3)	0.002
Death at 30 days - n (%)	85 (75.2)	56 (82.4)	0.175
Death overall - n (%)	85 (75.2)	56 (82.4)	0.175
Discharged alive - n (%)	16 (22.2)	6 (14.0)	0.216

SD = standard deviation; IQR = interquartile range; SMD = standardized mean difference (difference between the means for the two groups divided by their mutual standard deviation. Values below 0.1 (10%) are considered inconsequential (i.e., no significant difference between the groups)). CPR = cardiopulmonary resuscitatio*n*, PCI = percutaneous coronary intervention, ICD = implantable cardioverter-defibrillator. ROSC = return of spontaneous circulation. AGA= alarm group arrival.

Supplementary Figure 1: Missing data before and after imputation with MICE

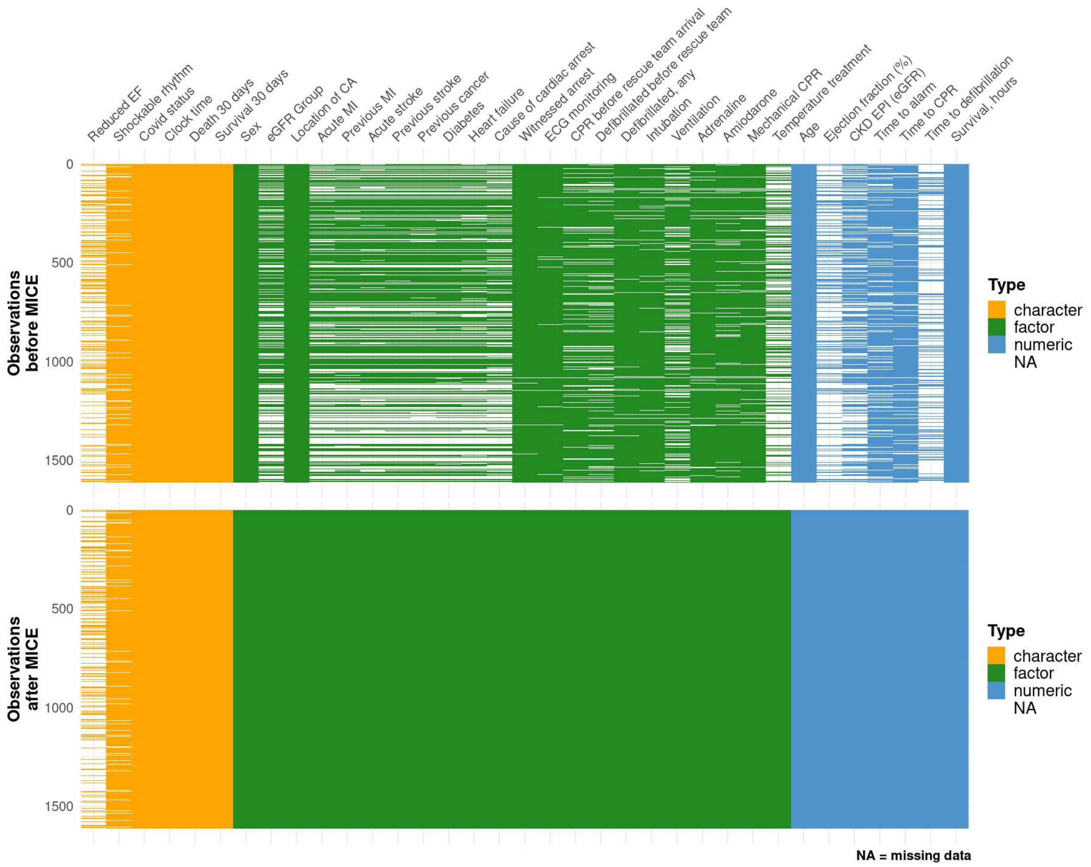

Supplementary Figure 1: Missing data before and after imputation with MICE. A graphical view of the entire dataset is printed. Each column (variable) is depicted at the top and column color depicts type of variable. Each patient represents a row and white gaps indicate a missing data entry.

Supplementary Figure 2: Flow chart

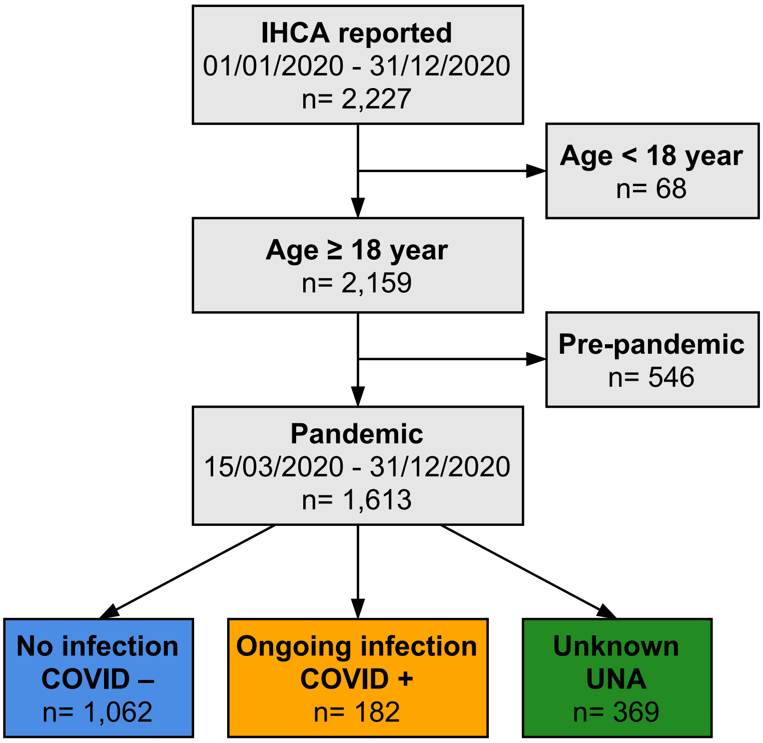

Supplementary Figure 2: Flow chart of the study population. Patients who were less than 18 year of age, and cases occurring in the pre-pandemic period were excluded.

Supplementary Figure 3: Information on COVID-19 status during the study period.

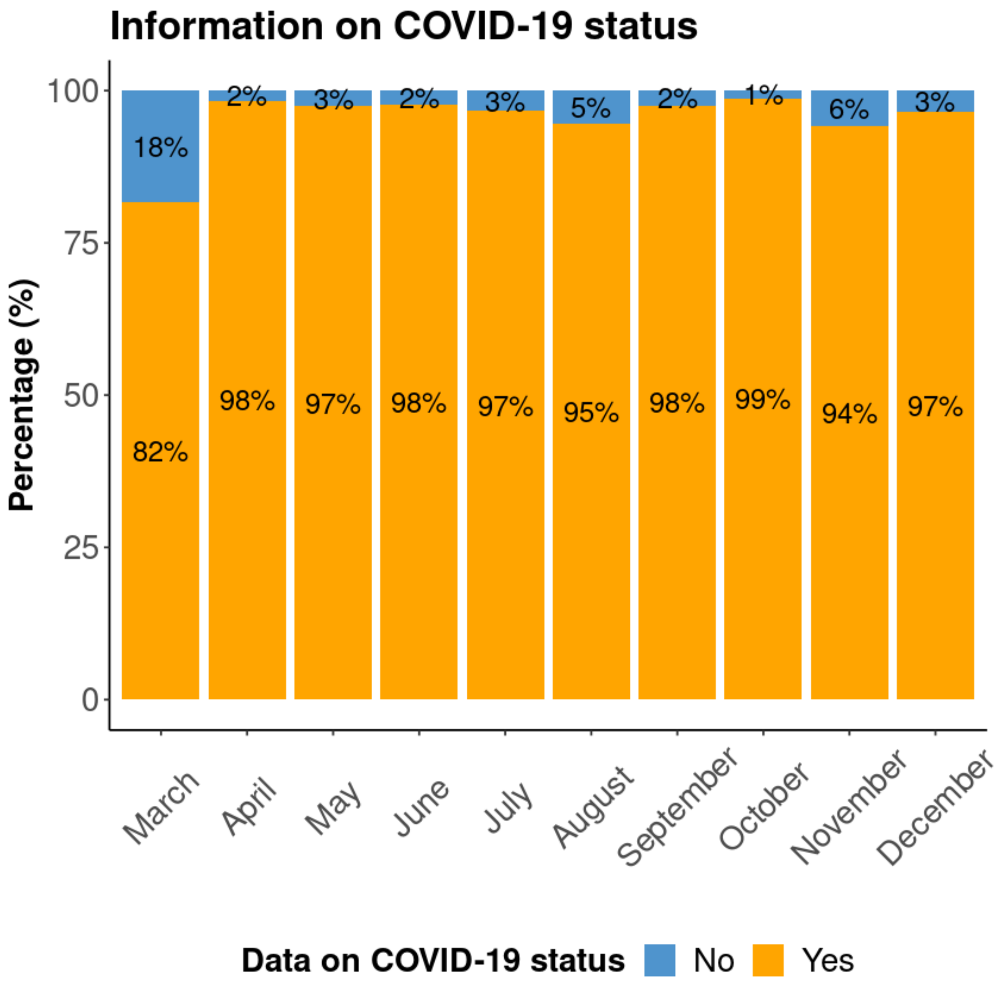

Supplementary Figure 3: Information on COVID-19 status during the study period. No equals missing data, i.e. no information on COVID-19 status available. Yes equals, COVID +, COVID – or Unknown. In March only cases after 15/03/2020 were included.

Supplementary Figure 4: Distribution of age

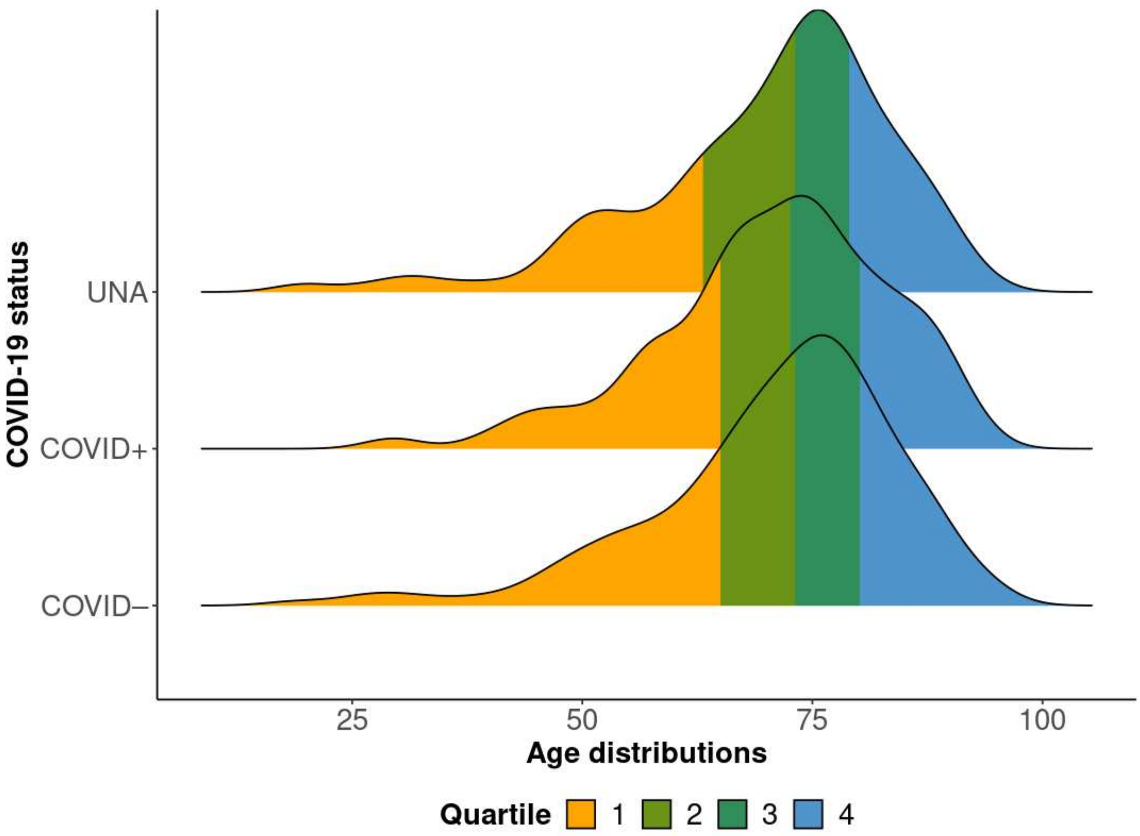

Supplementary Figure 4: Distribution of age in relation to COVID-19 status.

Supplementary Figure 5: Etiology of IHCA, according to sex

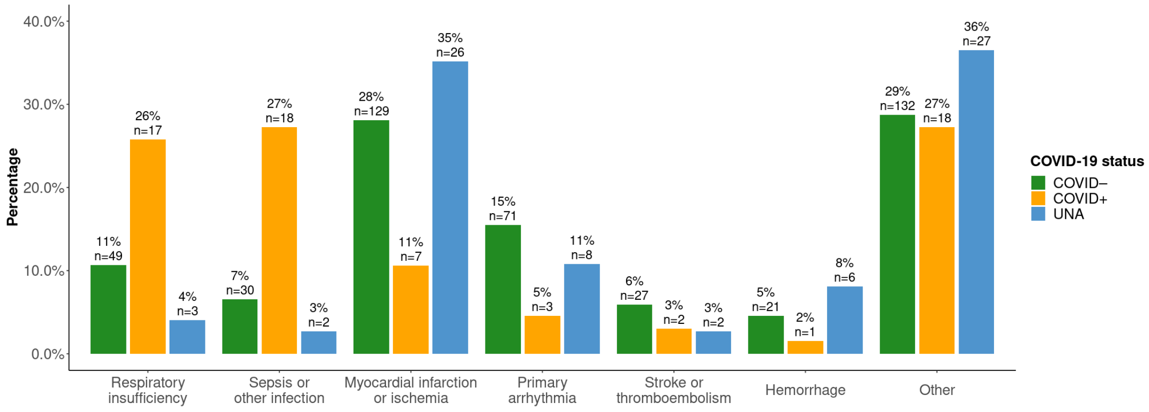

Supplementary Figure 5A: Etiology of IHCA, men only.

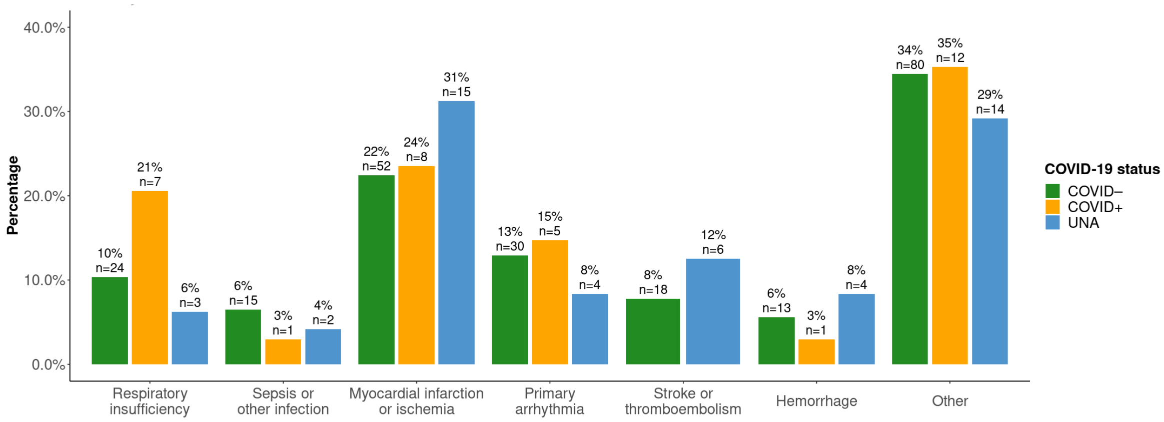

Supplementary Figure 5B: Etiology of IHCA, women only.

Supplementary Figure 6: Conditions preceding IHCA, according to sex

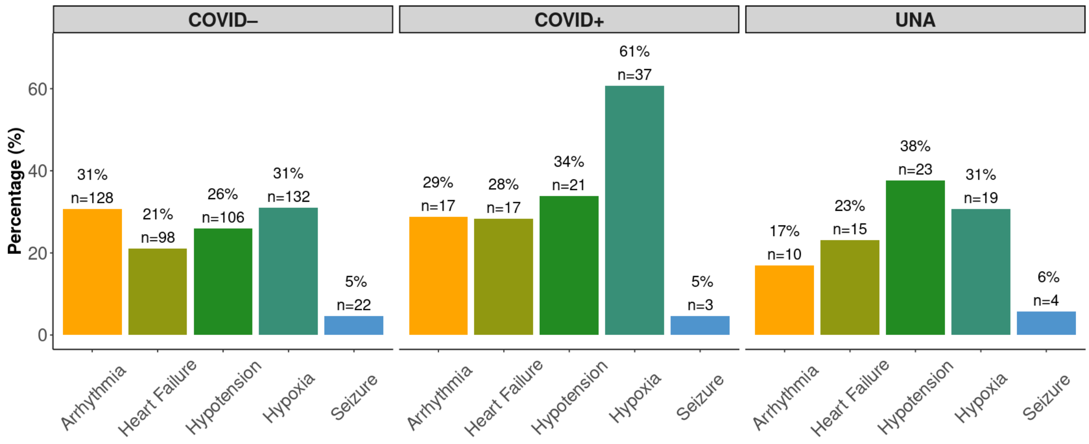

Supplementary Figure 6A: Conditions preceding IHCA, men only.

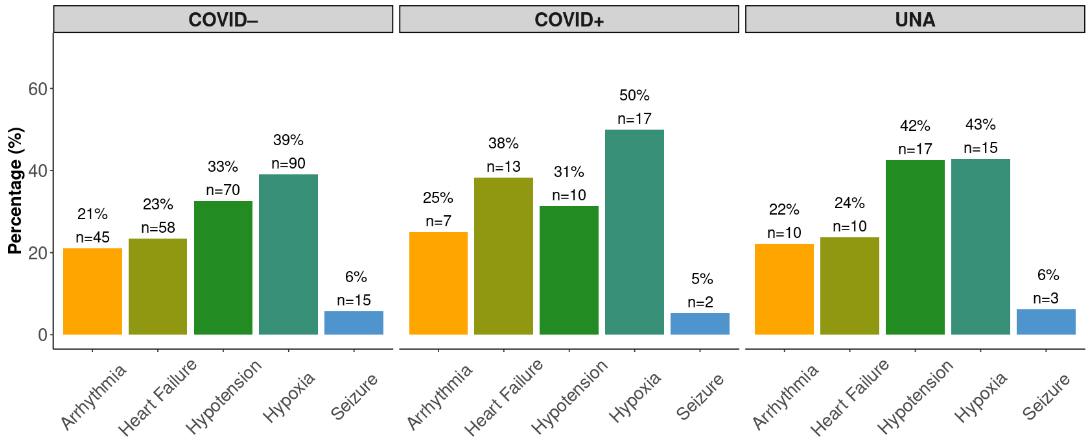

Supplementary Figure 6B: Conditions preceding IHCA, women only.

Supplementary Figure 7: Cox adjusted survival curve for the overall population

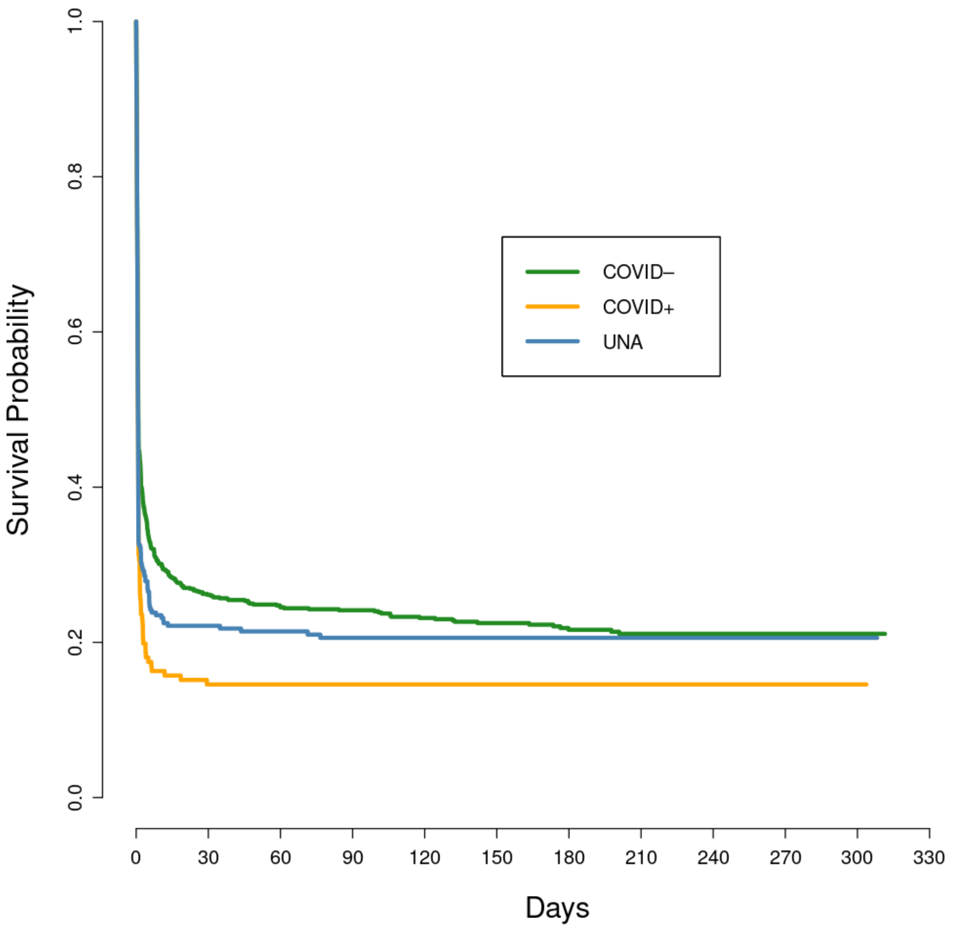

Supplementary Figure 7: Cox adjusted survival curve for the overall population, stratified on COVID-19 status.
