# Supplementary material for: Cohort study of the characteristics and outcomes in patients with COVID-19 and in-hospital cardiac arrest
Source: BMJ Open. 2021 Nov 29;11(11):e054943. doi: 10.1136/bmjopen-2021-054943 (PMC8635887; doi:10.1136/bmjopen-2021-054943)
Supplement: Author's manuscript [file bmjopen-2021-054943.draft_revisions.pdf]

BMJ Open

BMJ Open is committed to open peer review. As part of this commitment we make the peer review history of every article we publish publicly available.

When an article is published we post the peer reviewers' comments and the authors' responses online. We also post the versions of the paper that were used during peer review. These are the versions that the peer review comments apply to.

The versions of the paper that follow are the versions that were submitted during the peer review process. They are not the versions of record or the final published versions. They should not be cited or distributed as the published version of this manuscript.

BMJ Open is an open access journal and the full, final, typeset and author-corrected version of record of the manuscript is available on our site with no access controls, subscription charges or pay-per-view fees (http://bmjopen.bmj.com).

If you have any questions on BMJ Open's open peer review process please email info.bmjopen@bmj.com

BMJ Open

A Cohort Study of the Characteristics and Outcomes in Patients with COVID-19 and In-Hospital Cardiac Arrest

Journal:	*BMJ Open*
Manuscript ID	bmjopen-2021-054943
Article Type:	Original research
Date Submitted by the Author:	06-Jul-2021
Complete List of Authors:	Holm, Astrid; University of Gothenburg, Department of Molecular and Clinical Medicine<br>Jerkeman, Matilda; University of Gothenburg, Department of Molecular and Clinical Medicine<br>Sultanian, Pedram; University of Gothenburg, Department of Molecular and Clinical Medicine<br>Lundgren, Peter; University of Gothenburg, Department of Molecular and Clinical Medicine; Kalmar County Hospital, Department of Internal Medicine<br>Ravn-Fischer, Annica; University of Gothenburg, Department of Molecular and Clinical Medicine<br>Israelsson, Johan; Kalmar County Hospital, Department of Internal Medicine; Linnaeus University, Faculty of Health and Life Sciences<br>Giesecke, Jasna; Danderyd University Hospital<br>Herlitz, Johan; University of Borås, Centre for Prehospital Research<br>Rawshani, Araz; University of Gothenburg, Department of Molecular and Clinical Medicine
Keywords:	COVID-19, CARDIOLOGY, Adult cardiology < CARDIOLOGY, Coronary heart disease < CARDIOLOGY

SCHOLARONE™
Manuscripts

I, the Submitting Author has the right to grant and does grant on behalf of all authors of the Work (as defined in the below author licence), an exclusive licence and/or a non-exclusive licence for contributions from authors who are: i) UK Crown employees; ii) where BMJ has agreed a CC-BY licence shall apply, and/or iii) in accordance with the terms applicable for US Federal Government officers or employees acting as part of their official duties; on a worldwide, perpetual, irrevocable, royalty-free basis to BMJ Publishing Group Ltd ("BMJ") its licensees and where the relevant Journal is co-owned by BMJ to the co-owners of the Journal, to publish the Work in this journal and any other BMJ products and to exploit all rights, as set out in our licence.

The Submitting Author accepts and understands that any supply made under these terms is made by BMJ to the Submitting Author unless you are acting as an employee on behalf of your employer or a postgraduate student of an affiliated institution which is paying any applicable article publishing charge ("APC") for Open Access articles. Where the Submitting Author wishes to make the Work available on an Open Access basis (and intends to pay the relevant APC), the terms of reuse of such Open Access shall be governed by a Creative Commons licence – details of these licences and which Creative Commons licence will apply to this Work are set out in our licence referred to above.

Other than as permitted in any relevant BMJ Author's Self Archiving Policies, I confirm this Work has not been accepted for publication elsewhere, is not being considered for publication elsewhere and does not duplicate material already published. I confirm all authors consent to publication of this Work and authorise the granting of this licence.

For peer review only

A Cohort Study of the Characteristics and Outcomes in Patients with COVID-19 and In-Hospital Cardiac Arrest

Astrid Holm, MD[1], Matilda Jerkeman, MD[1], Pedram Sultanian, MD[1], Peter Lundgren, MD, PhD[1,3,7], Annica Ravn-Fischer, MD, PhD, Docent[1], Johan Israelsson, PhD[3,4], Jasna Giesecke, RN[5], Johan Herlitz, MD, PhD[2,6], Araz Rawshani, MD, PhD[1,2]

Affiliations:

[1]Department of Molecular and Clinical Medicine, Institute of Medicine, Sahlgrenska Academy, University of Gothenburg, Sweden

[2]The Swedish Registry for Cardiopulmonary Resuscitation, Centre of Registries, Västra Götaland County, Sweden

[3]Department of Internal Medicine, Division of Cardiology, Kalmar County Hospital, Region Kalmar County, Sweden

[4]Faculty of Health and Life Sciences, Linnaeus University, Kalmar, Sweden

[5]RN, Lead CPR coordinator, Clinicum- Centre for clinical skills, interprofessional education and advanced medical simulation, Danderyd University Hospital, Stockholm, Sweden

[6]Prehospen – Centre for Prehospital Research, University of Borås, Borås, Sweden

[7]Region Västra Götaland, Sahlgrenska University Hospital, Department of Cardiology, Gothenburg, Sweden

Contact information:

Astrid Holm
Email: astrid.holm@gu.se

Phone: +358 400707722

Manuscript word count: 3592

Keywords: COVID19, SARS-COV-2, Coronavirus, cardiac arrest, in-hospital cardiac arrest

Key Points

Question: What are the Characteristics, causes and outcomes in patients with COVID-19 who suffer an in-hospital cardiac arrest (IHCA)?

Findings: In the registry-based observational study we found that during pandemic peaks, up to one fourth of all IHCAs are complicated by COVID-19, and these patients have halved chance of survival.

Meaning: The survival rate of patients with COVID-19 associated IHCA is low with women displaying the worst outcomes.

Abstract

Objective: We studied characteristics, survival, causes of cardiac arrest, conditions preceding cardiac arrest, predictors of survival, and trends in the prevalence of COVID-19 among IHCA cases. Data on characteristics and outcomes in patients with COVID-19 who suffer an in-hospital cardiac arrest (IHCA) is scarce.

Design and setting: Registry-based observational study.

Participants: We studied all cases of IHCA receiving CPR (≥18 years of age) in the Swedish Registry for Cardiopulmonary Resuscitation from 15/03/2020 to 31/12/2020. A total of 1613

patients were included and divided into the following groups: ongoing infection (**COVID+;** n=182), no infection (**COVID–;** n=1062) and unknown/not assessed (**UNA**; n=369).

Main outcomes and measures: We studied monthly trends in proportions of COVID-19 associated IHCAs, causes of IHCA in relation to COVID-19 status, clinical conditions preceding the cardiac arrest and predictors of survival.

Results: The rate of COVID+ patients suffering an IHCA increased to 23% during the first pandemic wave (April), then abated to 3% in July, and then increased to 19% during the second wave (December). Among COVID+ cases, 43% had respiratory insufficiency or infection as the underlying cause of the cardiac arrest, compared to 18% among COVID– cases. The most common clinical sign preceding cardiac arrest was hypoxia (57%) among COVID+ cases. Odds ratio for 30-day survival for COVID+ cases was 0.50 (95% CI 0.33-0.76) compared with COVID– cases. At the end of follow-up, 19% of COVID+ cases and 35.6% of COVID– cases had been discharged alive. Among COVID+ cases, 22% of men, compared with 14% of women, were discharged alive.

Conclusion: During pandemic peaks, up to one fourth of all IHCAs are complicated by COVID-19, and these patients have halved chance of survival, with women displaying the worst outcomes.

Article Summary

Strengths and limitations of this study

- A major strength of our study is that it includes all IHCAs in Sweden which were reported to the Swedish Registry for Cardiopulmonary Resuscitation.
- The sample recorded in the Swedish Registry for Cardiopulmonary Resuscitation is unbiased since all hospitals participate in the registry and all hospitals report data on COVID-19 status
- A limitation is that we do not know the severity of the COVID-19 infection, and we do not know if COVID-19 was the main reason for admission to hospital.
- Our study only includes IHCAs receiving CPR which leaves out all other patients with IHCA, e.g with a Do Not Attempt Resuscitation order.
- It is important to stress the fact that our regression model that included only COVID-19 cases must be interpreted with caution due to the large number of predictors in the model, which had relatively few patients.

Introduction

The COVID-19 pandemic has, as of May 1st 2021 infected over 159,000,000 persons and lead to the demise of over 3,321,000 individuals(1).The Swedish Public Health Authority declared on March 16th 2020 that community spread of COVID-19 had commenced, and COVID-19 is now the third leading cause of death in Sweden(2, 3).

A recent study including over 5,000 critically ill patients with COVID-19 showed that in-hospital cardiac arrest (IHCA) is common and associated with poor survival(4). An early study from Wuhan also showed poor survival after IHCA among COVID-19 patients(5). We recently studied IHCA in the Swedish Registry for Cardiopulmonary Resuscitation (SRCR) and showed a 2.3-fold increase in 30-day mortality among cases with COVID-19 compared to pre-pandemic cases and this was mainly driven by a 9-fold increase in mortality among women with COVID-19. During the study period no case of IHCA with COVID-19 was discharged alive from the hospital(6).

The current study expands our previous investigation, including more patients, longer follow-up and emphasizes the causes of cardiac arrest in COVID-19, predictors of survival, coexisting conditions, and trends in the prevalence of COVID-19 among IHCA cases.

Methods

Data sources

The study is a registry-based observational study with data obtained from the SRCR during the time period 15/03/2020 to 31/12/2020.

The SRCR is a national quality registry and has included IHCA cases since 2005. The data is collected by trained nurses who report patient data using a web-based protocol. The registry has previously been described in detail(7). Vital status was obtained from the Swedish Population Registry and the last day of follow up was 31/12/2020.

Study population

The study population included all patients ≥18 years of age suffering from IHCA and receiving CPR throughout Sweden during the period 15/03/2020 to 31/12/2020. We used 15th of March as the start date of the pandemic as the Swedish Public Health Authority declared on March 16th 2020 that COVID-19 was community spread in Sweden(3). On 1st of April the SRCR started collecting data about COVID-19 status, and retrospectively identified 60 patients with COVID-19 who suffered IHCA during March (they were included in the study). Patients were divided into the following three groups: ongoing infection (COVID+; n=182), no infection (COVID–; n=1062) and unknown/not assessed (UNA; n=369). COVID+ was

defined as patients registered with an ongoing COVID-19 infection, suspected ongoing infection or patients with a recent infection(n=29).

Variable definitions

In SRCR a patient with cardiac arrest was defined as an unconscious patient with no or abnormal breathing, in whom resuscitation or defibrillation was attempted. IHCA was defined as cardiac arrest in patients admitted to the hospital.

With regards to previous coexisting conditions heart failure was defined as any heart failure described before cardiac arrest. Kidney failure was defined as estimated glomerular filtration rate (eGFR) below 60 ml/min/1.73 m^2, calculated using the highest creatinine before cardiac arrest with Chronic Kidney Disease Epidemiology Collaboration (CKD-EPI) formula based on sex, age and creatinine. The SRCR records data on the highest creatinine levels analyzed up to six months prior to CA. Diabetes was defined as any diabetes diagnosis, regardless of type. Cancer was defined as any previously known cancer. Acute myocardial infarction (MI) was defined as an MI within 72 hours of CA. Previous myocardial infarction was defined as MI occurring earlier than 72 hours preceding the CA.

Regarding clinical conditions one hour prior to CA, arrhythmia was defined as any arrhythmia, hypoxia was defined as an oxygen saturation below 90%, hypotension was

defined as systolic blood pressure below 90 mmHg, seizure was defined as any seizure with loss of consciousness, and heart failure was defined as any heart failure with pulmonary edema or severe shortness of breath with rales.

A monitored ward was defined as a coronary care unit(CCU), an intensive care unit(ICU), an operational room(OR), an emergency room(ER), an intermediate care unit(IMCU) or a catheterization laboratory(Cath lab). A non-monitored ward was defined as a regular ward (RW). All other wards were defined as other ward, e.g. outpatient lab, radiology department, etc.

Statistical analyses

Patient characteristics are reported in means and medians, along with standard deviations and interquartile ranges, respectively. The Kaplan-Meier estimator was used for defining survival distributions; the log rank test was used to test for differences in survival.

Logistic regression was used to calculate odds ratios for 30-days survival. These models assessed the association between COVID-19 status and 30-days survival, while adjusting for age, sex and initial rhythm (shockable or non-shockable). Subgroup analyzes were done for men, women, age ≥70 years, age <70 years, heart failure, kidney failure, diabetes, myocardial infarction and cancer.

In order to obtain estimates of overall survival, we used Cox proportional hazards model with hours since CA as the time scale. The proportional hazards assumption was fulfilled for all variables.

We used the MICE (Multiple Imputation By Chained Equations) algorithm to impute missing values(8, 9) (Supplementary Figure 1). The imputed data set was used to calculate odds ratios for 30-days survival in the overall group, as well as in COVID+ and COVID– cases. These models included age, sex, initial rhythm, time to start of cardiopulmonary resuscitation (CPR), time of CA, previous MI, location (other ward vs monitored, and non-monitored ward vs monitored), heart failure, EKG monitoring, diabetes and acute MI.

Analyses were done in R (v. 4.0.3, R Foundation for Statistical Computing) using RStudio.

The study was approved by the Swedish Ethical Review Authority (ID 2020-02017).

Patient and Public Involvement statement:

No patients were involved.

Results

A total of 2,227 patients were enrolled in the SRCR between 01/01/2020 and 31/12/2020. After excluding patients <18 years (n=68) and pre-pandemic cases (n=546), 1,613 cases remained from 15/03/2020 to 31/12/2020 and constituted the final study population

(Supplementary Figure 2). There was a high rate of information on COVID-19 status during the study period among patients registered in the registry (Supplementary Figure 3).

Baseline characteristics

The overall mean age was 70.8 years, and the proportion of women was 37.6%. At the end of follow-up, 341 (32.7%) patients were alive. The mean age was similar in the three groups: 70.9 years among COVID+, 71.0 years among COVID– cases, and 70.2 years in cases with UNA (Supplementary Figure 4). The proportion of women was also similar; 37.6% in COVID+, 36.6% in COVID– and 41.0% in UNA cases.

A regular ward (RW) was the most common place of cardiac arrest in all 3 groups with rates of 45.1% among COVID+, 44.1% among COVID– and 31.4% among UNA (Table 1). The emergency room (ER) was the second most common location for COVID+ cases (15.9%). The ER was the location of cardiac arrest in 17.6% of UNA cases and 13.1% for COVID– cases.

Regarding comorbidities, acute myocardial infarction was observed in 12.0% of COVID+ and 23.6% of COVID– cases. Previous myocardial infarction was observed in 11.7% of COVID+, 20.8% of COVID– and 11.7% of UNA cases. The prevalence of heart failure, cancer and diabetes was similar across all groups (Table 1).

Fewer cases among COVID+ individuals had a shockable rhythm (17.3%), compared with COVID– (24.9%) and UNA (27.0%). Likewise, fewer cases among COVID+ (22.7%) were defibrillated, compared with COVID– (31.5%) and UNA (32.8%). COVID+ cases were ventilated in 54.8% of cases before rescue team arrival, as compared with 63.2% and 69.2% in COVID– and UNA, respectively.

Follow-up and crude survival

Return of spontaneous circulation (ROSC) after initial resuscitation, was less common in COVID+ cases, as compared with COVID– and UNA. Also, angiography, PCI, pacemaker and ICD implantation post cardiac arrest were less common in COVID+ cases.

Survival at 30-days was 37.3% in COVID– patients, compared to 22.5% among COVID+ patients (Table 1), and 35.6% of COVID– patients were discharged alive, compared with 19.0% of COVID+ patients.

Sex specific characteristics

Acute myocardial infarction was observed in 21.2% of COVID+ women and 7.6% of COVID+ men. Previous myocardial infarction was observed in 4.7% of COVID+ women and 16.2% of COVID+ men. The prevalence of previous stroke, renal failure, heart failure, cancer

and diabetes were similar among men and women, as was location at the time of cardiac arrest. COVID+ men were more likely to have a shockable rhythm (20.8%) compared with COVID+ women (11.5%) and to be defibrillated (26.4% in men vs 16.9% in women). In all, 22.2% of COVID+ men were discharged alive from hospital compared to 14.0% of COVID+ women (Supplementary Table 1).

Monthly trends in COVID-19 associated IHCA

In March, April and May 14%, 23% and 20% of patients suffering IHCA were COVID+ (data from 16th March). The proportion of COVID+ cases diminished rapidly during June to July. From September onwards the COVID+ cases increased again to reach 19% in December. In Figure 1A additional details regarding monthly variations are presented.

Etiology of IHCA

The most common cause of IHCA among COVID+ was respiratory insufficiency (24%, n=24). The second most common cause was sepsis or other infection (19%, n=19) among COVID+. Respiratory insufficiency and sepsis/other infection were less common in the other groups (Figure 1B), which instead displayed higher rates of acute myocardial infarction.

Clinical conditions one hour prior to IHCA

As evident in Figure 1C which describes the clinical conditions preceding (up to 60 minutes) the cardiac arrest, hypoxia was more common among COVID+ (57%), as compared with COVID– (34%). Regarding arrhythmia, heart failure, hypotension and seizure the percentages were more similar.

Survival analysis

The Kaplan Meier plots (Figure 2) show that COVID+ cases generally had a lower probability of survival compared to COVID– and UNA cases. The overall 30-day survival (Figure 2A) was 21% among COVID+, compared with 36% in COVID– cases (p=0.00086). The subgroup analysis of women (Figure 2B) showed low survival rates in COVID+ cases (16% 30-day survival). Regarding age, 30 days survival among COVID+ aged <70 years was 25% (Figure 2E), as compared with 18% of COVID+ cases aged 70 or older (Figure 2D). Patients with acute MI had a 30 days survival of 8% among COVID+ cases (Figure 2J).

Survival curves for the subgroups of individuals with cancer, heart failure and diabetes, did not display any clear patterns (Figure 2F-2H). All p values were >0.1.

Cox adjusted survival curves are presented in Supplementary Figure 5; COVID+ cases displayed the lowest probability of survival, whereas there was no material difference between COVID– and UNA cases.

Odds ratios for 30-days survival

When adjusted for age, sex and initial rhythm the odds ratio for 30-day survival, comparing COVID+ vs. COVID–, were 0.50 (0.33-0.76) overall, 0.53 (0.31-0.88) for men, and 0.44 (0.20-0.88) for women. In the subgroup of patients with heart failure, myocardial infarction and cancer, we found no statistically significant associations, whereas in the subgroup of COVID+ patients with kidney failure, odds ratio for 30-days survival was 0.43 (0.16–0.99), when compared with COVID– (Figure 3).

Predictors of survival

Regarding predictors for 30-days survival among COVID+ we note that confidence intervals were generally wide. Lack of ECG monitoring and later start of CPR showed point estimates below 1.0, although non-significant. Odds ratio for patients treated in non-monitored wards

was 0.26 (95% CI 0.08-0.78) as compared with monitored ward(Figure 4). No coexisting condition was associated with survival among COVID+ cases.

Regarding COVID– cases the factors that were significantly associated with 30-days survival were shockable rhythm (OR 4.18 [95% CI 2.69–6.02]), ECG monitoring (2.67 [95% CI 1.82–3.95]), heart failure (OR 0.58 [95% CI 0.40–0.83]) and diabetes (OR 0.64 [95% CI 0.44–0.92]) were significantly associated with death(Figure 4).

Discussion

This study elucidates characteristics and outcomes in patients with COVID-19 who develop IHCA. To the best of our knowledge, this is the largest study on IHCA with individual level COVID-19 data. We show that most characteristics (e.g., underlying etiology, initial rhythm, conditions preceding cardiac arrest), as well as survival, differs markedly in COVID+ cases compared with COVID–, with the former group exhibiting worse characteristics and outcomes. Importantly, survival in COVID+ cases was half that of COVID– cases. As of writing this report the pandemic is still surging worldwide with hundreds of thousands of new cases every day. The results of our study are relevant for any health care system handling patients infected with COVID-19.

Regarding location of CA, we note that the most common location for COVID-19 patients was regular wards, which are not monitored. This is unfortunate since our regression analysis showed that type of ward (monitored vs non-monitored) was significantly associated with survival, such that COVID+ cases in non-monitored wards displayed 74% lower probability of survival as compared with COVID+ cases in monitored wards. As compared with COVID– cases, cardiac arrest in the ER was more common in COVID+ cases. The often rapid deterioration of cardiopulmonary function in patients with COVID-19 may be one of the explanations for this finding. Fewer COVID+ cases were located in the CCU which is an expected finding due to the fact that cardiac etiology was less common among these patients.

There were high rates of COVID-19 associated cases in April and May and as expected the number of patients started increasing again from September onwards to reach 19% in December at the end of our study. At the moment the incidence of severe COVID-19 cases in Sweden is still high.

In this study we note that the most common cause of cardiac arrest in COVID+ cases, as well as the most frequent clinical condition directly preceding the arrest, is respiratory. The high rate of respiratory etiology was driven by men (Supplementary Figure 6-7). A total of 57% of cases displayed hypoxia before cardiac arrest. This may highlight an opportunity for

improving outcomes; measures to prevent hypoxia and to correct it immediately may reduce the risk of cardiac arrest in patients with COVID-19. On the other hand, it can be argued that we cannot do that inference because we have not studied patients with and without hypoxia and followed them in terms of risk of developing cardiac arrest (all our cases had already developed cardiac arrest). However, we know that COVID-19 causes ARDS (acute respiratory distress syndrome) and hypoxia, which can induce cardiac arrest.

The fact that COVID+ cases were ventilated (prior to arrival of the rescue team) less frequently than all other patients was expected, despite the fact that they displayed higher rates of hypoxia prior to cardiac arrest, as well as respiratory etiology. This observation is likely explained by the fact that Swedish guidelines were revised during March 2020 as well as the guidelines from the European Resuscitation Council, with the recommendation that mouth-to-mouth ventilation and pocket mask ventilation should be avoided in case with confirmed or suspected COVID-19(10). Whether or not this resulted in worse outcomes for COVID- 19 cases remain unsolved.

However, the fact that 43% of cases with COVID-19 did not have hypoxia prior to cardiac arrest suggests that other factors are important as well. Thromboembolism, myocardial infarction, arrhythmias, etc. may all contribute to the development of a cardiac arrest(11).

A previous study from Wuhan showed that 87.5% of COVID+ cases with in-hospital cardiac arrest had a respiratory etiology(5). We report much lower rates (24%), which may be due to several factors; e.g. in our study we had a total of 22 possible categories for cause of CA, as compared with two categories in the study from Wuhan. Also, patients in the study from Wuhan had severe COVID-19 and in our study population we do not know the severity of the disease.

The survival rates were poor among COVID+ patients with an overall 30-days survival of 21%, compared to 36% among COVID–. The survival rate was, however, not as low as in the study from Wuhan, in which 3% (151 patients studied) survived, or in the study from New York with 31 patients with none surviving(5, 12). One reason for the poor survival could be the small number of patients found in a shockable rhythm (17% vs. 25% for COVID+ and COVID–, respectively) since patients with shockable rhythm have a more favorable outcome. After adjusting for sex, age and shockable rhythm the 30-day survival was though still significantly worse among patients with an ongoing infection.

In our previous study we showed that COVID+ women had an odds ratio of 7.63 (1.97−50.93) for 30-days mortality, as compared with COVID– women. The wide confidence interval in the previous study is mostly explained by lack of statistical power. In this study, in

which more patients were included, we demonstrate that COVID+ women had halved chance of survival at 30 days, compared with COVID– women(6). We find it interesting that COVID+ women had acute MI three times as often as men, despite the fact that men exhibited shockable rhythm – and were defibrillated – twice as often as women; this cannot be explained by differences in prevalent heart failure, as there were none across men and women.

Strengths and limitations. This study includes all IHCAs in Sweden which were reported to SRCR. The sample recorded in the SRCR is unbiased since all hospitals participate in the registry and all hospitals report data on COVID-19 status. However, we do not know the severity of the COVID-19 infection, and we do not know if COVID-19 was the main reason for admission to hospital. With regards to the classification of COVID-19 status, we have recently performed a misclassification analysis which demonstrated that odds ratios were not materially affected by misclassification bias. Our study only includes IHCAs receiving CPR. This leaves out all other patients with IHCA, e.g with a Do Not Attempt Resuscitation order.

It is important to stress the fact that our regression model that included only COVID-19 cases must be interpreted with caution due to the large number of predictors in the model, which had relatively few patients (resulting in wide confidence intervals). Further studies are

warranted, using a larger study population, and a longer follow up especially regarding subgroup analyses, neurological outcomes and the quality of life for the patients. Information about the severity of COVID-19 and the reason for admission to the hospital would add valuable insights as well.

Conclusion

During pandemic peaks, up to one fourth of all IHCAs are complicated by COVID-19, and these patients have halved chance of survival, with women displaying the worst outcomes. While our previous study did not identify any COVID+ cases that were discharged alive, we now show that 19% of COVID+ cases are discharged alive, which is half the rate among COVID– cases.

Funding

This work was supported by the Swedish Research Council [2019-02019] and the Swedish Heart and Lung Foundation [20200261].

Conflict of interest: none declared.

Author Statement: Astrid Holm and Araz Rawshani designed the study. Astrid Holm has been the main author and been in charge of the analysis and interpretation of data. Araz Rawshani

has been supervising. Matilda Jerkeman, Pedram Sultanian, Peter Lundgren, Annica Ravn-Fischer, Johan Israelsson, Jasna Giesecke and Johan Herlitzwere were all co-writers, revised the article critically for important intellectual content and approved the version of the article to be published.

References

1. Johns Hopkins University and Medicine COVID-19 map [internet] Johns Hopkins Coronavirus Resource Centre 2020. [updated 13/5/2021. Available from: https://coronavirus.jhu.edu/map.html.
2. Dödsorsaker första halvåret 2020. Socialstyrelsen. Hälso- och sjukvård.; 2020 17/11/2020.
3. Andersson J. Samhällsspridning av coronaviruset i Sverige. Läkartidningen. 16/03/2020.
4. Hayek SS, Brenner SK, Azam TU, Shadid HR, Anderson E, Berlin H, et al. In-hospital cardiac arrest in critically ill patients with covid-19: multicenter cohort study. Bmj. 2020;371:m3513.
5. Shao F, Xu S, Ma X, Xu Z, Lyu J, Ng M, et al. In-hospital cardiac arrest outcomes among patients with COVID-19 pneumonia in Wuhan, China. Resuscitation. 2020;151:18-23.
6. Sultanian P, Lundgren P, Strömsöe A, Aune S, Bergström G, Hagberg E, et al. Cardiac arrest in COVID-19: characteristics and outcomes of in- and out-of-hospital cardiac arrest. A report from the Swedish Registry for Cardiopulmonary Resuscitation. European Heart Journal. 2021.
7. Hessulf F, Herlitz J, Rawshani A, Aune S, Israelsson J, Södersved-Källestedt ML, et al. Adherence to guidelines is associated with improved survival following in-hospital cardiac arrest. Resuscitation. 2020;155:13-21.
8. Stef van Buuren, Groothuis-Oudshoorn K. mice: Multivariate Imputation by Chained Equations in R. Journal of Statistical Software. 2011;Vol 45.
9. 3.5 Classification and regression trees [Available from: https://stefvanbuuren.name/fimd/sec-cart.html.
10. Nolan JP, Monsieurs KG, Bossaert L, Böttiger BW, Greif R, Lott C, et al. European Resuscitation Council COVID-19 guidelines executive summary. Resuscitation. 2020;153:45-55.
11. Gupta A, Madhavan MV, Sehgal K, Nair N, Mahajan S, Sehrawat TS, et al. Extrapulmonary manifestations of COVID-19. Nat Med. 2020;26(7):1017-32.
12. Sheth V, Chishti I, Rothman A, Redlener M, Liang J, Pan D, et al. Outcomes of in-hospital cardiac arrest in patients with COVID-19 in New York City. Resuscitation. 2020;155:3-5.

Table 1 Characteristics of 1613 patients with IHCA during the COVID-19 pandemic.

**Variables**	**No infection COVID –**	**Ongoing infection COVID +**	**Unknown/NA UNA**	**SMD**
**n**	1062	182	369	
**Demographics:**				
Age - mean (SD)	71.00 (13.32)	70.93 (12.43)	70.22 (13.60)	0.039
Woman - n (%)	388 (36.6)	68 (37.6)	151 (41.0)	0.061
**Location of cardiac arrest - n (%)**				0.527
Coronary care unit - n (%)	155 (14.6)	14 (7.7)	50 (13.6)	
Intensive care unit - n (%)	77 (7.3)	25 (13.7)	19 (5.1)	
Operational room - n (%)	22 (2.1)	0 (0.0)	12 (3.3)	
Emergency room - n (%)	139 (13.1)	29 (15.9)	65 (17.6)	
Outpatient lab, radiology - n (%)	49 (4.6)	7 (3.8)	28 (7.6)	
Cathlab - n (%)	98 (9.2)	8 (4.4)	60 (16.3)	
Intermediate care unit - n (%)	25 (2.4)	15 (8.2)	10 (2.7)	
Regular ward - n (%)	468 (44.1)	82 (45.1)	116 (31.4)	
Other - n (%)	29 (2.7)	2 (1.1)	9 (2.4)	
**Critical times - median (IQR):**				
Time to alert – median (IQR)	1.00 [1.00, 1.00]	1.00 [1.00, 1.00]	1.00 [1.00,	0.078
Time to CPR - median (IQR)	0.00 [0.00, 1.00]	0.00 [0.00, 0.00]	0.00 [0.00,	0.109
Time to defibrillation - median (IQR)	2.00 [1.00, 5.00]	2.00 [1.00, 4.75]	1.00 [1.00,	0.141
**Comorbidities - n (%):**				
MI, ongoing - n (%)	178 (23.6)	12 (12.0)	37 (29.4)	0.292
MI, previous - n (%)	163 (20.8)	13 (11.7)	26 (18.4)	0.165
Stroke, ongoing - n (%)	30 (3.8)	4 (3.7)	4 (3.0)	0.030
Stroke, previous - n (%)	82 (10.3)	7 (6.1)	15 (10.5)	0.105
Cancer, any - n (%)	165 (20.9)	20 (17.7)	28 (20.6)	0.054
Diabetes - n (%)	224 (27.9)	36 (31.0)	38 (27.0)	0.060
Heart failure - n (%)	229 (29.7)	36 (33.0)	36 (27.9)	0.074
Ejection fraction (%) - mean (SD)	46.14 (13.74)	46.44 (11.86)	44.94 (14.82)	0.073
EF <50% - n (%)	167 (46.0)	26 (48.1)	22 (46.8)	0.029
**Kidney function category - n (%)**				0.121
eGFR <30 - n (%)	165 (21.6)	22 (20.0)	26 (20.0)	
eGFR 30–59- n (%)	216 (28.3)	32 (29.1)	44 (33.8)	
eGFR 60–89 - n (%)	198 (25.9)	25 (22.7)	30 (23.1)	
eGFR ≥90 - n (%)	185 (24.2)	31 (28.2)	30 (23.1)	
No kidney failure (eGFR ≥60) - n (%)	383 (50.1)	56 (50.9)	60 (46.2)	0.063
eGFR (ml/min/m2) - mean (SD)	66.89 (49.43)	71.26 (58.96)	63.78 (40.31)	0.099
**Cause of arrest:** - n (%)				0.629
Hemorrhage - n (%)	34 (4.9)	2 (2.0)	10 (8.1)	
Myocardial infarction/ischemia- n (%)	181 (26.2)	15 (14.9)	41 (33.3)	
Other - n (%)	213 (30.8)	30 (29.7)	41 (33.3)	
Primary arrhythmia - n (%)	101 (14.6)	8 (7.9)	12 (9.8)	
Respiratory insufficiency - n (%)	73 (10.5)	24 (23.8)	7 (5.7)	
Sepsis/infection - n (%)	45 (6.5)	19 (18.8)	4 (3.3)	

Stroke/thromboembolism - n (%)	45 (6.5)	3 (3.0)	8 (6.5)	
**Early interventions - n (%):**				
Witnessed arrest - n (%)	857 (80.9)	140 (77.8)	306 (85.0)	0.124
ECG monitoring - n (%)	635 (60.5)	89 (50.0)	221 (62.1)	0.163
CPR before AGA - n (%)	845 (91.0)	146 (93.6)	268 (88.2)	0.127
Defibrillated before AGA – n (%)	159 (17.9)	18 (11.9)	53 (19.0)	0.131
Ventilated before AGA - n (%)	503 (63.2)	74 (54.8)	175 (69.2)	0.199
Shockable rhythm - n (%)	247 (24.9)	29 (17.3)	90 (27.0)	0.158
Defibrillated, any - n (%)	323 (31.5)	40 (22.7)	111 (32.8)	0.151
Intubated - n (%)	473 (47.0)	100 (57.8)	177 (53.8)	0.145
Adrenaline given - n (%)	668 (65.6)	125 (72.7)	223 (66.4)	0.102
Antiarrhythmics - n (%)	139 (14.1)	17 (10.1)	48 (15.4)	0.107
Mechanical compressions – n (%)	109 (10.8)	18 (10.4)	66 (20.0)	0.180
Active temperature control – n (%)	54 (11.3)	5 (10.4)	3 (4.4)	0.173
**Status at rescue team arrival - n (%):**				
Consciousness - n (%)	214 (23.1)	18 (11.7)	57 (19.3)	0.204
Breathing - n (%)	288 (31.2)	30 (19.5)	84 (28.7)	0.181
Pulse - n (%)	309 (33.8)	36 (23.4)	89 (30.4)	0.154
**Follow-Up data - n (%):**				
Angiography - n (%)	115 (24.2)	8 (16.7)	15 (20.8)	0.124
PCI - n (%)	87 (18.2)	4 (8.3)	16 (21.9)	0.258
Pacemaker implanted - n (%)	80 (16.7)	2 (4.2)	4 (5.6)	0.281
ICD implanted - n (%)	36 (7.5)	1 (2.1)	2 (2.8)	0.172
ROSC - n (%)	520 (49.0)	64 (35.2)	142 (38.5)	0.188
Death at 30 days - n (%)	666 (62.7)	141 (77.5)	237 (64.2)	0.218
Death overall - n (%)	703 (66.2)	141 (77.5)	241 (65.3)	0.181
Discharged alive – n (%)	283 (35.6)	22 (19.0)	39 (26.5)	0.253

SD = standard deviation; IQR = interquartile range; SMD = standardized mean difference (difference between the means for the two groups divided by their mutual standard deviation. Values below 0.1 (10%) are considered inconsequential (i.e., no significant difference between the groups)). CPR = Cardiopulmonary resuscitatio*n,* PCI = Percutaneous Coronary Intervention, ICD = implantable cardioverter-defibrillator. ROSC = return of spontaneous circulation. AGA= alarm group arrival

1

2

Figure Titles and Legends

Figure 1: Characteristics of IHCA according to COVID-19 status

A: Monthly proportion of COVID-19 status among patients with IHCA, stratified on COVID-19 status. In March only cases after 15/03/2020 were included.

B: Etiology of IHCA, stratified on COVID-19 status. The y-axis shows percentages for each etiology in each group.

C: Clinical conditions 1 hour prior to IHCA, stratified on COVID-19 status. Only patients with data regarding the specific condition was included.

Figure 2: Kaplan Meier survival curves

Kaplan Meier survival curves, separately for (A)Overall, (B)Women, (C)Men, (D)Age ≥70 year, (E)Age <70 year, (F)Cancer, (G)Heart failure, (H)Diabetes, (I)Kidney failure and (J)Myocardial infarction. p= log-rank p-value. The numbers under the graphs are showing the survival in percentages. Regarding myocardial infarction acute MI is presented.

Figure 3: Odds Ratio for 30-day survival

Forest plot with the adjusted odds ratio for 30-day survival among patients with ongoing infection vs. no infection and unknown/NA vs. no infection. Stratified on overall, men,

women, age < 70 years, age ≥ 70 years, heart failure, kidney failure, diabetes, myocardial infarction and cancer. Myocardial infarction was defined as acute or previous MI.

Figure 4: Odds Ratio for 30-day survival

Forest plot with odds ratio for 30-day survival, stratified on the groups, no infection, ongoing infection and overall, all in different colors. The 95% Confidence interval is shown between the bars. X-axis has a logarithmic scale. ECG=electrocardiogram, CA=cardiac arrest, MI=myocardial infarction. CI=confidence interval.

Figure 1: Characteristics of IHCA according to COVID-19 status

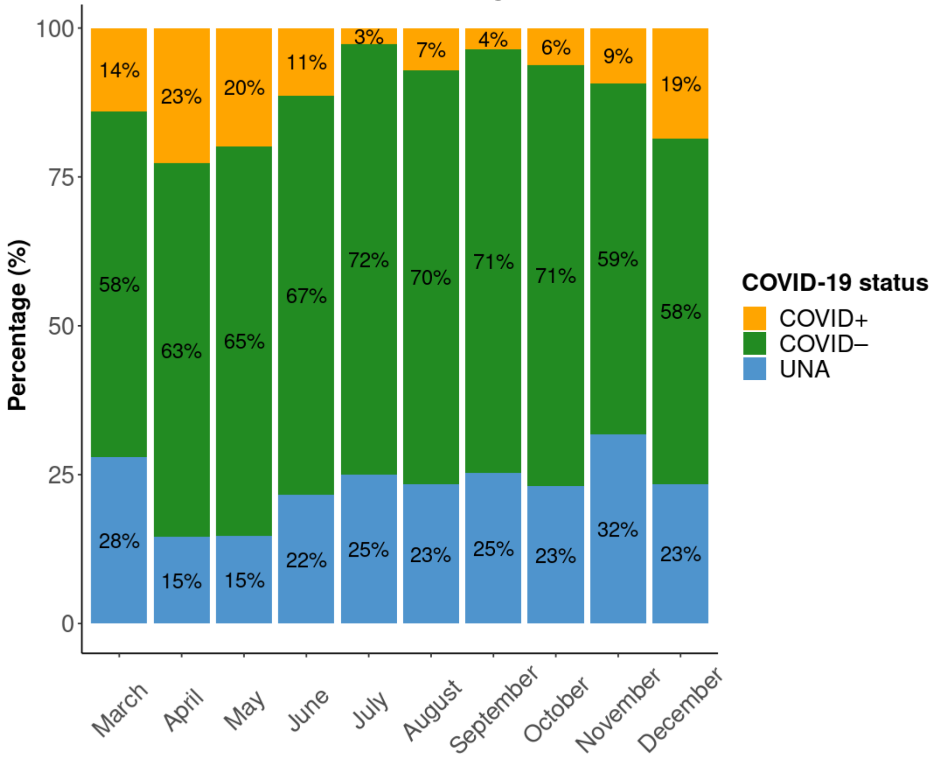

Figure 1A: Monthly proportion of COVID-19 status among patients with IHCA, stratified on COVID-19 status. In March only cases after 15/03/2020 were included.

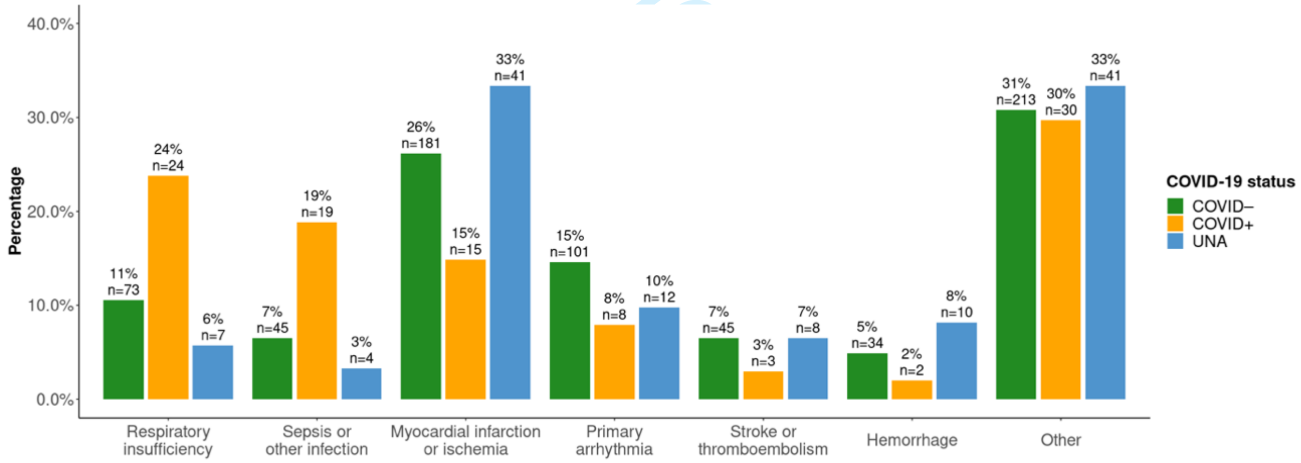

Figure 1B: Etiology of IHCA, stratified on COVID-19 status. The y-axis shows percentages for each etiology in each group.

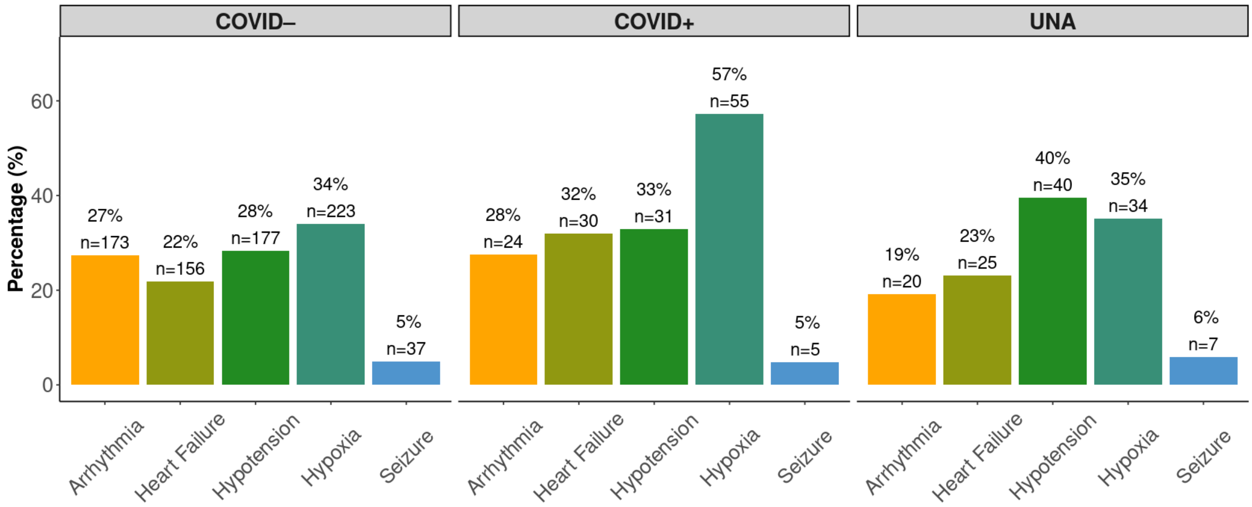

Figure 1C: Clinical conditions 1 hour prior to IHCA, stratified on COVID-19 status. Only patients with data regarding the specific condition was included.

For peer review only

Figure 2: Kaplan Meier survival curves

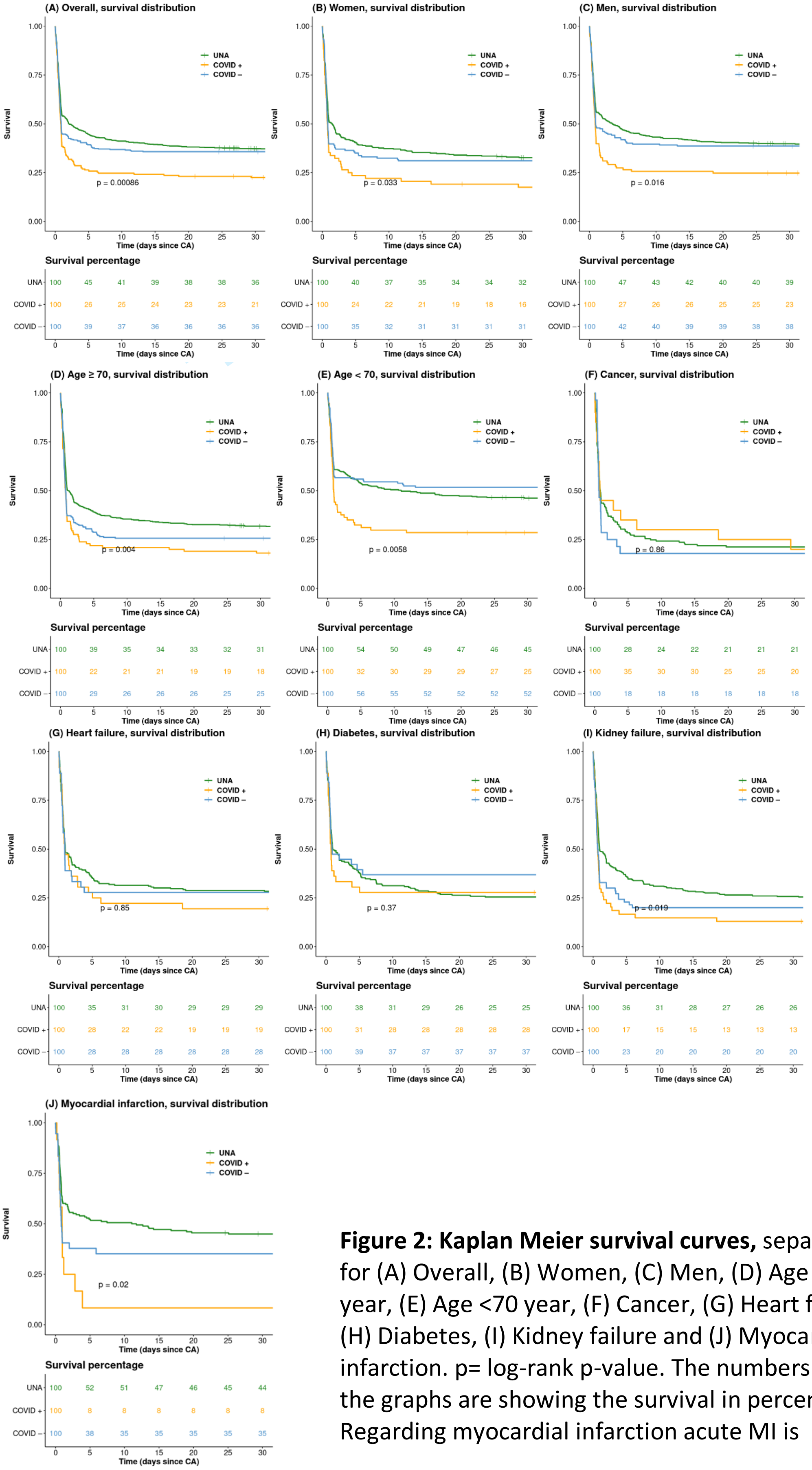

Figure 2: Kaplan Meier survival curves, separately for (A) Overall, (B) Women, (C) Men, (D) Age ≥70 year, (E) Age <70 year, (F) Cancer, (G) Heart failure, (H) Diabetes, (I) Kidney failure and (J) Myocardial infarction. p= log-rank p-value. The numbers under the graphs are showing the survival in percentages. Regarding myocardial infarction acute MI is

Figure 3: Odds Ratio for 30-day survival

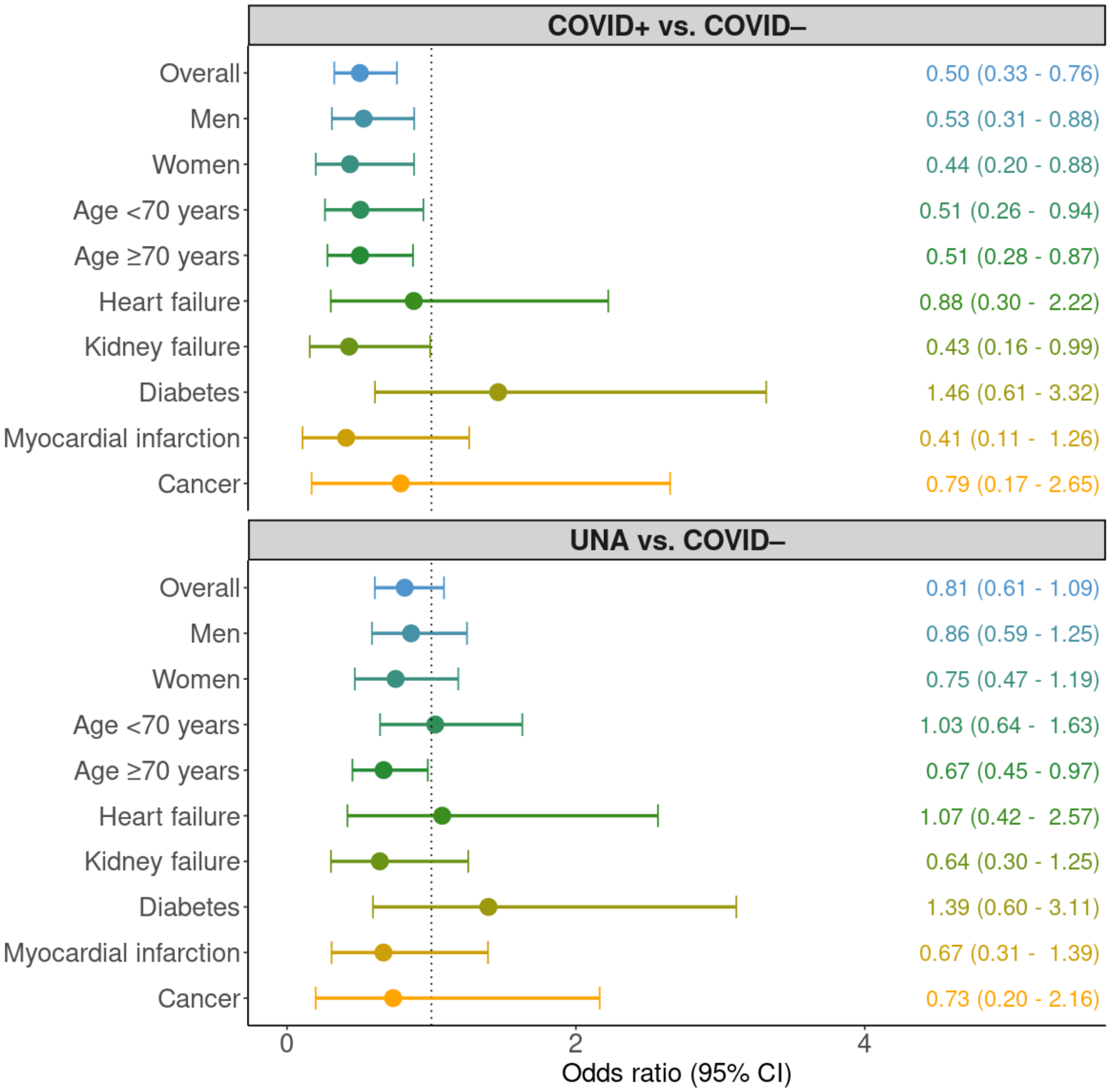

Figure 3: Forest plot with the adjusted odds ratio for 30-day survival among patients with ongoing infection vs. no infection and unknown/NA vs. no infection. Stratified on overall, men, women, age < 70 years, age ≥ 70 years, heart failure, kidney failure, diabetes, myocardial infarction and cancer. Myocardial infarction was defined as acute or previous MI.

Figure 4: Odds Ratio for 30-day survival

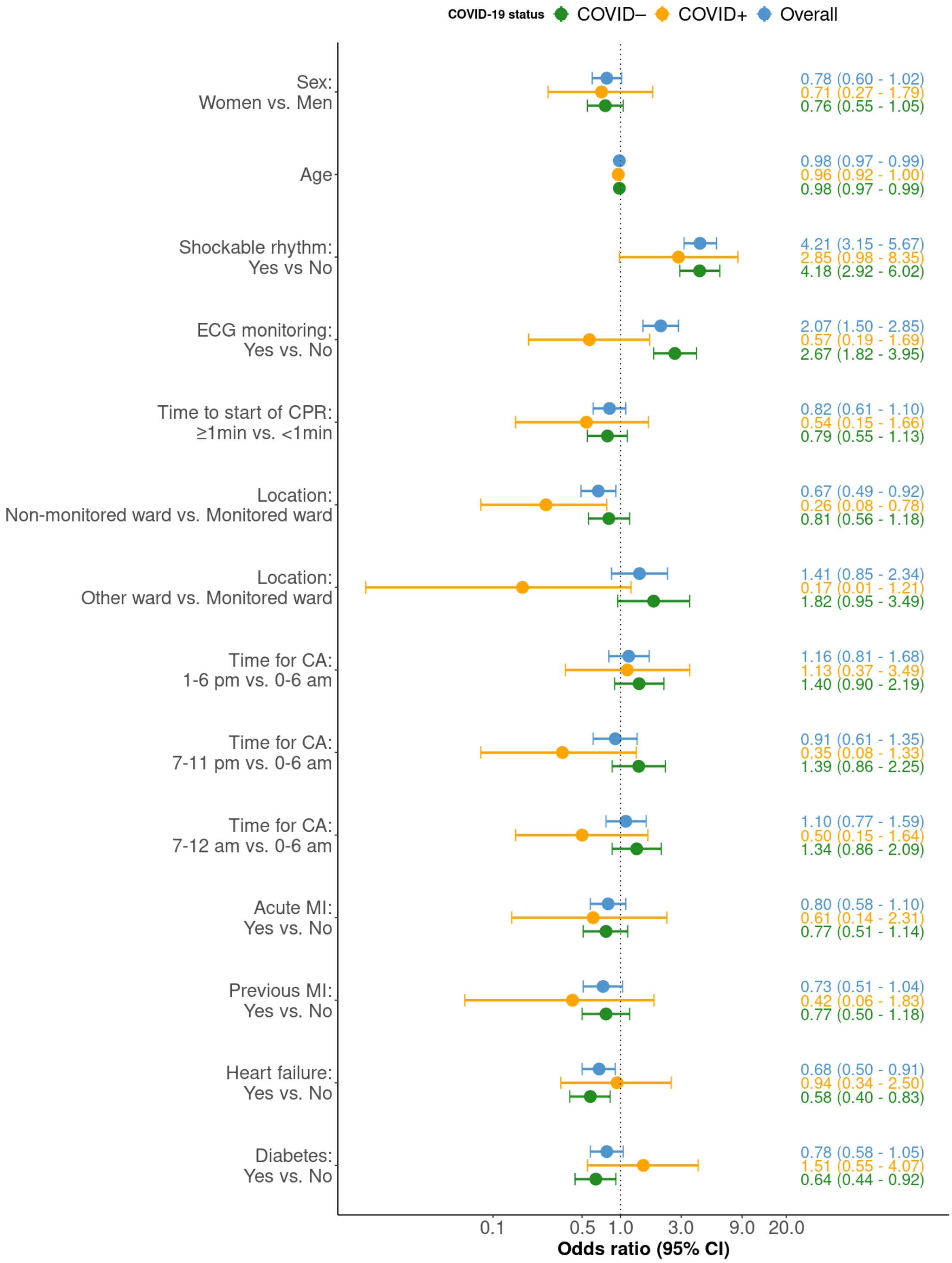

Figure 4: Forest plot with odds ratio for 30-day survival, stratified on the groups, no infection, ongoing infection and overall, all in different colors. The 95% Confidence interval is shown between the bars. X-axis has a logarithmic scale. ECG= electrocardiogram, CA= cardiac arrest, MI= myocardial infarction. CI= confidence interval.

Supplementary figures and tables

Characteristics and Outcomes in Patients with COVID-19 and In-Hospital Cardiac Arrest

For peer review only

Supplementary Table 1: Characteristics of COVID+ patients with IHCA in relation to sex.

Supplementary Table 1: Characteristics of 181 COVID+ patients with IHCA during the COVID-19 pandemic in relation to sex. One COVID+ patient had missing data on sex.

variables	Men	Women	SMD
**n**	113	68	
**Demographics:**			
Age - mean (SD)	71.39 (10.75)	70.35 (14.87)	0.080
**Location of cardiac arrest - n (%):**			0.249
Coronary care unit	7 (6.2)	7 (10.3)	
Intensive care unit	15 (13.3)	10 (14.7)	
Operational room	0 (0.0)	0 (0.0)	
Emergency room	17 (15.0)	11 (16.2)	
Outpatient lab, radiology	4 (3.5)	3 (4.4)	
Cathlab	6 (5.3)	2 (2.9)	
Intermediate care unit	11 (9.7)	4 (5.9)	
Regular ward	52 (46.0)	30 (44.1)	
Other	1 (0.9)	1 (1.5)	
**Critical times - median (IQR):**			
Time to alert – median (IQR)	1.00 [1.00, 1.00]	1.00 [1.00, 1.00]	0.256
Time to CPR - median (IQR)	0.00 [0.00, 0.00]	0.00 [0.00, 1.00]	0.031
Time to defibrillation - median	2.00 [1.00, 5.00]	2.00 [1.00, 2.00]	0.009
**Comorbidities - n (%):**			
MI, ongoing - n (%)	5 (7.6)	7 (21.2)	0.396
MI, previous - n (%)	11 (16.4)	2 (4.7)	0.391
Stroke, ongoing - n (%)	4 (5.8)	0 (0.0)	0.351
Stroke, previous - n (%)	5 (7.0)	2 (4.7)	0.102
Cancer, any - n (%)	13 (18.8)	6 (14.0)	0.132
Diabetes - n (%)	24 (33.3)	12 (27.9)	0.118
Heart failure - n (%)	23 (33.8)	13 (32.5)	0.028
Ejection fraction (%) - mean (SD)	44.84 (12.22)	49.31 (10.56)	0.392
EF <50% - n (%)	19 (51.4)	7 (43.8)	0.153
**Kidney function category - n (%):**			0.357
eGFR <30	16 (22.9)	6 (15.0)	
eGFR 30–59	17 (24.3)	15 (37.5)	
eGFR 60–89	18 (25.7)	7 (17.5)	
eGFR ≥90	19 (27.1)	12 (30.0)	
No kidney failure (eGFR ≥60)	37 (52.9)	19 (47.5)	0.107
eGFR (ml/min/m2) - mean (SD)	72.72 (65.75)	68.70 (45.34)	0.071
**Cause of arrest - n (%):**			0.920
Hemorrhage	1 (1.5)	1 (2.9)	
Myocardial infarction/ischemia	7 (10.6)	8 (23.5)	
Other	18 (27.3)	12 (35.3)	
Primary arrhythmia	3 (4.5)	5 (14.7)	
Respiratory insufficiency	17 (25.8)	7 (20.6)	

For peer review only - http://bmjopen.bmj.com/site/about/guidelines.xhtml

Sepsis / infection	18 (27.3)	1 (2.9)	
Stroke / thromboembolism	2 (3.0)	0 (0.0)	
**Early interventions - n (%):**			
Witnessed arrest - n (%)	86 (76.8)	53 (79.1)	0.056
ECG monitoring - n (%)	56 (50.5)	33 (50.0)	0.009
CPR before AGA - n (%)	90 (92.8)	55 (94.8)	0.085
Defibrillated before AGA - n (%)	13 (13.8)	5 (8.9)	0.155
Ventilated before AGA- n (%)	49 (56.3)	25 (53.2)	0.063
Shockable rhythm - n (%)	22 (20.8)	7 (11.5)	0.254
Defibrillated, any - n (%)	29 (26.4)	11 (16.9)	0.231
Intubated - n (%)	61 (57.0)	38 (58.5)	0.029
Adrenaline given - n (%)	76 (70.4)	48 (76.2)	0.132
Antiarrhythmics - n (%)	11 (10.4)	6 (9.7)	0.023
Mechanical compressions - n (%)	12 (10.9)	5 (8.1)	0.097
Active temperature control - n (%)	2 (6.1)	3 (20.0)	0.423
**Status at rescue team arrival - n**			
Consciousness - n (%)	11 (11.3)	6 (10.7)	0.020
Breathing - n (%)	18 (18.6)	11 (19.6)	0.028
Pulse - n (%)	22 (22.7)	13 (23.2)	0.013
**Follow-Up data - n (%):**			
Angiography - n (%)	4 (12.1)	4 (26.7)	0.374
PCI - n (%)	2 (6.1)	2 (13.3)	0.248
Pacemaker implanted - n (%)	0 (0.0)	2 (13.3)	0.555
ICD implanted - n (%)	0 (0.0)	1 (6.7)	0.378
ROSC - n (%)	40 (35.4)	24 (35.3)	0.002
Death at 30 days - n (%)	85 (75.2)	56 (82.4)	0.175
Death overall - n (%)	85 (75.2)	56 (82.4)	0.175
Discharged alive - n (%)	16 (22.2)	6 (14.0)	0.216

SD = standard deviation; IQR = interquartile range; SMD = standardized mean difference (difference between the means for the two groups divided by their mutual standard deviation. Values below 0.1 (10%) are considered inconsequential (i.e., no significant difference between the groups)). CPR = cardiopulmonary resuscitatio*n*, PCI = percutaneous coronary intervention, ICD = implantable cardioverter-defibrillator. ROSC = return of spontaneous circulation. AGA= alarm group arrival.

Supplementary Figure 1: Missing data before and after imputation with MICE

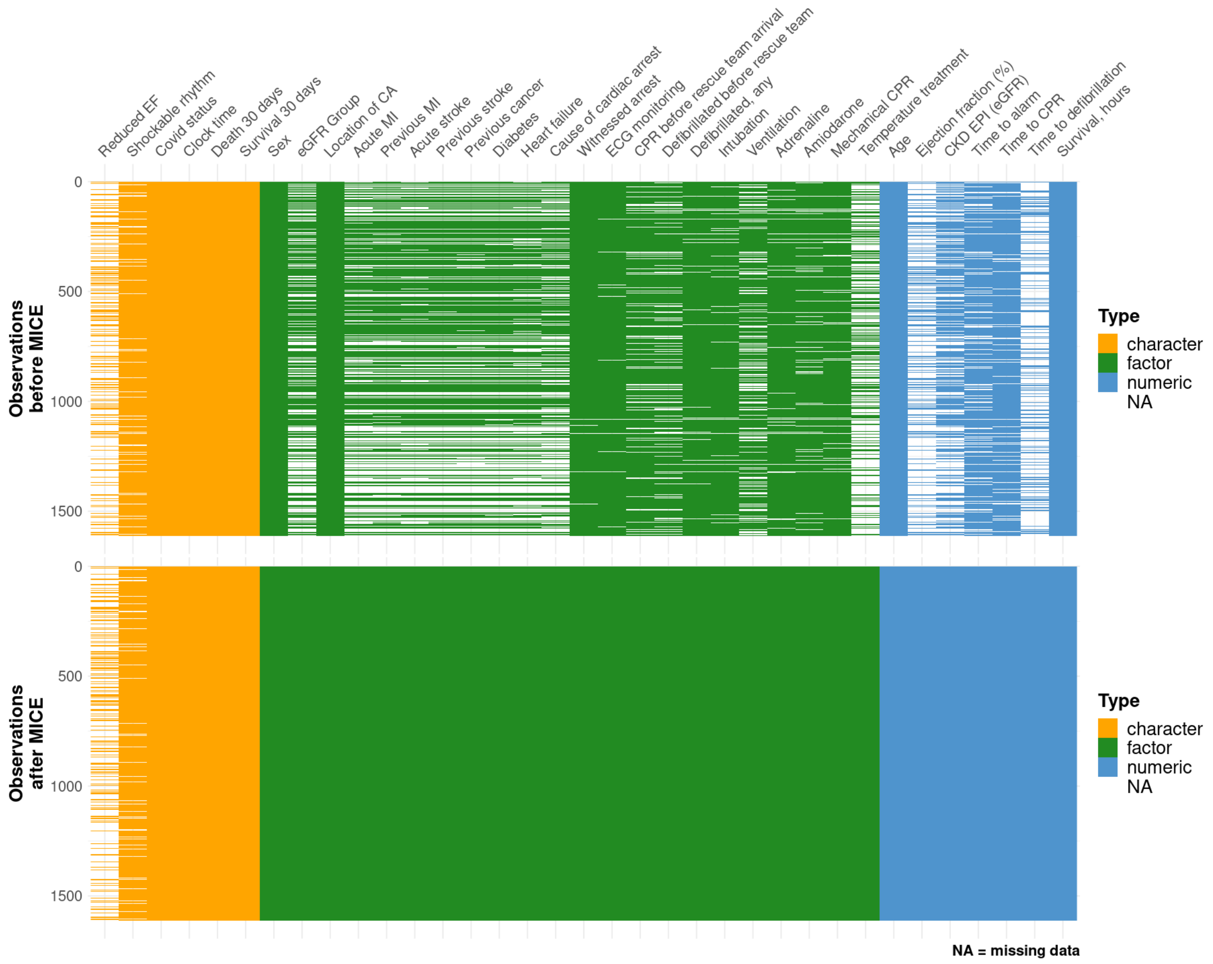

Supplementary Figure 1: Missing data before and after imputation with MICE. A graphical view of the entire dataset is printed. Each column (variable) is depicted at the top and column color depicts type of variable. Each patient represents a row and white gaps indicate a missing data entry.

Supplementary Figure 2: Flow chart

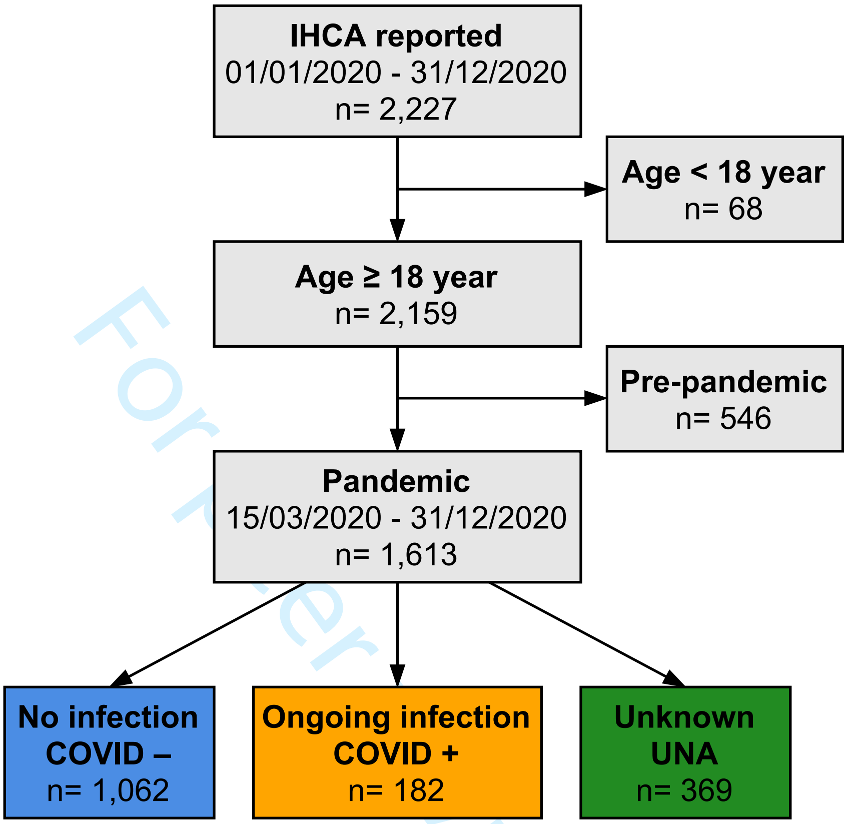

Supplementary Figure 2: Flow chart of the study population. Patients who were less than 18 year of age, and cases occurring in the pre-pandemic period were excluded.

Supplementary Figure 3: Information on COVID-19 status during the study period.

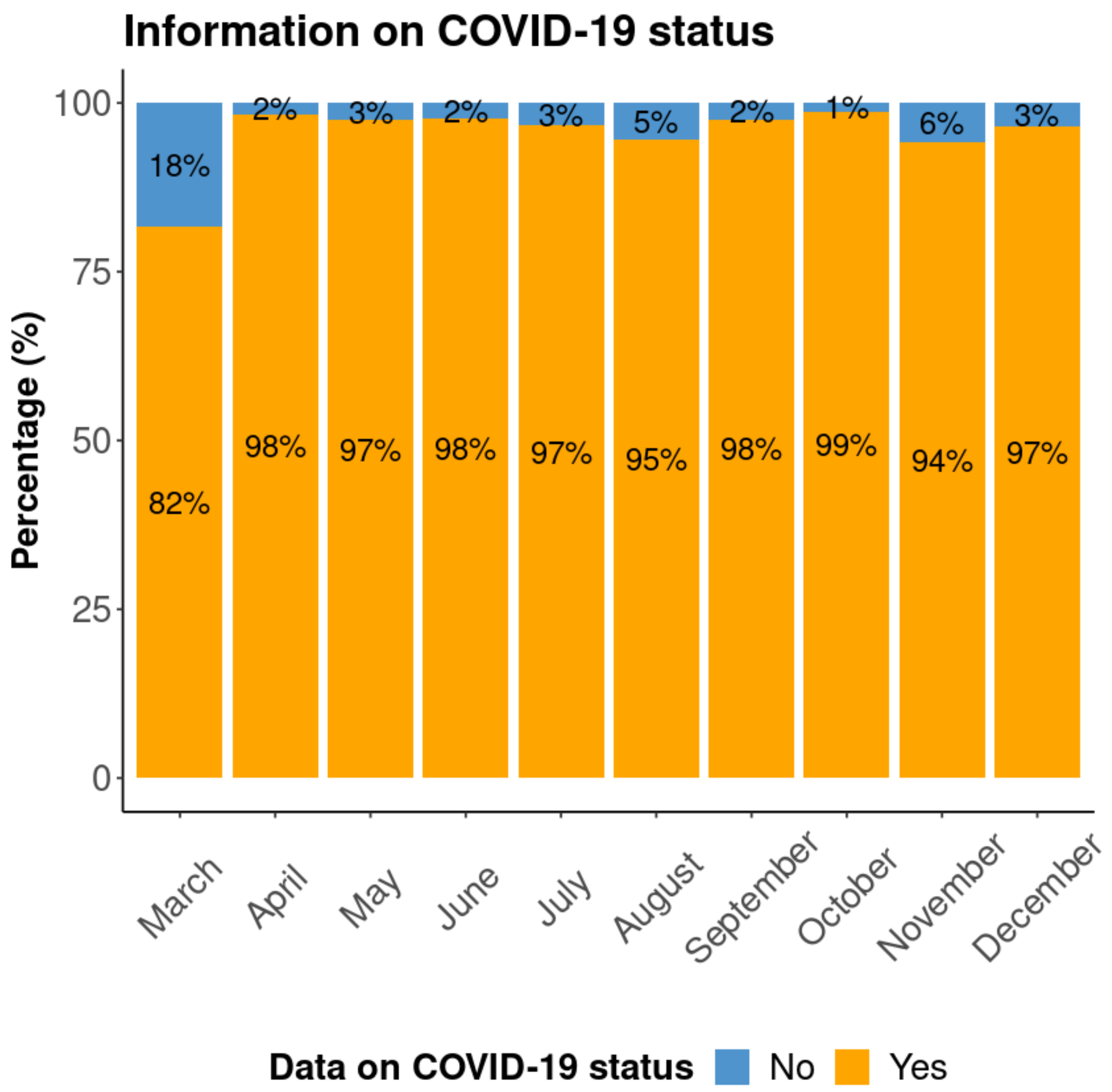

Supplementary Figure 3: Information on COVID-19 status during the study period. No equals missing data, i.e. no information on COVID-19 status available. Yes equals, COVID +, COVID – or Unknown. In March only cases after 15/03/2020 were included.

Supplementary Figure 4: Distribution of age

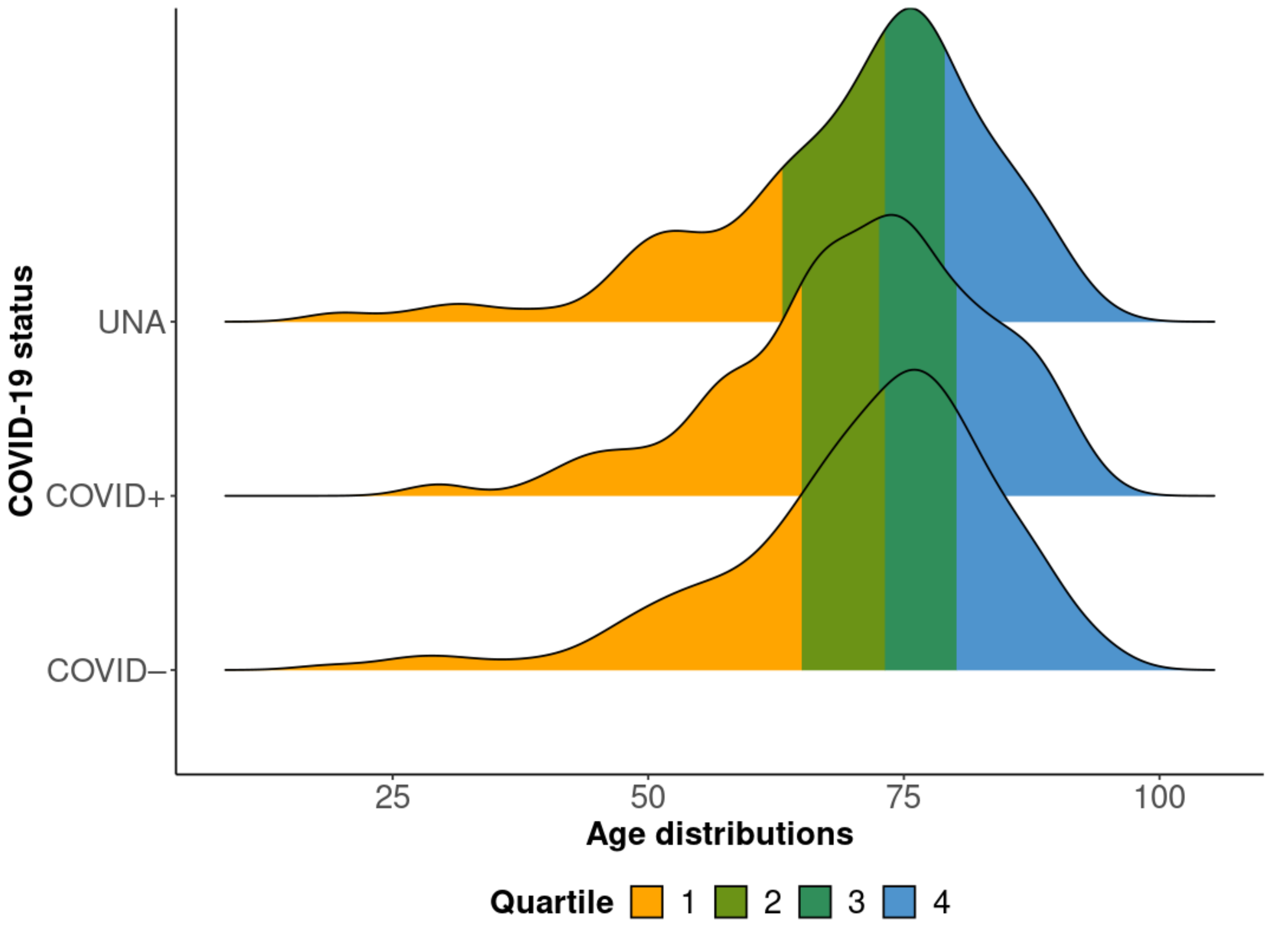

Supplementary Figure 4: Distribution of age in relation to COVID-19 status.

Supplementary Figure 5: Etiology of IHCA, according to sex

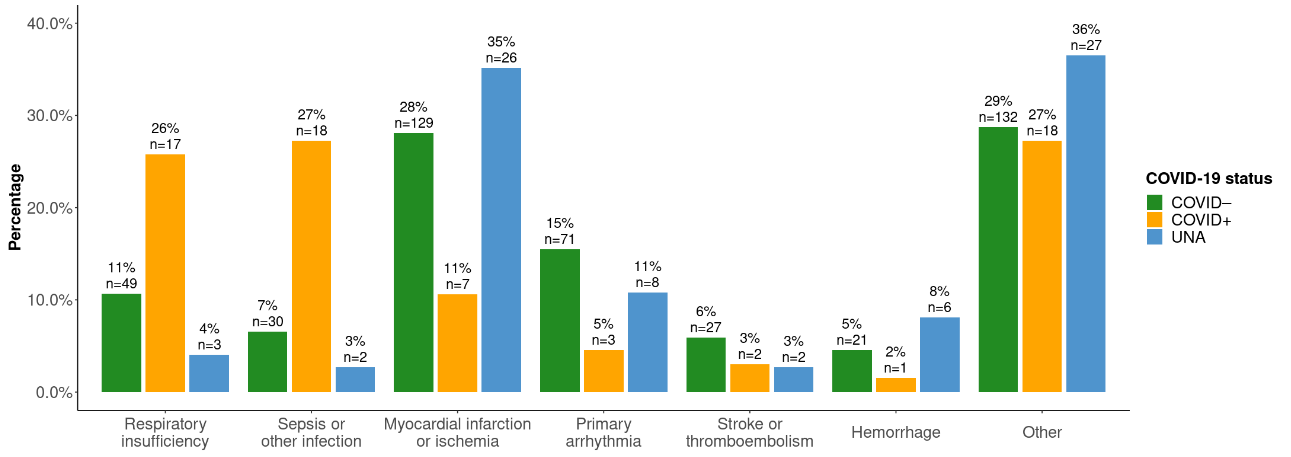

Supplementary Figure 5A: Etiology of IHCA, men only.

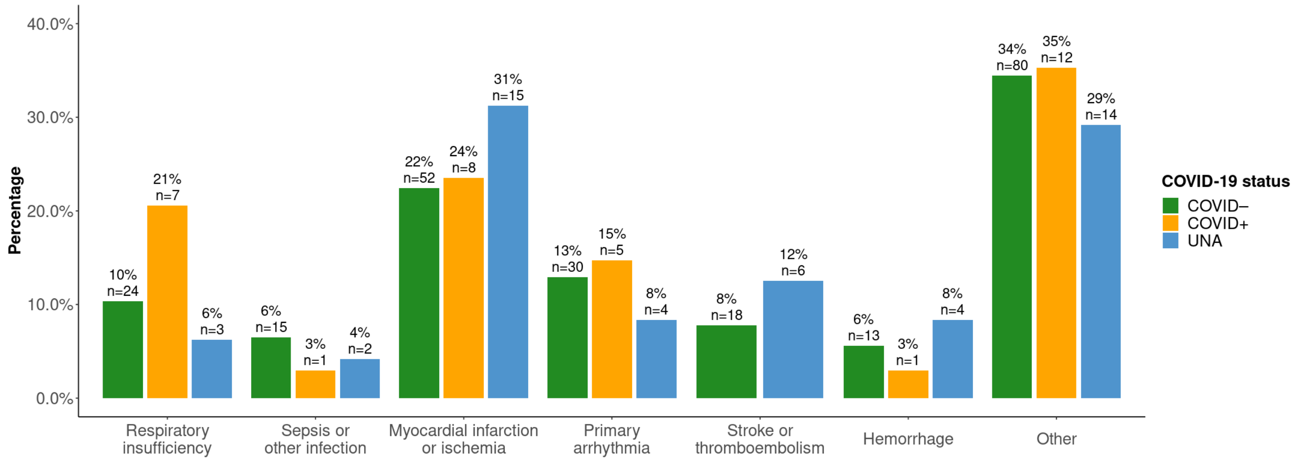

Supplementary Figure 5B: Etiology of IHCA, women only.

For peer review only - http://bmjopen.bmj.com/site/about/guidelines.xhtml

Supplementary Figure 6: Conditions preceding IHCA, according to sex

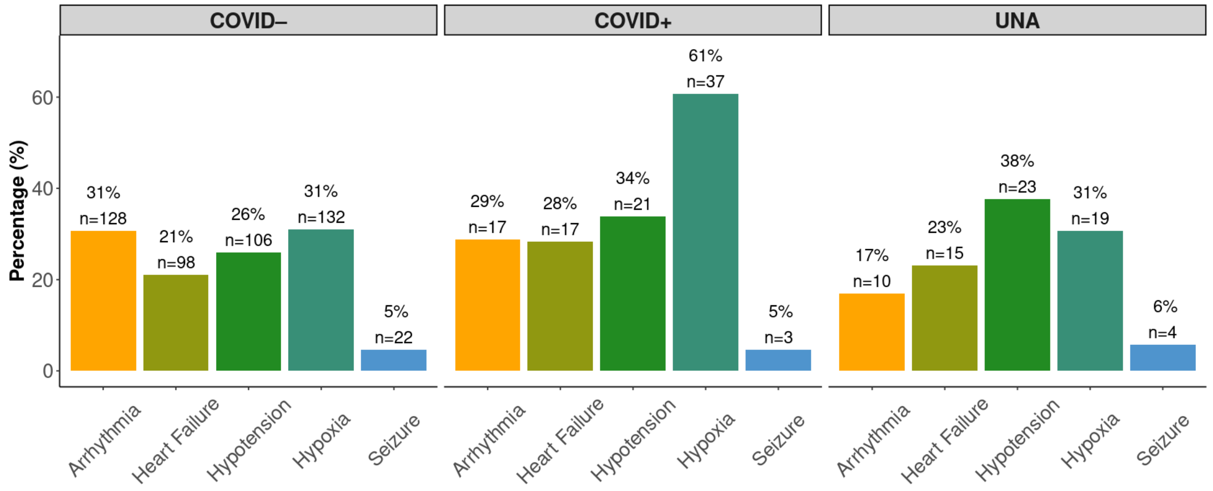

Supplementary Figure 6A: Conditions preceding IHCA, men only.

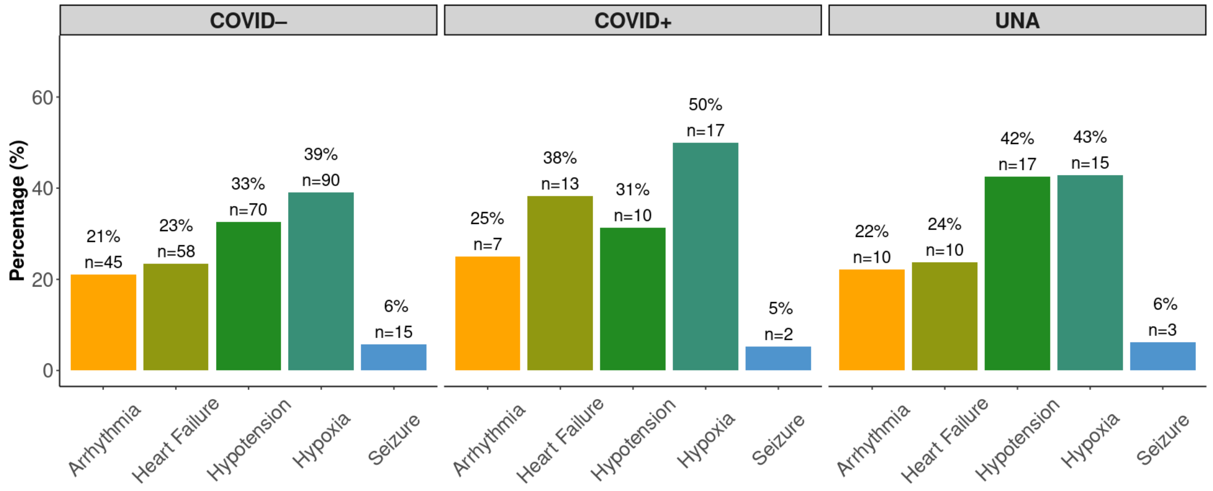

Supplementary Figure 6B: Conditions preceding IHCA, women only.

Supplementary Figure 7: Cox adjusted survival curve for the overall population

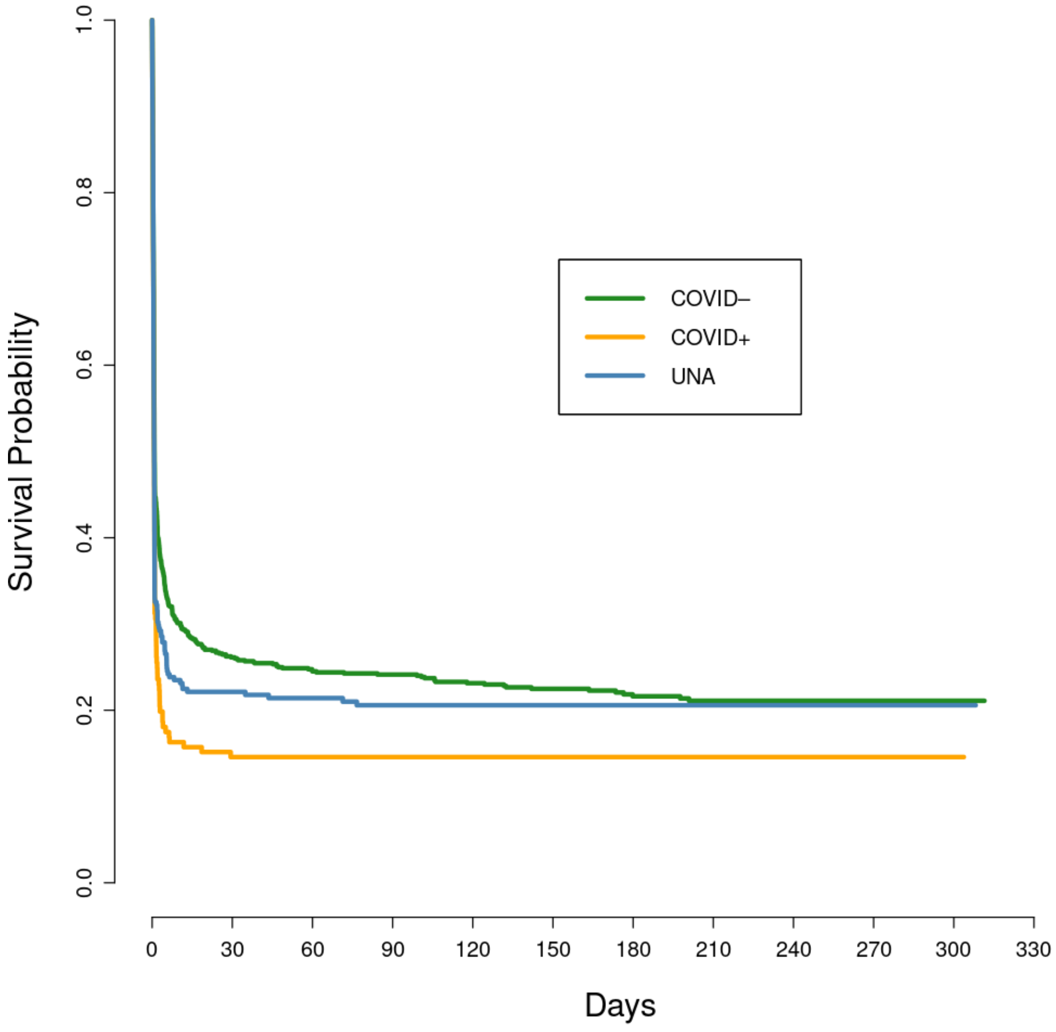

Supplementary Figure 7: Cox adjusted survival curve for the overall population, stratified on COVID-19 status.

only

STROBE Statement—checklist of items that should be included in reports of observational studies

	Item No	Recommendation	Page No
**Title and abstract**	1	(*a*) Indicate the study's design with a commonly used term in the title or the abstract	1
		(*b*) Provide in the abstract an informative and balanced summary of what was done and what was found	2-3
**Introduction**			
Background/rationale	2	Explain the scientific background and rationale for the investigation being reported	2
Objectives	3	State specific objectives, including any prespecified hypotheses	2
**Methods**			
Study design	4	Present key elements of study design early in the paper	2
Setting	5	Describe the setting, locations, and relevant dates, including periods of recruitment, exposure, follow-up, and data collection	2
Participants	6	(*a*) *Cohort study*—Give the eligibility criteria, and the sources and methods of selection of participants. Describe methods of follow-up<br>*Case-control study*—Give the eligibility criteria, and the sources and methods of case ascertainment and control selection. Give the rationale for the choice of cases and controls<br>*Cross-sectional study*—Give the eligibility criteria, and the sources and methods of selection of participants	2
		(*b*) *Cohort study*—For matched studies, give matching criteria and number of exposed and unexposed<br>*Case-control study*—For matched studies, give matching criteria and the number of controls per case	2
Variables	7	Clearly define all outcomes, exposures, predictors, potential confounders, and effect modifiers. Give diagnostic criteria, if applicable	5-6
Data sources/ measurement	8*	For each variable of interest, give sources of data and details of methods of assessment (measurement). Describe comparability of assessment methods if there is more than one group	*5*
Bias	9	Describe any efforts to address potential sources of bias	3-4
Study size	10	Explain how the study size was arrived at	5
Quantitative variables	11	Explain how quantitative variables were handled in the analyses. If applicable, describe which groupings were chosen and why	5-6
Statistical methods	12	(*a*) Describe all statistical methods, including those used to control for confounding	6-7
		(*b*) Describe any methods used to examine subgroups and interactions	6-7
		(*c*) Explain how missing data were addressed	6-7
		(*d*) *Cohort study*—If applicable, explain how loss to follow-up was addressed<br>*Case-control study*—If applicable, explain how matching of cases and controls was addressed<br>*Cross-sectional study*—If applicable, describe analytical methods taking account of sampling strategy	6-7
		(*e*) Describe any sensitivity analyses	6-7

Continued on next page

For peer review only - http://bmjopen.bmj.com/site/about/guidelines.xhtml
For peer review only

**Results**			
Participants	13*	(a) Report numbers of individuals at each stage of study—eg numbers potentially eligible, examined for eligibility, confirmed eligible, included in the study, completing follow-up, and analysed	7
		(b) Give reasons for non-participation at each stage	7
		(c) Consider use of a flow diagram	Supplementary material
Descriptive data	14*	(a) Give characteristics of study participants (eg demographic, clinical, social) and information on exposures and potential confounders	7-8
		(b) Indicate number of participants with missing data for each variable of interest	7-8
		(c) *Cohort study*—Summarise follow-up time (eg, average and total amount)	7
Outcome data	15*	*Cohort study*—Report numbers of outcome events or summary measures over time	*8-9*
		Case-control study—Report numbers in each exposure category, or summary measures of exposure	
		Cross-sectional study—Report numbers of outcome events or summary measures	
Main results	16	(*a*) Give unadjusted estimates and, if applicable, confounder-adjusted estimates and their precision (eg, 95% confidence interval). Make clear which confounders were adjusted for and why they were included	7-10
		(*b*) Report category boundaries when continuous variables were categorized	
		(*c*) If relevant, consider translating estimates of relative risk into absolute risk for a meaningful time period	
Other analyses	17	Report other analyses done—eg analyses of subgroups and interactions, and sensitivity analyses	Supplementary material
**Discussion**			
Key results	18	Summarise key results with reference to study objectives	11
Limitations	19	Discuss limitations of the study, taking into account sources of potential bias or imprecision. Discuss both direction and magnitude of any potential bias	13-14
Interpretation	20	Give a cautious overall interpretation of results considering objectives, limitations, multiplicity of analyses, results from similar studies, and other relevant evidence	11-14
Generalisability	21	Discuss the generalisability (external validity) of the study results	14
**Other information**			
Funding	22	Give the source of funding and the role of the funders for the present study and, if applicable, for the original study on which the present article is based	14

*Give information separately for cases and controls in case-control studies and, if applicable, for exposed and unexposed groups in cohort and cross-sectional studies.

Note: An Explanation and Elaboration article discusses each checklist item and gives methodological background and published examples of transparent reporting. The STROBE checklist is best used in conjunction with this article (freely

For peer review only - http://bmjopen.bmj.com/site/about/guidelines.xhtml

available on the Web sites of PLoS Medicine at http://www.plosmedicine.org/, Annals of Internal Medicine at http://www.annals.org/, and Epidemiology at http://www.epidem.com/). Information on the STROBE Initiative is available at www.strobe-statement.org.

For peer review only

For peer review only - http://bmjopen.bmj.com/site/about/guidelines.xhtml

BMJ Open

A Cohort Study of the Characteristics and Outcomes in Patients with COVID-19 and In-Hospital Cardiac Arrest

Journal:	*BMJ Open*
Manuscript ID	bmjopen-2021-054943.R1
Article Type:	Original research
Date Submitted by the Author:	30-Sep-2021
Complete List of Authors:	Holm, Astrid; University of Gothenburg, Department of Molecular and Clinical Medicine<br>Jerkeman, Matilda; University of Gothenburg, Department of Molecular and Clinical Medicine<br>Sultanian, Pedram; University of Gothenburg, Department of Molecular and Clinical Medicine<br>Lundgren, Peter; University of Gothenburg, Department of Molecular and Clinical Medicine; Kalmar County Hospital, Department of Internal Medicine<br>Ravn-Fischer, Annica; University of Gothenburg, Department of Molecular and Clinical Medicine<br>Israelsson, Johan; Kalmar County Hospital, Department of Internal Medicine; Linnaeus University, Faculty of Health and Life Sciences<br>Giesecke, Jasna; Danderyd University Hospital<br>Herlitz, Johan; University of Borås, Centre for Prehospital Research<br>Rawshani, Araz; University of Gothenburg, Department of Molecular and Clinical Medicine
**Primary Subject Heading**:	Cardiovascular medicine
Secondary Subject Heading:	Cardiovascular medicine, Infectious diseases
Keywords:	COVID-19, CARDIOLOGY, Adult cardiology < CARDIOLOGY, Coronary heart disease < CARDIOLOGY

SCHOLARONE™ Manuscripts

I, the Submitting Author has the right to grant and does grant on behalf of all authors of the Work (as defined in the below author licence), an exclusive licence and/or a non-exclusive licence for contributions from authors who are: i) UK Crown employees; ii) where BMJ has agreed a CC-BY licence shall apply, and/or iii) in accordance with the terms applicable for US Federal Government officers or employees acting as part of their official duties; on a worldwide, perpetual, irrevocable, royalty-free basis to BMJ Publishing Group Ltd ("BMJ") its licensees and where the relevant Journal is co-owned by BMJ to the co-owners of the Journal, to publish the Work in this journal and any other BMJ products and to exploit all rights, as set out in our licence.

The Submitting Author accepts and understands that any supply made under these terms is made by BMJ to the Submitting Author unless you are acting as an employee on behalf of your employer or a postgraduate student of an affiliated institution which is paying any applicable article publishing charge ("APC") for Open Access articles. Where the Submitting Author wishes to make the Work available on an Open Access basis (and intends to pay the relevant APC), the terms of reuse of such Open Access shall be governed by a Creative Commons licence – details of these licences and which Creative Commons licence will apply to this Work are set out in our licence referred to above.

Other than as permitted in any relevant BMJ Author's Self Archiving Policies, I confirm this Work has not been accepted for publication elsewhere, is not being considered for publication elsewhere and does not duplicate material already published. I confirm all authors consent to publication of this Work and authorise the granting of this licence.

For peer review only

A Cohort Study of the Characteristics and Outcomes in Patients with COVID-19 and In-Hospital Cardiac Arrest

Astrid Holm, MD[1], Matilda Jerkeman, MD[1], Pedram Sultanian, MD[1], Peter Lundgren, MD, PhD[1,3,7], Annica Ravn-Fischer, MD, PhD, Docent[1], Johan Israelsson, PhD[3,4], Jasna Giesecke, RN[5], Johan Herlitz, MD, PhD[2,6], Araz Rawshani, MD, PhD[1,2]

Affiliations:

[1]Department of Molecular and Clinical Medicine, Institute of Medicine, Sahlgrenska Academy, University of Gothenburg, Sweden

[2]The Swedish Registry for Cardiopulmonary Resuscitation, Centre of Registries, Västra Götaland County, Sweden

[3]Department of Internal Medicine, Division of Cardiology, Kalmar County Hospital, Region Kalmar County, Sweden

[4]Faculty of Health and Life Sciences, Linnaeus University, Kalmar, Sweden

[5]RN, Lead CPR coordinator, Clinicum- Centre for clinical skills, interprofessional education and advanced medical simulation, Danderyd University Hospital, Stockholm, Sweden

[6]Prehospen – Centre for Prehospital Research, University of Borås, Borås, Sweden

[7]Region Västra Götaland, Sahlgrenska University Hospital, Department of Cardiology, Gothenburg, Sweden

Contact information:

Astrid Holm

Email: astrid.holm@gu.se

Phone: +358 400707722

Manuscript word count: 3442

Key Points

Question: What are the Characteristics, causes and outcomes in patients with COVID-19 who suffer an in-hospital cardiac arrest (IHCA)?

Findings: In the registry-based observational study we found that during pandemic peaks, up to one fourth of all IHCAs are complicated by COVID-19, and these patients have halved chance of survival.

Meaning: The survival rate of patients with COVID-19 associated IHCA is low with women displaying the worst outcomes.

Abstract

Objective: We studied characteristics, survival, causes of cardiac arrest, conditions preceding cardiac arrest, predictors of survival, and trends in the prevalence of COVID-19 among IHCA cases. Data on characteristics and outcomes in patients with COVID-19 who suffer an in-hospital cardiac arrest (IHCA) is scarce.

Design and setting: Registry-based observational study.

Participants: We studied all cases of IHCA receiving CPR (≥18 years of age) in the Swedish Registry for Cardiopulmonary Resuscitation from 15/03/2020 to 31/12/2020. A total of 1613

patients were included and divided into the following groups: ongoing infection (**COVID+;** n=182), no infection (**COVID–;** n=1062) and unknown/not assessed (**UNA**; n=369).

Main outcomes and measures: We studied monthly trends in proportions of COVID-19 associated IHCAs, causes of IHCA in relation to COVID-19 status, clinical conditions preceding the cardiac arrest and predictors of survival.

Results: The rate of COVID+ patients suffering an IHCA increased to 23% during the first pandemic wave (April), then abated to 3% in July, and then increased to 19% during the second wave (December). Among COVID+ cases, 43% had respiratory insufficiency or infection as the underlying cause of the cardiac arrest, compared to 18% among COVID– cases. The most common clinical sign preceding cardiac arrest was hypoxia (57%) among COVID+ cases. Odds ratio for 30-day survival for COVID+ cases was 0.50 (95% CI 0.33-0.76) compared with COVID– cases.

Conclusion: During pandemic peaks, up to one fourth of all IHCAs are complicated by COVID-19, and these patients have halved chance of survival, with women displaying the worst outcomes.

Article Summary

Strengths and limitations of this study

- A major strength of our study is that it includes all IHCAs in Sweden which were reported to the Swedish Registry for Cardiopulmonary Resuscitation.
- The sample recorded in the Swedish Registry for Cardiopulmonary Resuscitation is unbiased since all hospitals participate in the registry and all hospitals report data on COVID-19 status
- A limitation is that we do not know the severity of the COVID-19 infection, and we do not know if COVID-19 was the main reason for admission to hospital.
- Our study only includes IHCAs receiving CPR which leaves out all other patients with IHCA, e.g with a Do Not Attempt Resuscitation order.
- It is important to stress the fact that our regression model that included only COVID-19 cases must be interpreted with caution due to the large number of predictors in the model, which had relatively few patients.

Introduction

The COVID-19 pandemic has, as of May 1st 2021 infected over 159,000,000 persons and lead to the demise of over 3,321,000 individuals(1).The Swedish Public Health Authority declared on March 16th 2020 that community spread of COVID-19 had commenced, and COVID-19 is now the third leading cause of death in Sweden(2, 3).

Multiple studies have showed that in-hospital cardiac arrest (IHCA) among patients with COVID-19 is associated with poor survival(4-7). In a study from the U.S. with 260 patients hypoxia was the main cause to Cardiac arrest among over 40% of the patients with COVID-19 and IHCA (6).We studied IHCA in the Swedish Registry for Cardiopulmonary Resuscitation (SRCR) and showed a 2.3-fold increase in 30-day mortality among cases with COVID-19 compared to pre-pandemic cases and this was mainly driven by a 9-fold increase in mortality among women with COVID-19. During the study period no case of IHCA with COVID-19 was discharged alive from the hospital(8).

The current study expands our previous investigation, including more patients, longer follow-up and emphasizes the causes of cardiac arrest in COVID-19, predictors of survival, coexisting conditions, and trends in the prevalence of COVID-19 among IHCA cases.

Methods

Data sources

The study is a registry-based observational study with data obtained from the SRCR during the time period 15/03/2020 to 31/12/2020.

The SRCR is a national quality registry and has included IHCA cases since 2005. The data is collected by trained nurses who report patient data using a web-based protocol. The registry has previously been described in detail(9). Vital status was obtained from the Swedish Population Registry and the last day of follow up was 31/12/2020.

Study population

The study population included all patients ≥18 years of age suffering from IHCA and receiving CPR throughout Sweden during the period 15/03/2020 to 31/12/2020. We used 15th of March as the start date of the pandemic as the Swedish Public Health Authority declared on March 16th 2020 that COVID-19 was community spread in Sweden(3). On 1st of April the SRCR started collecting data about COVID-19 status, and retrospectively identified 60 patients with COVID-19 who suffered IHCA during March (they were included in the study). Patients were divided into the following three groups: ongoing infection (COVID+; n=182), no infection (COVID–; n=1062) and unknown/not assessed (UNA; n=369). COVID+ was

defined as patients registered with an ongoing COVID-19 infection, suspected ongoing infection or patients with a recent infection(n=29).

Variable definitions

In SRCR a patient with cardiac arrest was defined as an unconscious patient with no or abnormal breathing, in whom resuscitation or defibrillation was attempted. IHCA was defined as cardiac arrest in patients admitted to the hospital.

With regards to previous coexisting conditions heart failure was defined as any heart failure described before cardiac arrest. Kidney failure was defined as estimated glomerular filtration rate (eGFR) below 60 ml/min/1.73 m^2, calculated using the highest creatinine before cardiac arrest with Chronic Kidney Disease Epidemiology Collaboration (CKD-EPI) formula based on sex, age and creatinine. The SRCR records data on the highest creatinine levels analyzed up to six months prior to CA. Diabetes was defined as any diabetes diagnosis, regardless of type. Cancer was defined as any previously known cancer. Acute myocardial infarction (MI) was defined as an MI within 72 hours of CA. **Previous** myocardial infarction was defined as MI occurring earlier than 72 hours preceding the CA.

Regarding clinical conditions one hour prior to CA, arrhythmia was defined as any arrhythmia, hypoxia was defined as an oxygen saturation below 90%, hypotension was

defined as systolic blood pressure below 90 mmHg, seizure was defined as any seizure with loss of consciousness, and heart failure was defined as any heart failure with pulmonary edema or severe shortness of breath with rales.

A monitored ward was defined as a coronary care unit(CCU), an intensive care unit(ICU), an operational room(OR), an emergency room(ER), an intermediate care unit(IMCU) or a catheterization laboratory(Cath lab). A non-monitored ward was defined as a regular ward (RW). All other wards were defined as other ward, e.g. outpatient lab, radiology department, etc.

Statistical analyses

Patient characteristics are reported in means and medians, along with standard deviations and interquartile ranges, respectively. The Kaplan-Meier estimator was used for defining survival distributions; the log rank test was used to test for differences in survival. Trends in rates of COVID-19 were assessed on a monthly basis during the entire study basis.

Logistic regression was used to calculate odds ratios for 30-days survival. These models assessed the association between COVID-19 status and 30-days survival, while adjusting for age, sex and initial rhythm (shockable or non-shockable). Subgroup analyzes were done for

men, women, age ≥70 years, age <70 years, heart failure, kidney failure, diabetes, myocardial infarction and cancer.

In order to obtain estimates of overall survival, we used Cox proportional hazards model with hours since CA as the time scale. The proportional hazards assumption was fulfilled for all variables.

We used the MICE (Multiple Imputation By Chained Equations) algorithm to impute missing values(10, 11) (Supplementary Figure 1). The imputed data set was used to calculate odds ratios for 30-days survival in the overall group, as well as in COVID+ and COVID– cases. These models included age, sex, initial rhythm, time to start of cardiopulmonary resuscitation (CPR), time of CA, previous MI, location (other ward vs monitored, and non-monitored ward vs monitored), heart failure, EKG monitoring, diabetes and acute MI.

Analyses were done in R (v. 4.0.3, R Foundation for Statistical Computing) using RStudio.

Patient and Public Involvement statement:

No patients were involved.

Results

A total of 2,227 patients were enrolled in the SRCR between 01/01/2020 and 31/12/2020.

After excluding patients <18 years (n=68) and pre-pandemic cases (n=546), 1,613 cases

remained from 15/03/2020 to 31/12/2020 and constituted the final study population (Supplementary Figure 2). There was a high rate of information on COVID-19 status during the study period among patients registered in the registry (Supplementary Figure 3).

Baseline characteristics

The overall mean age was 70.8 years, and the proportion of women was 37.6%. At the end of follow-up, 341 (32.7%) patients were alive. The mean age was similar in the three groups: 70.9 years among COVID+, 71.0 years among COVID– cases, and 70.2 years in cases with UNA (Supplementary Figure 4). The proportion of women was also similar; 37.6% in COVID+, 36.6% in COVID– and 41.0% in UNA cases.

A regular ward (RW) was the most common place of cardiac arrest in all 3 groups with rates of 45.1% among COVID+, 44.1% among COVID– and 31.4% among UNA (Table 1). The emergency room (ER) was the second most common location for COVID+ cases (15.9%). The ER was the location of cardiac arrest in 17.6% of UNA cases and 13.1% for COVID– cases.

Regarding comorbidities, acute myocardial infarction was observed in 12.0% of COVID+ and 23.6% of COVID– cases. Previous myocardial infarction was observed in 11.7% of COVID+,

20.8% of COVID– and 11.7% of UNA cases. The prevalence of heart failure, cancer and diabetes was similar across all groups (Table 1).

Fewer cases among COVID+ individuals had a shockable rhythm (17.3%), compared with COVID– (24.9%) and UNA (27.0%). Likewise, fewer cases among COVID+ (22.7%) were defibrillated, compared with COVID– (31.5%) and UNA (32.8%). COVID+ cases were ventilated in 54.8% of cases before rescue team arrival, as compared with 63.2% and 69.2% in COVID– and UNA, respectively.

Follow-up and

Return of spontaneous circulation (ROSC) after initial resuscitation, was less common in COVID+ cases, as compared with COVID– and UNA. Also, angiography, PCI, pacemaker and ICD implantation post cardiac arrest were less common in COVID+ cases.

Sex specific characteristics

Acute myocardial infarction was observed in 21.2% of COVID+ women and 7.6% of COVID+ men. Previous myocardial infarction was observed in 4.7% of COVID+ women and 16.2% of COVID+ men. The prevalence of previous stroke, renal failure, heart failure, cancer and diabetes were similar among men and women, as was location at the time of cardiac

arrest. COVID+ men were more likely to have a shockable rhythm (20.8%) compared with COVID+ women (11.5%) and to be defibrillated (26.4% in men vs 16.9% in women) (Supplemntary Table 1).

Monthly trends in COVID-19 associated IHCA

In March, April and May 14%, 23% and 20% of patients suffering IHCA were COVID+ (data from 16th March). The proportion of COVID+ cases diminished rapidly during June to July. From September onwards the COVID+ cases increased again to reach 19% in December. In Figure 1A additional details regarding monthly variations are presented.

Etiology of IHCA

The most common cause of IHCA among COVID+ was respiratory insufficiency (24%, n=24). The second most common cause was sepsis or other infection (19%, n=19) among COVID+. Respiratory insufficiency and sepsis/other infection were less common in the other groups (Figure 1B), which instead displayed higher rates of acute myocardial infarction.

Clinical conditions one hour prior to IHCA

As evident in Figure 1C which describes the clinical conditions preceding (up to 60 minutes) the cardiac arrest, hypoxia was more common among COVID+ (57%), as compared with

COVID– (34%). Regarding arrhythmia, heart failure, hypotension and seizure the percentages were more similar.

Survival analysis

The Kaplan Meier plots (Figure 2) show that COVID+ cases generally had a lower probability of survival compared to COVID– and UNA cases. The overall 30-day survival (Figure 2A) was 21% among COVID+, compared with 36% in COVID– cases (p=0.00086). The subgroup analysis of women (Figure 2B) showed low survival rates in COVID+ cases (16% 30-day survival). The subgroup analysis of men (Figure 2C) showed low survival rates in COVID+ cases (23% 30-day survival) but not as low as the women. Regarding age, 30 days survival among COVID+ aged >70 years was 18% (Figure 2D), as compared with 25% of COVID+ cases aged 70 or younger (Figure 2E). Survival curves for the subgroups of individuals with cancer, heart failure and diabetes, did not display any clear patterns (Figure 2F-2H). All p values were >0.1. Patients with kidney failure had a 30 days survival of 13% among COVID+ cases (Figure 2I). Patients with acute MI had a 30 days survival of 8% among COVID+ cases (Figure 2J).

Cox adjusted survival curves are presented in Supplementary Figure 5; COVID+ cases displayed the lowest probability of survival, whereas there was no material difference between COVID– and UNA cases.

Odds ratios for 30-days survival

When adjusted for age, sex and initial rhythm the odds ratio for 30-day survival, comparing COVID+ vs. COVID–, were 0.50 (0.33-0.76) overall, 0.53 (0.31-0.88) for men, and 0.44 (0.20-0.88) for women. In the subgroup of patients with heart failure, myocardial infarction and cancer, we found no statistically significant associations, whereas in the subgroup of COVID+ patients with kidney failure, odds ratio for 30-days survival was 0.43 (0.16–0.99), when compared with COVID– (Figure 3).

Predictors of survival

Regarding predictors for 30-days survival among COVID+ we note that confidence intervals were generally wide. Lack of ECG monitoring and later start of CPR showed point estimates below 1.0, although non-significant. Odds ratio for patients treated in non-monitored wards was 0.26 (95% CI 0.08-0.78) as compared with monitored ward(Figure 4). No coexisting condition was associated with survival among COVID+ cases.

Regarding COVID– cases the factors that were significantly associated with 30-days survival were shockable rhythm (OR 4.18 [95% CI 2.69–6.02]), ECG monitoring (2.67 [95% CI 1.82–3.95]), heart failure (OR 0.58 [95% CI 0.40–0.83]) and diabetes (OR 0.64 [95% CI 0.44–0.92]) were significantly associated with death(Figure 4).

Discussion

This study elucidates characteristics and outcomes in patients with COVID-19 who develop IHCA. As of writing this report the pandemic is still surging worldwide with hundreds of thousands of new cases every day, despite successful vaccinations efforts. We show that the prevalence of COVID-19 among patients suffering an IHCA increased to approximately one in four cardiac arrests during the first pandemic wave, and one in five cardiac arrests during the second wave. Non-respiratory and non-infectious causes are dominating the cause of

cardiac arrest in COVID+ patients, and probability of survival at 30-days is halved by the presence of COVID-19.

Regarding location of CA, we note that the most common location for COVID-19 patients was regular wards, which are not monitored. This is unfortunate since our analyses showed that type of ward (monitored vs non-monitored) was significantly associated with survival, such that COVID+ cases in non-monitored wards displayed 74% lower probability of survival as compared with COVID+ cases in monitored wards. As compared with COVID– cases, cardiac arrest in the ER was more common in COVID+ cases. The often rapid deterioration of cardiopulmonary function in patients with COVID-19 may be one of the explanations for this finding. Fewer COVID+ cases were located in the CCU which is an expected finding due to the fact that cardiac etiology was less common among these patients.

We note that the most common cause of cardiac arrest in COVID+ cases, as well as the most frequent clinical condition directly preceding the arrest, is respiratory. The high rate of respiratory etiology was driven by men (Supplementary Figure 6-7). A total of 57% of cases displayed hypoxia before cardiac arrest. This may highlight an opportunity for improving outcomes; measures to prevent hypoxia and to correct it immediately may reduce the risk of cardiac arrest in patients with COVID-19. On the other hand, it can be argued that we cannot

do that inference because we have not studied patients with and without hypoxia and followed them in terms of risk of developing cardiac arrest (all our cases had already developed cardiac arrest). However, we know that COVID-19 causes ARDS (acute respiratory distress syndrome) and hypoxia, which can induce cardiac arrest.

However, the fact that 43% of cases with COVID-19 did not have hypoxia prior to cardiac arrest suggests that other factors are important as well. Thromboembolism, myocardial infarction, arrhythmias, etc. may all contribute to the development of a cardiac arrest(12).

A previous study from Wuhan showed that 87.5% of COVID+ cases with IHCA had a respiratory etiology and a study from Southwest Georgia that 53% of the patients with IHCA and COVID-19 had ARDS(5, 7). We report much lower rates of respiratory etiology (24%), which may be due to several factors; e.g. in our study we had a total of 22 possible categories for cause of CA, as compared with two categories in the study from Wuhan. Also, patients in the study from Wuhan had severe COVID-19 and in our study population we do not know the severity of the disease.

The survival rates were poor among COVID+ patients with an overall 30-days survival of 21%, compared to 36% among COVID–. The survival rate was, however, not as low as in the

study from Wuhan, in which 3% (151 patients studied) survived, or in the study from New York with 31 patients or in the study from Southwest Georgia with 63 patients with none surviving (5, 7, 13). One reason for the poor survival could be the small number of patients found in a shockable rhythm (17% vs. 25% for COVID+ and COVID–, respectively) since patients with shockable rhythm have a more favorable outcome. After adjusting for sex, age and shockable rhythm the 30-day survival was though still significantly worse among patients with an ongoing infection.

We demonstrate that COVID+ women had halved chance of survival at 30 days, compared with COVID– women. We find it interesting that COVID+ women had acute MI three times as often as men, despite the fact that men exhibited shockable rhythm – and were defibrillated – twice as often as women; this cannot be explained by differences in prevalent heart failure, as there were none across men and women.

Strengths and limitations. This study includes all IHCAs in Sweden which were reported to SRCR. The sample recorded in the SRCR is unbiased since all hospitals participate in the registry and all hospitals report data on COVID-19 status. However, we do not know the severity of the COVID-19 infection, and we do not know if COVID-19 was the main reason for admission to hospital. With regards to the classification of COVID-19 status, we have

performed a misclassification analysis which demonstrated that odds ratios were not materially affected by misclassification bias. Our study only includes IHCAs receiving CPR. This leaves out all other patients with IHCA, e.g with a Do Not Attempt Resuscitation order.

It is important to stress the fact that our regression model that included only COVID-19 cases must be interpreted with caution due to the large number of predictors in the model, which had relatively few patients (resulting in wide confidence intervals). Further studies are warranted, using a larger study population, and a longer follow up especially regarding subgroup analyses, neurological outcomes and the quality of life for the patients. Information about the severity of COVID-19 and the reason for admission to the hospital would add valuable insights as well.

Conclusion

During pandemic peaks, up to one fourth of all IHCAs are complicated by COVID-19, and these patients have halved chance of survival, with women displaying the worst outcomes. The Pandemic has changed the whole world and the halved chance of survival displays just a little part of how it has affected us all.

Funding

This work was supported by the Swedish Research Council [2019-02019] and the Swedish Heart and Lung Foundation [20200261].

Conflict of interest: none declared.

Author Statement: Astrid Holm and Araz Rawshani designed the study. Astrid Holm wrote the first draft of the manuscript, analyzed all data and made initial interpretations of data. Araz Rawshani has been supervising. Matilda Jerkeman, Pedram Sultanian, Peter Lundgren, Annica Ravn-Fischer, Johan Israelsson, Jasna Giesecke and Johan Herlitz revised the article critically for important intellectual content and approved the version of the article to be published.

Ethics statement: The study was approved by the Swedish Ethical Review Authority (ID 2020-02017). The data was anonymized before the authors accessed it for the purpose of the study.

Data sharing plan: No additional data available

References

1. Johns Hopkins University and Medicine COVID-19 map [internet] Johns Hopkins Coronavirus Resource Centre 2020. [updated 13/5/2021. Available from: https://coronavirus.jhu.edu/map.html.
2. Dödsorsaker första halvåret 2020. Socialstyrelsen. Hälso- och sjukvård.; 2020 17/11/2020.
3. Andersson J. Samhällsspridning av coronaviruset i Sverige. Läkartidningen. 16/03/2020.
4. Hayek SS, Brenner SK, Azam TU, Shadid HR, Anderson E, Berlin H, et al. In-hospital cardiac arrest in critically ill patients with covid-19: multicenter cohort study. Bmj. 2020;371:m3513.
5. Shah P, Smith H, Olarewaju A, Jani Y, Cobb A, Owens J, et al. Is Cardiopulmonary Resuscitation Futile in Coronavirus Disease 2019 Patients Experiencing In-Hospital Cardiac Arrest? Crit Care Med. 2021;49(2):201-8.
6. Mitchell OJL, Yuriditsky E, Johnson NJ, Doran O, Buckler DG, Neefe S, et al. In-hospital cardiac arrest in patients with coronavirus 2019. Resuscitation. 2021;160:72-8.
7. Shao F, Xu S, Ma X, Xu Z, Lyu J, Ng M, et al. In-hospital cardiac arrest outcomes among patients with COVID-19 pneumonia in Wuhan, China. Resuscitation. 2020;151:18-23.
8. Sultanian P, Lundgren P, Strömsöe A, Aune S, Bergström G, Hagberg E, et al. Cardiac arrest in COVID-19: characteristics and outcomes of in- and out-of-hospital cardiac arrest. A report from the Swedish Registry for Cardiopulmonary Resuscitation. European Heart Journal. 2021.
9. Hessulf F, Herlitz J, Rawshani A, Aune S, Israelsson J, Södersved-Källestedt ML, et al. Adherence to guidelines is associated with improved survival following in-hospital cardiac arrest. Resuscitation. 2020;155:13-21.
10. Stef van Buuren, Groothuis-Oudshoorn K. mice: Multivariate Imputation by Chained Equations in R. Journal of Statistical Software. 2011;Vol 45.
11. 3.5 Classification and regression trees [Available from: https://stefvanbuuren.name/fimd/sec-cart.html.
12. Gupta A, Madhavan MV, Sehgal K, Nair N, Mahajan S, Sehrawat TS, et al. Extrapulmonary manifestations of COVID-19. Nat Med. 2020;26(7):1017-32.
13. Sheth V, Chishti I, Rothman A, Redlener M, Liang J, Pan D, et al. Outcomes of in-hospital cardiac arrest in patients with COVID-19 in New York City. Resuscitation. 2020;155:3-5.

Table 1 Characteristics of 1613 patients with IHCA during the COVID-19 pandemic.

Variables	No infection COVID –	Ongoing infection COVID +	Unknown/NA UNA	SMD
n	1062	182	369	
**Demographics:**				
Age - mean (SD)	71.00 (13.32)	70.93 (12.43)	70.22 (13.60)	0.039
Woman - n (%)	388 (36.6)	68 (37.6)	151 (41.0)	0.061
**Location of cardiac arrest - n (%)**				0.527
Coronary care unit - n (%)	155 (14.6)	14 (7.7)	50 (13.6)	
Intensive care unit - n (%)	77 (7.3)	25 (13.7)	19 (5.1)	
Operational room - n (%)	22 (2.1)	0 (0.0)	12 (3.3)	
Emergency room - n (%)	139 (13.1)	29 (15.9)	65 (17.6)	
Outpatient lab, radiology - n (%)	49 (4.6)	7 (3.8)	28 (7.6)	
Cathlab - n (%)	98 (9.2)	8 (4.4)	60 (16.3)	
Intermediate care unit - n (%)	25 (2.4)	15 (8.2)	10 (2.7)	
Regular ward - n (%)	468 (44.1)	82 (45.1)	116 (31.4)	
Other - n (%)	29 (2.7)	2 (1.1)	9 (2.4)	
**Critical times - median (IQR):**				
Time to alert – median (IQR)	1.00 [1.00, 1.00]	1.00 [1.00, 1.00]	1.00 [1.00, 1.00]	0.078
Time to CPR - median (IQR)	0.00 [0.00, 1.00]	0.00 [0.00, 0.00]	0.00 [0.00, 1.00]	0.109
Time to defibrillation - median (IQR)	2.00 [1.00, 5.00]	2.00 [1.00, 4.75]	1.00 [1.00, 4.00]	0.141
**Comorbidities - n (%):**				
MI, ongoing - n (%)	178 (23.6)	12 (12.0)	37 (29.4)	0.292
MI, previous - n (%)	163 (20.8)	13 (11.7)	26 (18.4)	0.165
Stroke, ongoing - n (%)	30 (3.8)	4 (3.7)	4 (3.0)	0.030
Stroke, previous - n (%)	82 (10.3)	7 (6.1)	15 (10.5)	0.105
Cancer, any - n (%)	165 (20.9)	20 (17.7)	28 (20.6)	0.054
Diabetes - n (%)	224 (27.9)	36 (31.0)	38 (27.0)	0.060
Heart failure - n (%)	229 (29.7)	36 (33.0)	36 (27.9)	0.074
Ejection fraction (%) - mean (SD)	46.14 (13.74)	46.44 (11.86)	44.94 (14.82)	0.073
EF <50% - n (%)	167 (46.0)	26 (48.1)	22 (46.8)	0.029
**Kidney function category - n (%)**				0.121
eGFR <30 - n (%)	165 (21.6)	22 (20.0)	26 (20.0)	
eGFR 30–59- n (%)	216 (28.3)	32 (29.1)	44 (33.8)	
eGFR 60–89 - n (%)	198 (25.9)	25 (22.7)	30 (23.1)	
eGFR ≥90 - n (%)	185 (24.2)	31 (28.2)	30 (23.1)	
No kidney failure (eGFR ≥60) - n (%)	383 (50.1)	56 (50.9)	60 (46.2)	0.063
eGFR (ml/min/m2) - mean (SD)	66.89 (49.43)	71.26 (58.96)	63.78 (40.31)	0.099
**Cause of arrest:** - n (%)				0.629
Hemorrhage - n (%)	34 (4.9)	2 (2.0)	10 (8.1)	
Myocardial infarction/ischemia- n (%)	181 (26.2)	15 (14.9)	41 (33.3)	

infarction/ischemia 181 (26.2) 15 (14.9) 4 (33.				
Other - n (%)	213 (30.8)	30 (29.7)	41 (33.3)	
Primary arrhythmia - n (%)	101 (14.6)	8 (7.9)	12 (9.8)	
Respiratory insufficiency - n (%)	73 (10.5)	24 (23.8)	7 (5.7)	
Sepsis/infection - n (%)	45 (6.5)	19 (18.8)	4 (3.3)	
Stroke/thromboembolism - n (%)	45 (6.5)	3 (3.0)	8 (6.5)	
**Early interventions - n (%):**				
Witnessed arrest - n (%)	857 (80.9)	140 (77.8)	306 (85.0)	0.124
ECG monitoring - n (%)	635 (60.5)	89 (50.0)	221 (62.1)	0.163
CPR before AGA - n (%)	845 (91.0)	146 (93.6)	268 (88.2)	0.127
Defibrillated before AGA – n (%)	159 (17.9)	18 (11.9)	53 (19.0)	0.131
Ventilated before AGA - n (%)	503 (63.2)	74 (54.8)	175 (69.2)	0.199
Shockable rhythm - n (%)	247 (24.9)	29 (17.3)	90 (27.0)	0.158
Defibrillated, any - n (%)	323 (31.5)	40 (22.7)	111 (32.8)	0.151
Intubated - n (%)	473 (47.0)	100 (57.8)	177 (53.8)	0.145
Adrenaline given - n (%)	668 (65.6)	125 (72.7)	223 (66.4)	0.102
Antiarrhythmics - n (%)	139 (14.1)	17 (10.1)	48 (15.4)	0.107
Mechanical compressions – n (%)	109 (10.8)	18 (10.4)	66 (20.0)	0.180
Active temperature control – n (%)	54 (11.3)	5 (10.4)	3 (4.4)	0.173
**Status at rescue team arrival - n (%):**				
Consciousness - n (%)	214 (23.1)	18 (11.7)	57 (19.3)	0.204
Breathing - n (%)	288 (31.2)	30 (19.5)	84 (28.7)	0.181
Pulse - n (%)	309 (33.8)	36 (23.4)	89 (30.4)	0.154
**Follow-Up data - n (%):**				
Angiography - n (%)	115 (24.2)	8 (16.7)	15 (20.8)	0.124
PCI - n (%)	87 (18.2)	4 (8.3)	16 (21.9)	0.258
Pacemaker implanted - n (%)	80 (16.7)	2 (4.2)	4 (5.6)	0.281
ICD implanted - n (%)	36 (7.5)	1 (2.1)	2 (2.8)	0.172
ROSC - n (%)	520 (49.0)	64 (35.2)	142 (38.5)	0.188
Death at 30 days - n (%)	666 (62.7)	141 (77.5)	237 (64.2)	0.218
Death overall - n (%)	703 (66.2)	141 (77.5)	241 (65.3)	0.181

SD = standard deviation; IQR = interquartile range; SMD = standardized mean difference (difference between the means for the two groups divided by their mutual standard deviation. Values below 0.1 (10%) are considered inconsequential (i.e., no significant difference between the groups)). CPR = Cardiopulmonary resuscitation, PCI = Percutaneous Coronary Intervention, **ICD = implantable cardioverter-defibrillator. ROSC = return of spontaneous circulation. AGA= alarm group arrival**

Figure Titles and Legends

Figure 1: Characteristics of IHCA according to COVID-19 status

A: Monthly proportion of COVID-19 status among patients with IHCA, stratified on COVID-19 status. In March only cases after 15/03/2020 were included.

B: Etiology of IHCA, stratified on COVID-19 status. The y-axis shows percentages for each etiology in each group.

C: Clinical conditions 1 hour prior to IHCA, stratified on COVID-19 status. Only patients with data regarding the specific condition was included.

Figure 2: Kaplan Meier survival curves

Kaplan Meier survival curves, separately for (A)Overall, (B)Women, (C)Men, (D)Age ≥70 year, (E)Age <70 year, (F)Cancer, (G)Heart failure, (H)Diabetes, (I)Kidney failure and (J)Myocardial infarction. p= log-rank p-value. The numbers under the graphs are showing the survival in percentages. Regarding myocardial infarction acute MI is presented.

Figure 3: Odds Ratio for 30-day survival

Forest plot with the adjusted odds ratio for 30-day survival among patients with ongoing infection vs. no infection and unknown/NA vs. no infection. Stratified on overall, men,

women, age < 70 years, age ≥ 70 years, heart failure, kidney failure, diabetes, myocardial infarction and cancer. Myocardial infarction was defined as acute or previous MI.

Figure 4: Odds Ratio for 30-day survival

Forest plot with odds ratio for 30-day survival, stratified on the groups, no infection, ongoing infection and overall, all in different colors. The 95% Confidence interval is shown between the bars. X-axis has a logarithmic scale. ECG=electrocardiogram, CA=cardiac arrest, MI=myocardial infarction. CI=confidence interval.

Figure 1: Characteristics of IHCA according to COVID-19 status

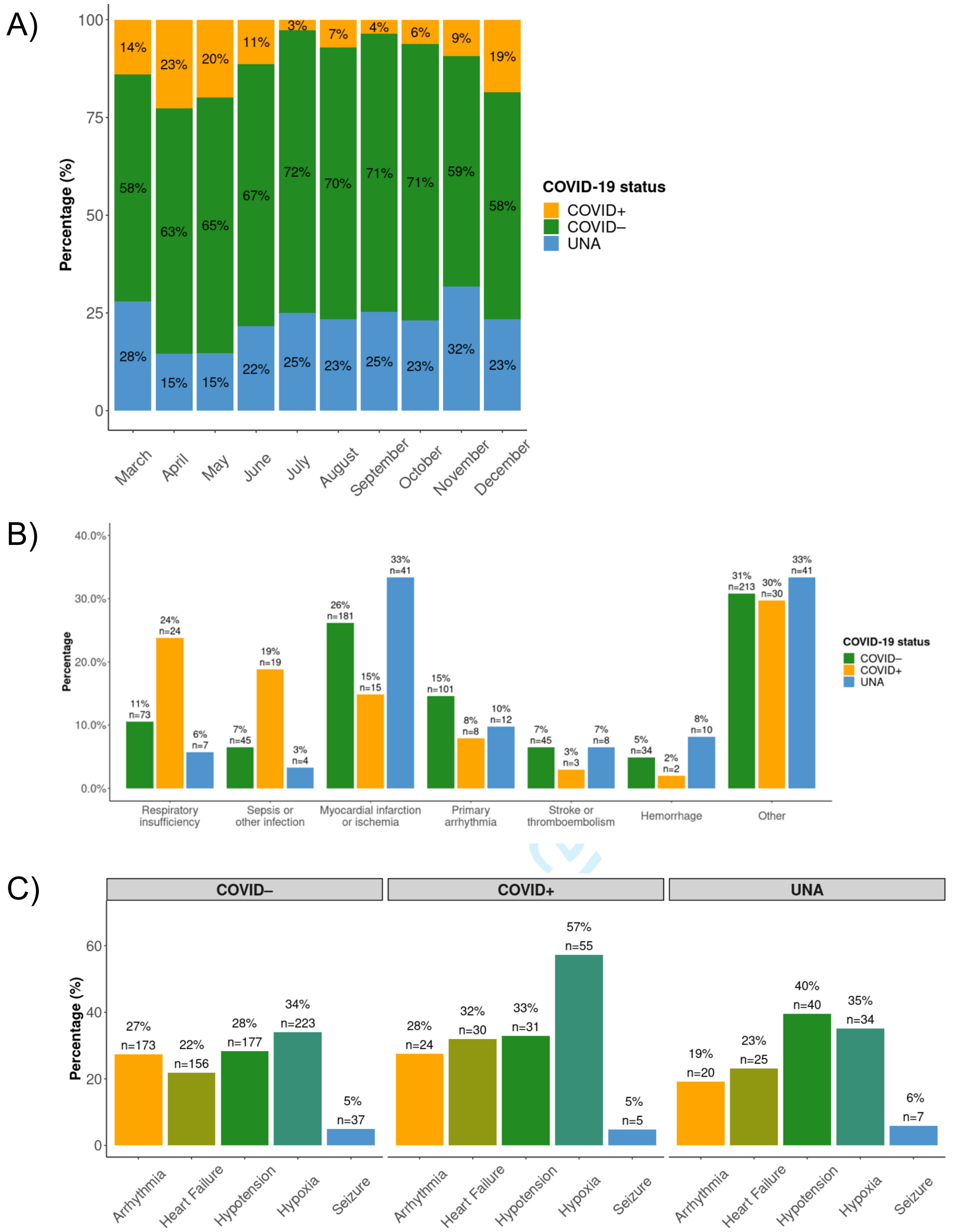

Figure 1A: Monthly proportion of COVID-19 status among patients with IHCA, stratified on COVID-19 status. In March only cases after 15/03/2020 were included.

Figure 1B: Etiology of IHCA, stratified on COVID-19 status. The y-axis shows percentages for each etiology in each group.

Figure 1C: Clinical conditions 1 hour prior to IHCA, stratified on COVID-19 status. Only patients with data regarding the specific condition was included.

Figure 2: Kaplan Meier survival curves

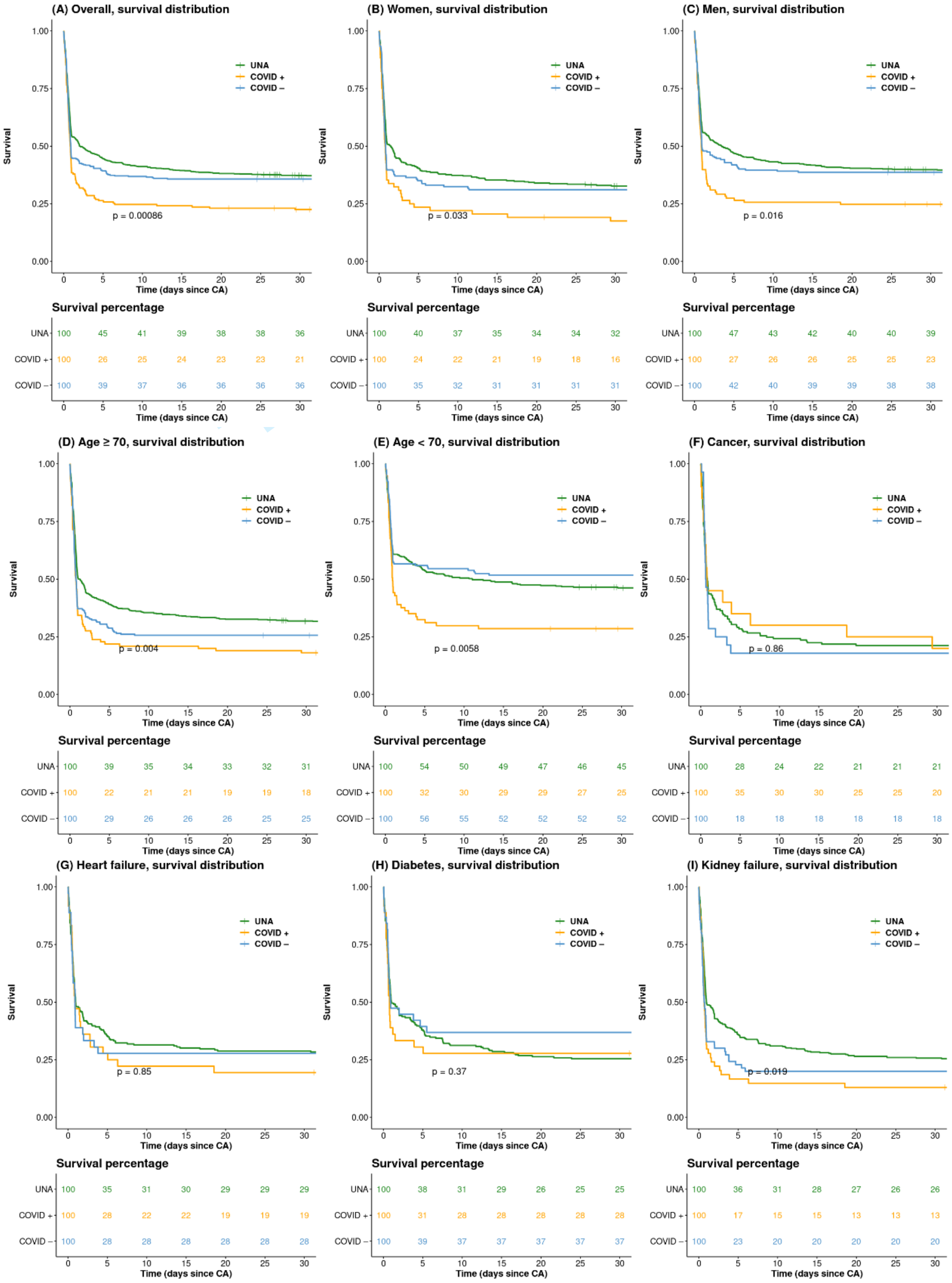

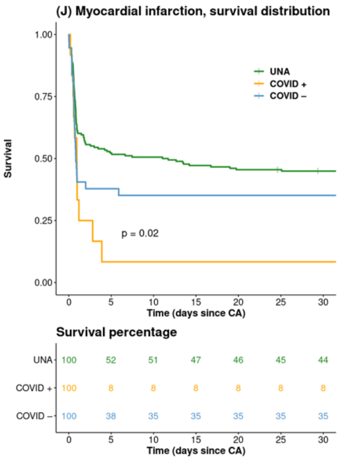

Figure 2: Kaplan Meier survival curves, separately for (A) Overall, (B) Women, (C) Men, (D) Age ≥70 year, (E) Age <70 year, (F) Cancer, (G) Heart failure, (H) Diabetes, (I) Kidney failure and (J) Myocardial infarction. p= log-rank p-value. The numbers under the graphs are showing the survival in percentages. Regarding myocardial infarction acute MI is

Figure 3: Odds Ratio for 30-day survival

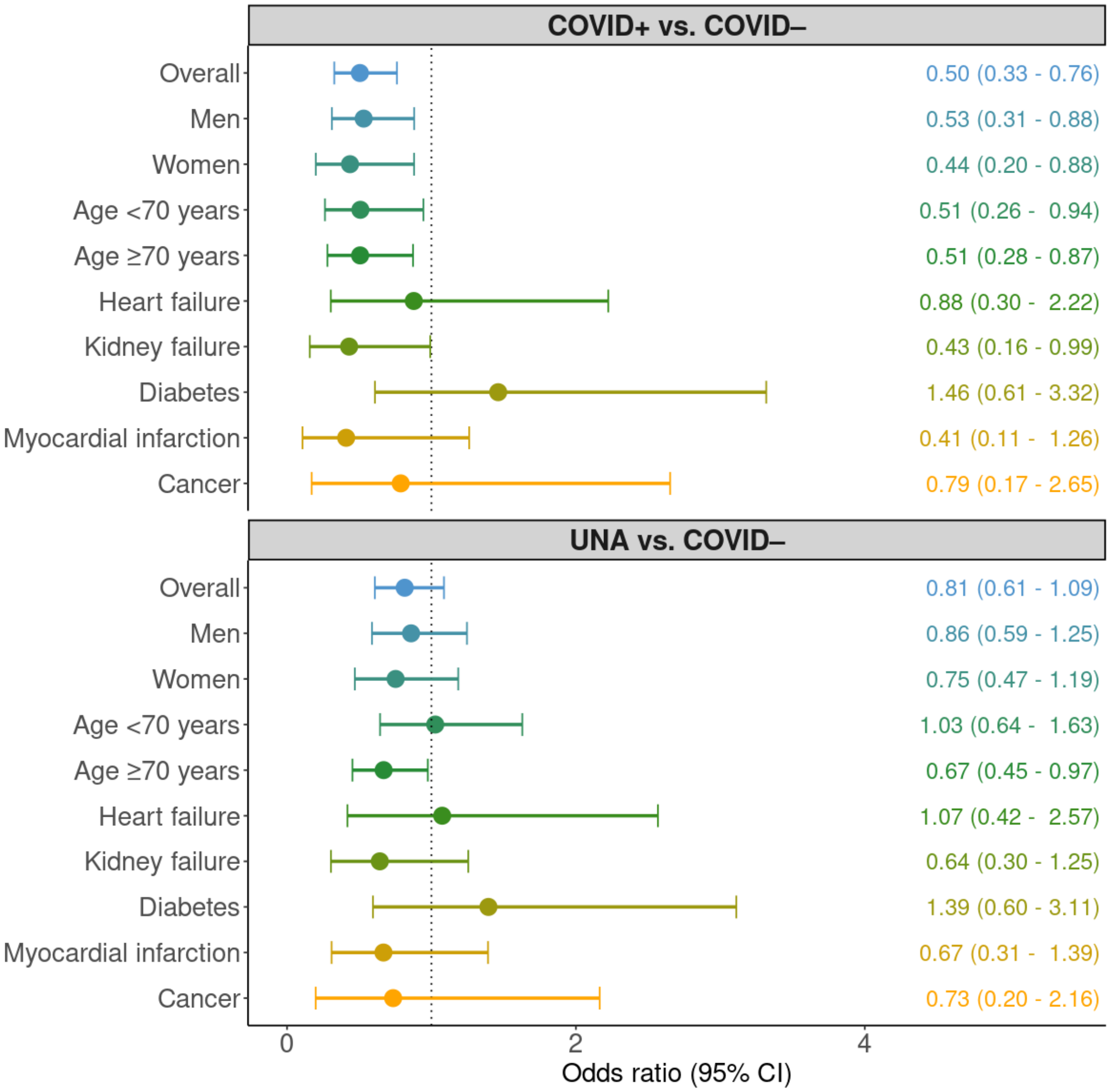

Figure 3: Forest plot with the adjusted odds ratio for 30-day survival among patients with ongoing infection vs. no infection and unknown/NA vs. no infection. Stratified on overall, men, women, age < 70 years, age ≥ 70 years, heart failure, kidney failure, diabetes, myocardial infarction and cancer. Myocardial infarction was defined as acute or previous MI.

Figure 4: Odds Ratio for 30-day survival

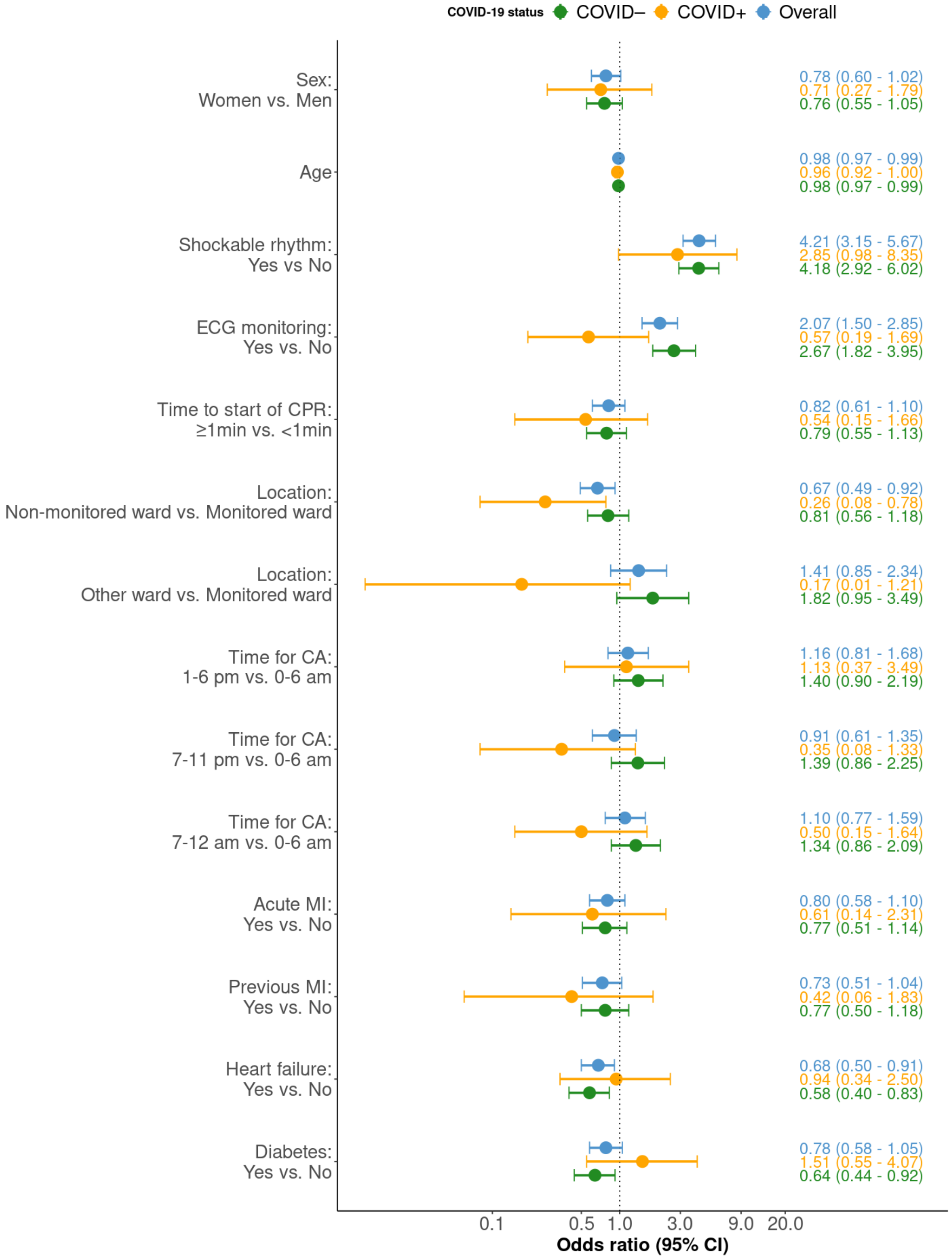

Figure 4: Forest plot with odds ratio for 30-day survival, stratified on the groups, no infection, ongoing infection and overall, all in different colors. The 95% Confidence interval is shown between the bars. X-axis has a logarithmic scale. ECG= electrocardiogram, CA= cardiac arrest, MI= myocardial infarction. CI= confidence interval.

Supplementary figures and tables

Characteristics and Outcomes in Patients with COVID-19 and In-Hospital Cardiac Arrest

For peer review only

Supplementary Table 1: Characteristics of COVID+ patients with IHCA in relation to sex.

Supplementary Table 1: Characteristics of 181 COVID+ patients with IHCA during the COVID-19 pandemic in relation to sex. One COVID+ patient had missing data on sex.

variables	Men	Women	SMD
**n**	113	68	
**Demographics:**			
Age - mean (SD)	71.39 (10.75)	70.35 (14.87)	0.080
**Location of cardiac arrest - n (%):**			0.249
Coronary care unit	7 (6.2)	7 (10.3)	
Intensive care unit	15 (13.3)	10 (14.7)	
Operational room	0 (0.0)	0 (0.0)	
Emergency room	17 (15.0)	11 (16.2)	
Outpatient lab, radiology	4 (3.5)	3 (4.4)	
Cathlab	6 (5.3)	2 (2.9)	
Intermediate care unit	11 (9.7)	4 (5.9)	
Regular ward	52 (46.0)	30 (44.1)	
Other	1 (0.9)	1 (1.5)	
**Critical times - median (IQR):**			
Time to alert – median (IQR)	1.00 [1.00, 1.00]	1.00 [1.00, 1.00]	0.256
Time to CPR - median (IQR)	0.00 [0.00, 0.00]	0.00 [0.00, 1.00]	0.031
Time to defibrillation - median	2.00 [1.00, 5.00]	2.00 [1.00, 2.00]	0.009
**Comorbidities - n (%):**			
MI, ongoing - n (%)	5 (7.6)	7 (21.2)	0.396
MI, previous - n (%)	11 (16.4)	2 (4.7)	0.391
Stroke, ongoing - n (%)	4 (5.8)	0 (0.0)	0.351
Stroke, previous - n (%)	5 (7.0)	2 (4.7)	0.102
Cancer, any - n (%)	13 (18.8)	6 (14.0)	0.132
Diabetes - n (%)	24 (33.3)	12 (27.9)	0.118
Heart failure - n (%)	23 (33.8)	13 (32.5)	0.028
Ejection fraction (%) - mean (SD)	44.84 (12.22)	49.31 (10.56)	0.392
EF <50% - n (%)	19 (51.4)	7 (43.8)	0.153
**Kidney function category - n (%):**			0.357
eGFR <30	16 (22.9)	6 (15.0)	
eGFR 30–59	17 (24.3)	15 (37.5)	
eGFR 60–89	18 (25.7)	7 (17.5)	
eGFR ≥90	19 (27.1)	12 (30.0)	
No kidney failure (eGFR ≥60)	37 (52.9)	19 (47.5)	0.107
eGFR (ml/min/m2) - mean (SD)	72.72 (65.75)	68.70 (45.34)	0.071
**Cause of arrest - n (%):**			0.920
Hemorrhage	1 (1.5)	1 (2.9)	
Myocardial infarction/ischemia	7 (10.6)	8 (23.5)	
Other	18 (27.3)	12 (35.3)	
Primary arrhythmia	3 (4.5)	5 (14.7)	
Respiratory insufficiency	17 (25.8)	7 (20.6)	

Sepsis / infection	18 (27.3)	1 (2.9)	
Stroke / thromboembolism	2 (3.0)	0 (0.0)	
**Early interventions - n (%):**			
Witnessed arrest - n (%)	86 (76.8)	53 (79.1)	0.056
ECG monitoring - n (%)	56 (50.5)	33 (50.0)	0.009
CPR before AGA - n (%)	90 (92.8)	55 (94.8)	0.085
Defibrillated before AGA - n (%)	13 (13.8)	5 (8.9)	0.155
Ventilated before AGA- n (%)	49 (56.3)	25 (53.2)	0.063
Shockable rhythm - n (%)	22 (20.8)	7 (11.5)	0.254
Defibrillated, any - n (%)	29 (26.4)	11 (16.9)	0.231
Intubated - n (%)	61 (57.0)	38 (58.5)	0.029
Adrenaline given - n (%)	76 (70.4)	48 (76.2)	0.132
Antiarrhythmics - n (%)	11 (10.4)	6 (9.7)	0.023
Mechanical compressions - n (%)	12 (10.9)	5 (8.1)	0.097
Active temperature control - n (%)	2 (6.1)	3 (20.0)	0.423
**Status at rescue team arrival - n**			
Consciousness - n (%)	11 (11.3)	6 (10.7)	0.020
Breathing - n (%)	18 (18.6)	11 (19.6)	0.028
Pulse - n (%)	22 (22.7)	13 (23.2)	0.013
**Follow-Up data - n (%):**			
Angiography - n (%)	4 (12.1)	4 (26.7)	0.374
PCI - n (%)	2 (6.1)	2 (13.3)	0.248
Pacemaker implanted - n (%)	0 (0.0)	2 (13.3)	0.555
ICD implanted - n (%)	0 (0.0)	1 (6.7)	0.378
ROSC - n (%)	40 (35.4)	24 (35.3)	0.002
Death at 30 days - n (%)	85 (75.2)	56 (82.4)	0.175
Death overall - n (%)	85 (75.2)	56 (82.4)	0.175
Discharged alive - n (%)	16 (22.2)	6 (14.0)	0.216

SD = standard deviation; IQR = interquartile range; SMD = standardized mean difference (difference between the means for the two groups divided by their mutual standard deviation. Values below 0.1 (10%) are considered inconsequential (i.e., no significant difference between the groups)). CPR = cardiopulmonary resuscitatio*n*, PCI = percutaneous coronary intervention, ICD = implantable cardioverter-defibrillator. ROSC = return of spontaneous circulation. AGA= alarm group arrival.

Supplementary Figure 1: Missing data before and after imputation with MICE

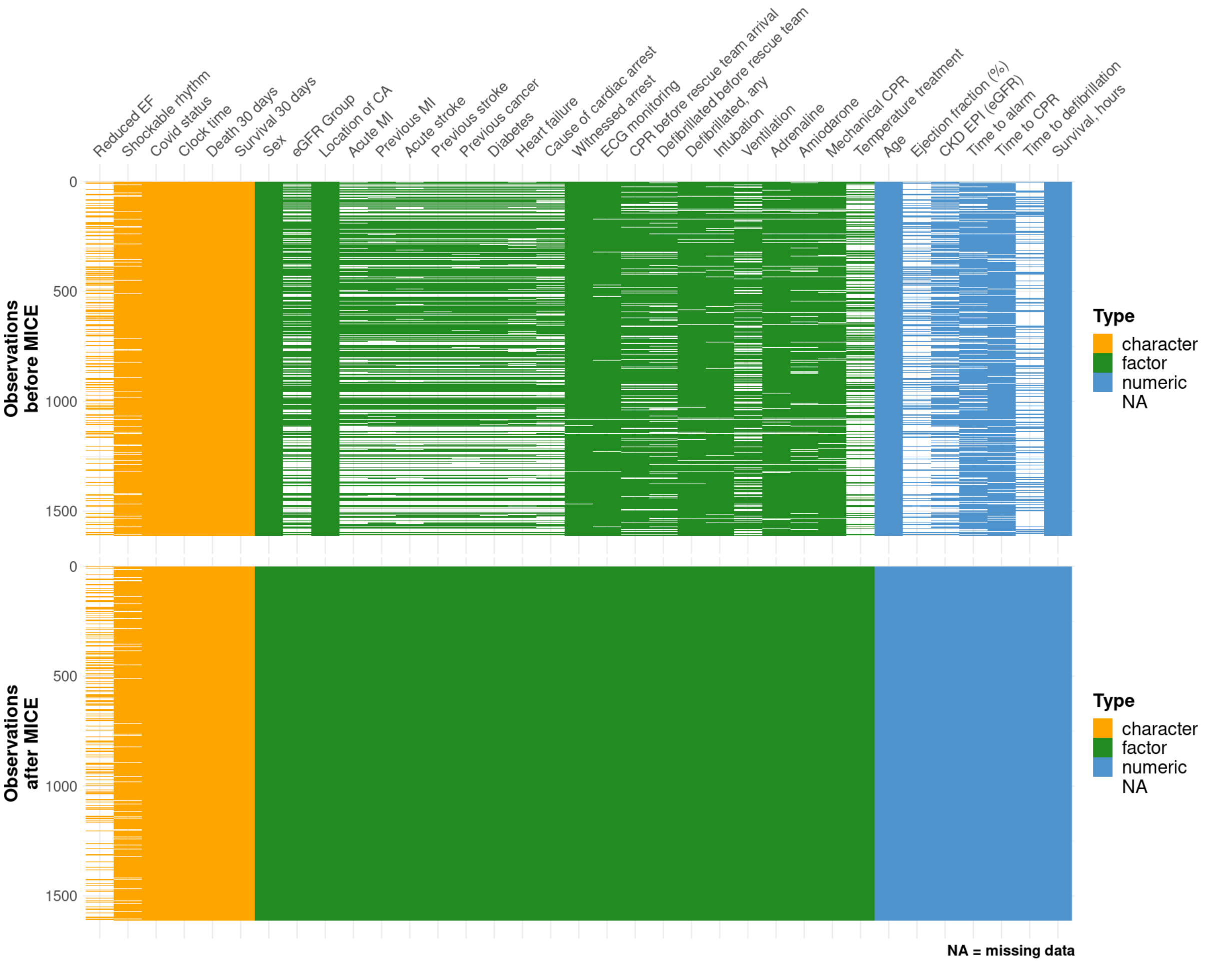

Supplementary Figure 1: Missing data before and after imputation with MICE. A graphical view of the entire dataset is printed. Each column (variable) is depicted at the top and column color depicts type of variable. Each patient represents a row and white gaps indicate a missing data entry.

For peer review only - http://bmjopen.bmj.com/site/about/guidelines.xhtml

Supplementary Figure 2: Flow chart

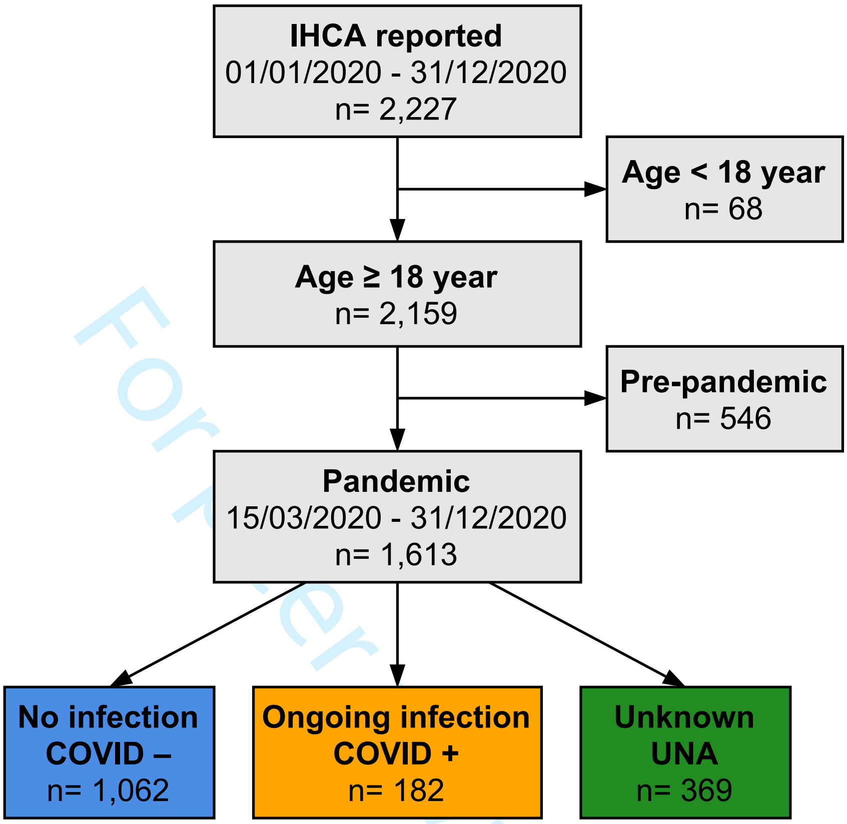

Supplementary Figure 2: Flow chart of the study population. Patients who were less than 18 year of age, and cases occurring in the pre-pandemic period were excluded.

For peer review only

Supplementary Figure 3: Information on COVID-19 status during the study period.

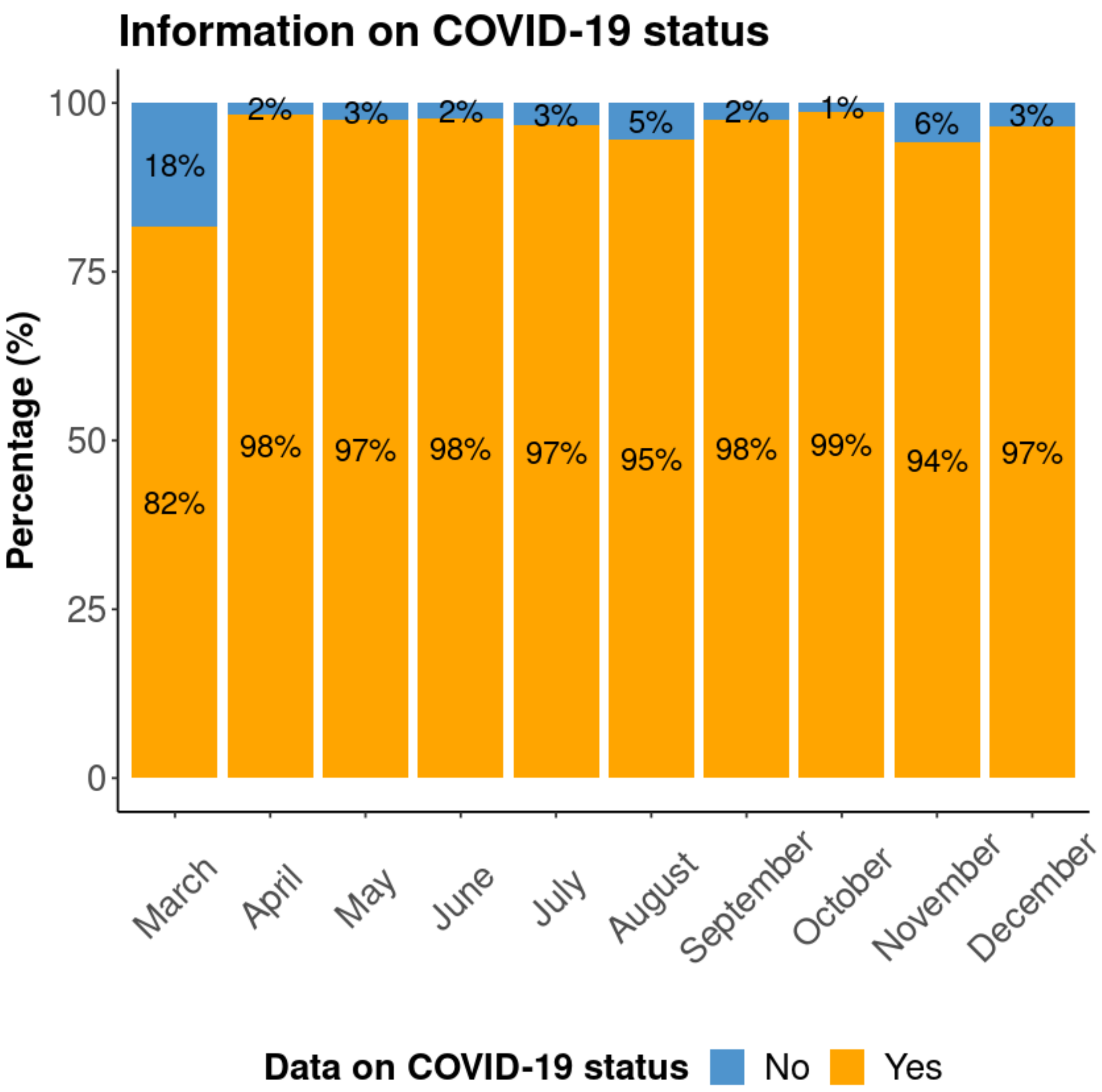

Supplementary Figure 3: Information on COVID-19 status during the study period. No equals missing data, i.e. no information on COVID-19 status available. Yes equals, COVID +, COVID – or Unknown. In March only cases after 15/03/2020 were included.

Supplementary Figure 4: Distribution of age

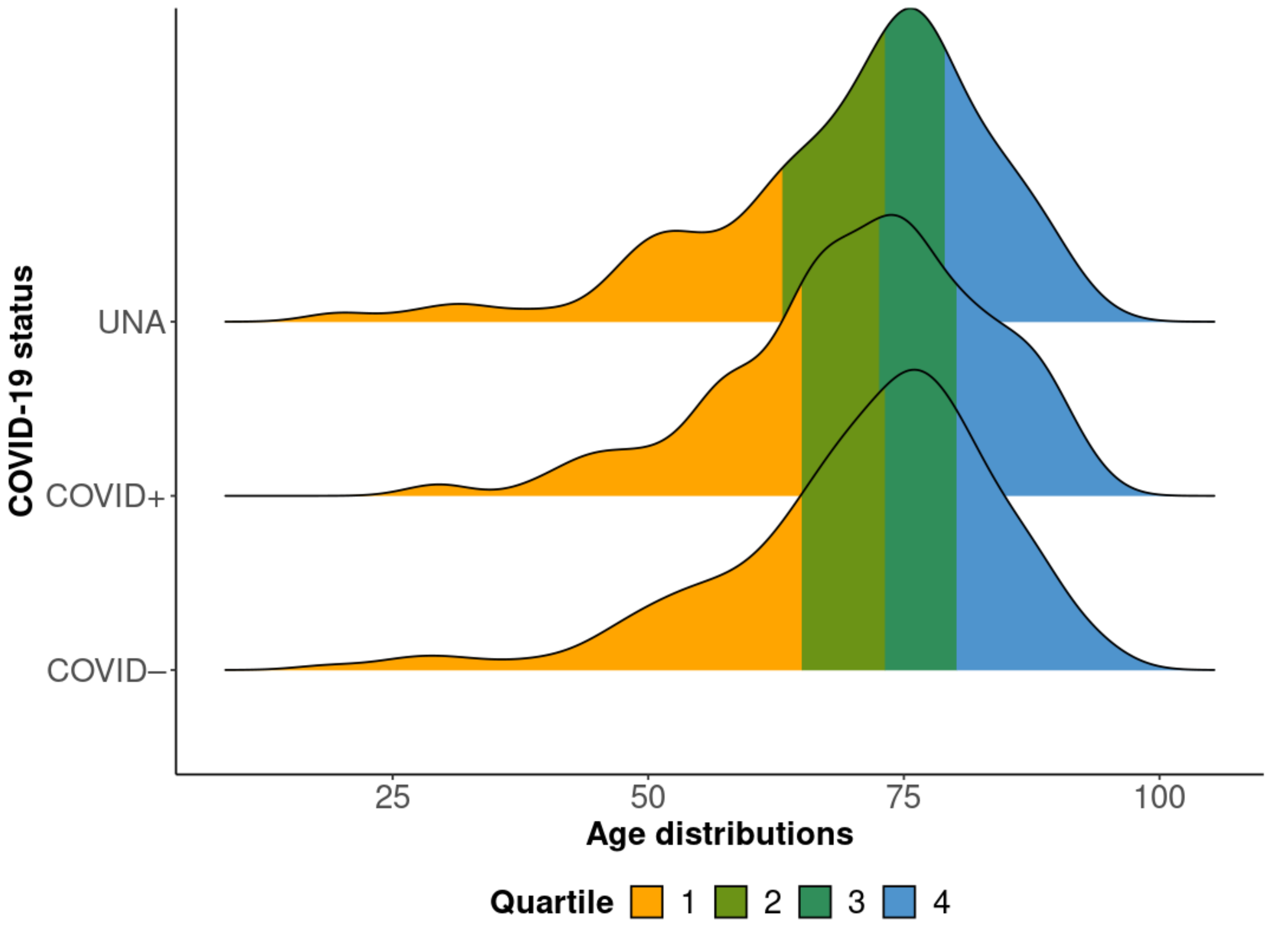

Supplementary Figure 4: Distribution of age in relation to COVID-19 status.

For peer review only - http://bmjopen.bmj.com/site/about/guidelines.xhtml

Supplementary Figure 5: Etiology of IHCA, according to sex

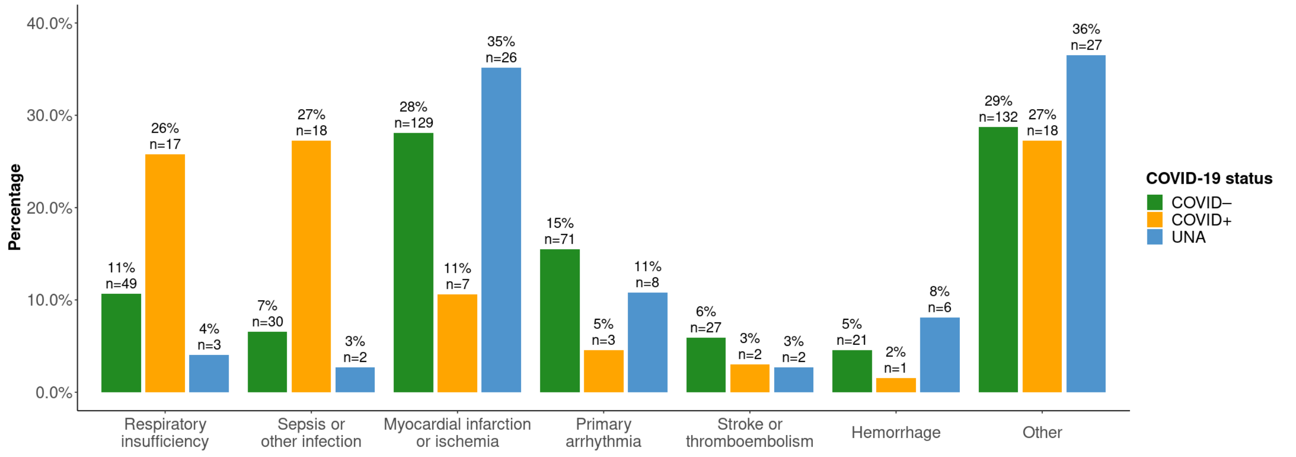

Supplementary Figure 5A: Etiology of IHCA, men only.

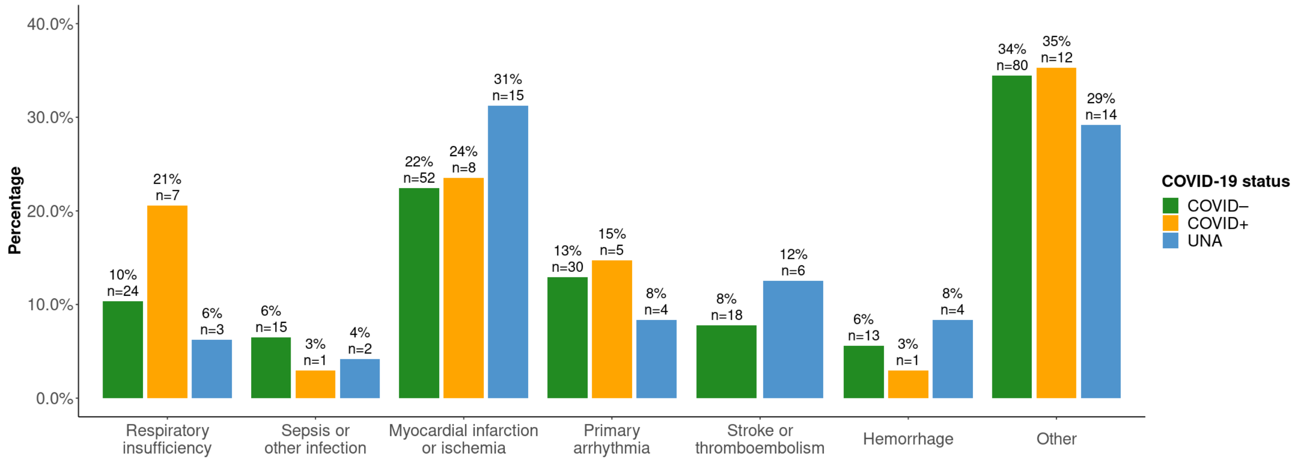

Supplementary Figure 5B: Etiology of IHCA, women only.

Supplementary Figure 6: Conditions preceding IHCA, according to sex

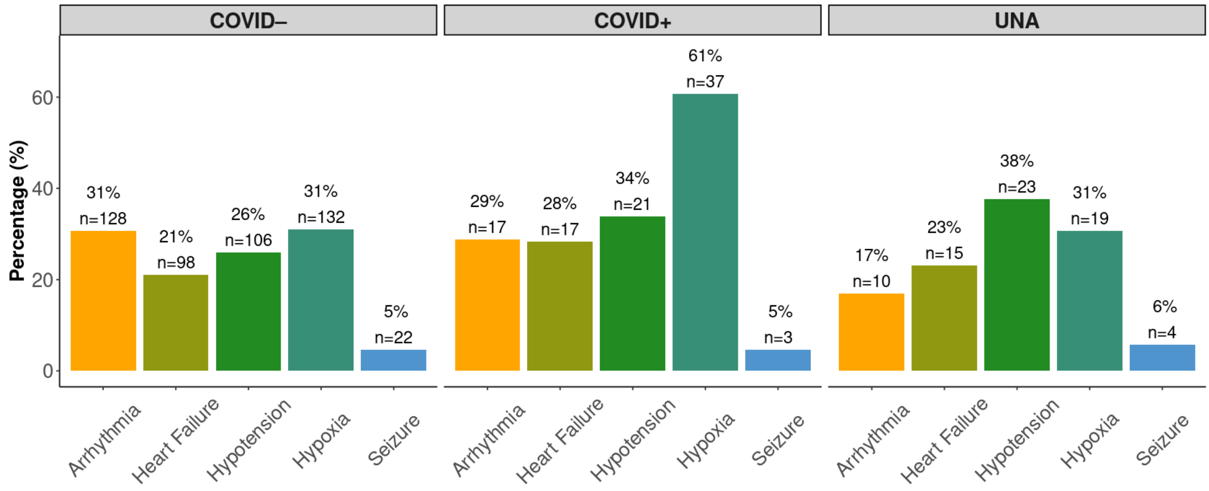

Supplementary Figure 6A: Conditions preceding IHCA, men only.

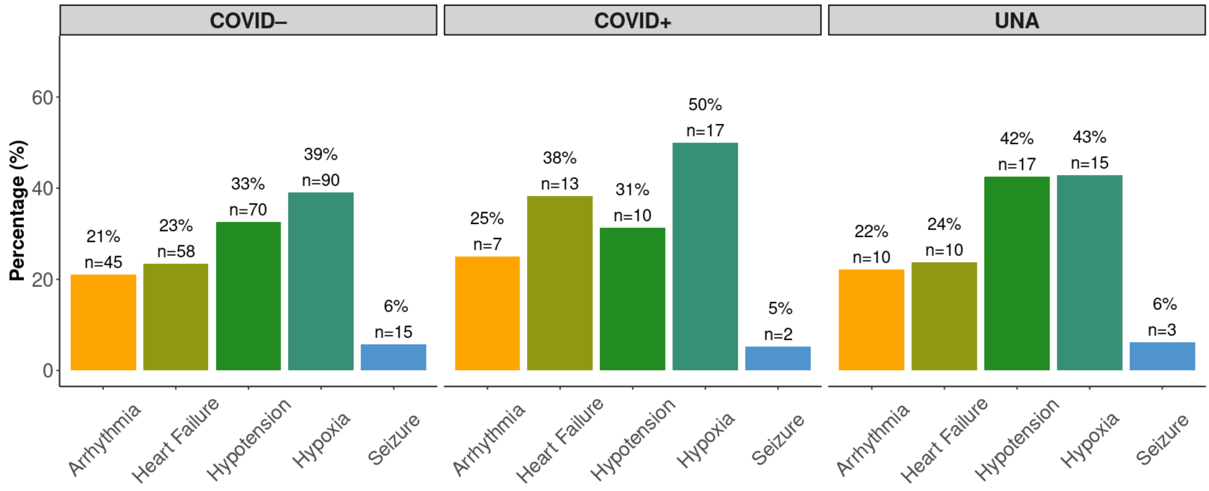

Supplementary Figure 6B: Conditions preceding IHCA, women only.

Supplementary Figure 7: Cox adjusted survival curve for the overall population

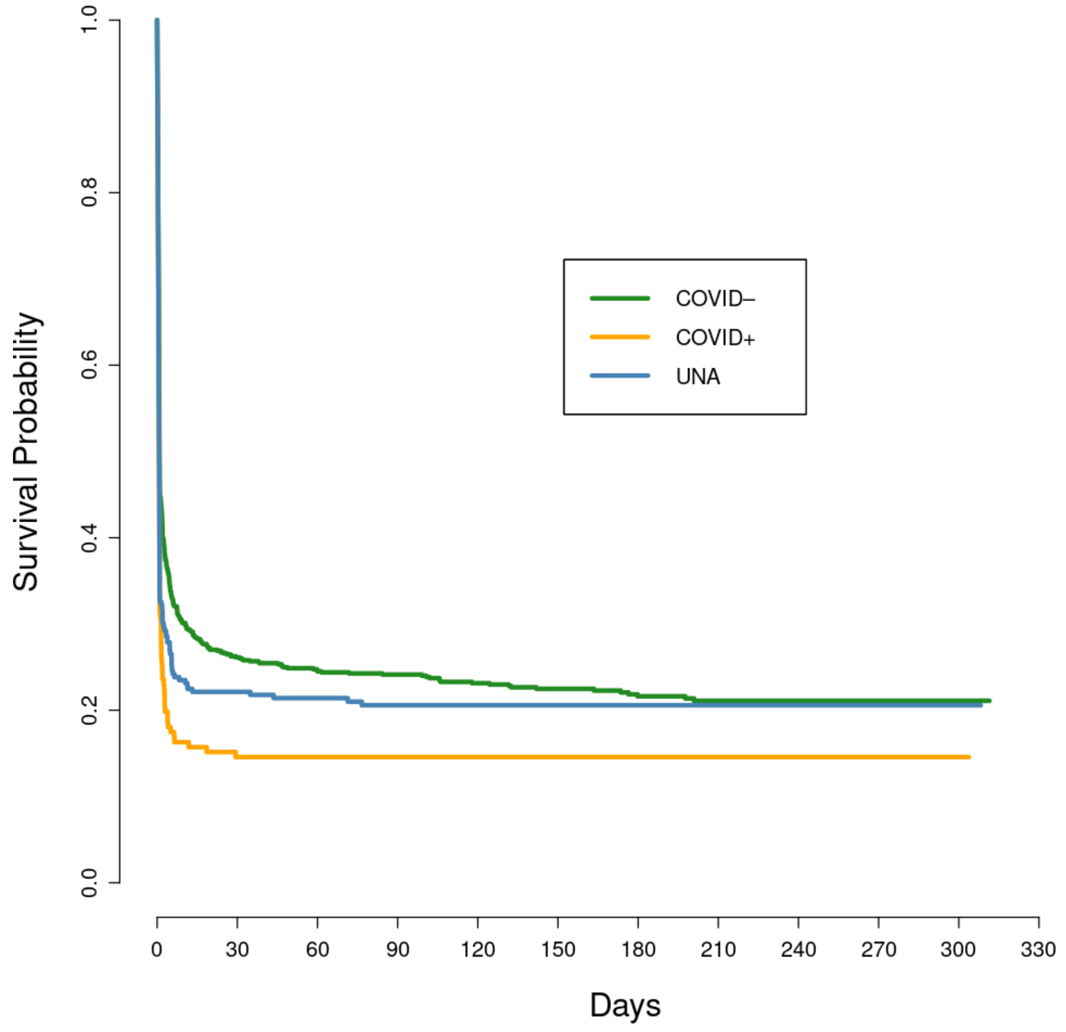

Supplementary Figure 7: Cox adjusted survival curve for the overall population, stratified on COVID-19 status.

only

STROBE Statement—checklist of items that should be included in reports of observational studies

	Item No	Recommendation	Page No
**Title and abstract**	1	(*a*) Indicate the study's design with a commonly used term in the title or the abstract	1
		(*b*) Provide in the abstract an informative and balanced summary of what was done and what was found	2-3
**Introduction**			
Background/rationale	2	Explain the scientific background and rationale for the investigation being reported	2
Objectives	3	State specific objectives, including any prespecified hypotheses	2
**Methods**			
Study design	4	Present key elements of study design early in the paper	2
Setting	5	Describe the setting, locations, and relevant dates, including periods of recruitment, exposure, follow-up, and data collection	2
Participants	6	(*a*) *Cohort study*—Give the eligibility criteria, and the sources and methods of selection of participants. Describe methods of follow-up *Case-control study*—Give the eligibility criteria, and the sources and methods of case ascertainment and control selection. Give the rationale for the choice of cases and controls *Cross-sectional study*—Give the eligibility criteria, and the sources and methods of selection of participants	2
		(*b*) *Cohort study*—For matched studies, give matching criteria and number of exposed and unexposed *Case-control study*—For matched studies, give matching criteria and the number of controls per case	2
Variables	7	Clearly define all outcomes, exposures, predictors, potential confounders, and effect modifiers. Give diagnostic criteria, if applicable	5-6
Data sources/ measurement	8*	For each variable of interest, give sources of data and details of methods of assessment (measurement). Describe comparability of assessment methods if there is more than one group	*5*
Bias	9	Describe any efforts to address potential sources of bias	3-4
Study size	10	Explain how the study size was arrived at	5
Quantitative variables	11	Explain how quantitative variables were handled in the analyses. If applicable, describe which groupings were chosen and why	5-6
Statistical methods	12	(*a*) Describe all statistical methods, including those used to control for confounding	6-7
		(*b*) Describe any methods used to examine subgroups and interactions	6-7
		(*c*) Explain how missing data were addressed	6-7
		(*d*) *Cohort study*—If applicable, explain how loss to follow-up was addressed *Case-control study*—If applicable, explain how matching of cases and controls was addressed *Cross-sectional study*—If applicable, describe analytical methods taking account of sampling strategy	6-7
		(*e*) Describe any sensitivity analyses	6-7

Continued on next page

For peer review only - http://bmjopen.bmj.com/site/about/guidelines.xhtml

**Results**			
Participants	13*	(a) Report numbers of individuals at each stage of study—eg numbers potentially eligible, examined for eligibility, confirmed eligible, included in the study, completing follow-up, and analysed	7
		(b) Give reasons for non-participation at each stage	7
		(c) Consider use of a flow diagram	Supplementary material
Descriptive data	14*	(a) Give characteristics of study participants (eg demographic, clinical, social) and information on exposures and potential confounders	7-8
		(b) Indicate number of participants with missing data for each variable of interest	7-8
		(c) *Cohort study*—Summarise follow-up time (eg, average and total amount)	7
Outcome data	15*	*Cohort study*—Report numbers of outcome events or summary measures over time	*8-9*
		Case-control study—Report numbers in each exposure category, or summary measures of exposure	
		Cross-sectional study—Report numbers of outcome events or summary measures	
Main results	16	(*a*) Give unadjusted estimates and, if applicable, confounder-adjusted estimates and their precision (eg, 95% confidence interval). Make clear which confounders were adjusted for and why they were included	7-10
		(*b*) Report category boundaries when continuous variables were categorized	
		(*c*) If relevant, consider translating estimates of relative risk into absolute risk for a meaningful time period	
Other analyses	17	Report other analyses done—eg analyses of subgroups and interactions, and sensitivity analyses	Supplementary material
**Discussion**			
Key results	18	Summarise key results with reference to study objectives	11
Limitations	19	Discuss limitations of the study, taking into account sources of potential bias or imprecision. Discuss both direction and magnitude of any potential bias	13-14
Interpretation	20	Give a cautious overall interpretation of results considering objectives, limitations, multiplicity of analyses, results from similar studies, and other relevant evidence	11-14
Generalisability	21	Discuss the generalisability (external validity) of the study results	14
**Other information**			
Funding	22	Give the source of funding and the role of the funders for the present study and, if applicable, for the original study on which the present article is based	14

*Give information separately for cases and controls in case-control studies and, if applicable, for exposed and unexposed groups in cohort and cross-sectional studies.

Note: An Explanation and Elaboration article discusses each checklist item and gives methodological background and published examples of transparent reporting. The STROBE checklist is best used in conjunction with this article (freely

For peer review only - http://bmjopen.bmj.com/site/about/guidelines.xhtml

available on the Web sites of PLoS Medicine at http://www.plosmedicine.org/, Annals of Internal Medicine at http://www.annals.org/, and Epidemiology at http://www.epidem.com/). Information on the STROBE Initiative is available at www.strobe-statement.org.

For peer review only

For peer review only - http://bmjopen.bmj.com/site/about/guidelines.xhtml

BMJ Open

A Cohort Study of the Characteristics and Outcomes in Patients with COVID-19 and In-Hospital Cardiac Arrest

Journal:	*BMJ Open*
Manuscript ID	bmjopen-2021-054943.R2
Article Type:	Original research
Date Submitted by the Author:	08-Nov-2021
Complete List of Authors:	Holm, Astrid; University of Gothenburg, Department of Molecular and Clinical Medicine<br>Jerkeman, Matilda; University of Gothenburg, Department of Molecular and Clinical Medicine<br>Sultanian, Pedram; University of Gothenburg, Department of Molecular and Clinical Medicine<br>Lundgren, Peter; University of Gothenburg, Department of Molecular and Clinical Medicine; Kalmar County Hospital, Department of Internal Medicine<br>Ravn-Fischer, Annica; University of Gothenburg, Department of Molecular and Clinical Medicine<br>Israelsson, Johan; Kalmar County Hospital, Department of Internal Medicine; Linnaeus University, Faculty of Health and Life Sciences<br>Giesecke, Jasna; Danderyd University Hospital<br>Herlitz, Johan; University of Borås, Centre for Prehospital Research<br>Rawshani, Araz; University of Gothenburg, Department of Molecular and Clinical Medicine
**Primary Subject Heading**:	Cardiovascular medicine
Secondary Subject Heading:	Cardiovascular medicine, Infectious diseases
Keywords:	COVID-19, CARDIOLOGY, Adult cardiology < CARDIOLOGY, Coronary heart disease < CARDIOLOGY

SCHOLARONE™
Manuscripts

BMJ

I, the Submitting Author has the right to grant and does grant on behalf of all authors of the Work (as defined in the below author licence), an exclusive licence and/or a non-exclusive licence for contributions from authors who are: i) UK Crown employees; ii) where BMJ has agreed a CC-BY licence shall apply, and/or iii) in accordance with the terms applicable for US Federal Government officers or employees acting as part of their official duties; on a worldwide, perpetual, irrevocable, royalty-free basis to BMJ Publishing Group Ltd ("BMJ") its licensees and where the relevant Journal is co-owned by BMJ to the co-owners of the Journal, to publish the Work in this journal and any other BMJ products and to exploit all rights, as set out in our licence.

The Submitting Author accepts and understands that any supply made under these terms is made by BMJ to the Submitting Author unless you are acting as an employee on behalf of your employer or a postgraduate student of an affiliated institution which is paying any applicable article publishing charge ("APC") for Open Access articles. Where the Submitting Author wishes to make the Work available on an Open Access basis (and intends to pay the relevant APC), the terms of reuse of such Open Access shall be governed by a Creative Commons licence – details of these licences and which Creative Commons licence will apply to this Work are set out in our licence referred to above.

Other than as permitted in any relevant BMJ Author's Self Archiving Policies, I confirm this Work has not been accepted for publication elsewhere, is not being considered for publication elsewhere and does not duplicate material already published. I confirm all authors consent to publication of this Work and authorise the granting of this licence.

For peer review only

A Cohort Study of the Characteristics and Outcomes in Patients with COVID-19 and In-Hospital Cardiac Arrest

Astrid Holm, MD[1], Matilda Jerkeman, MD[1], Pedram Sultanian, MD[1], Peter Lundgren, MD, PhD[1,3,7], Annica Ravn-Fischer, MD, PhD, Docent[1], Johan Israelsson, PhD[3,4], Jasna Giesecke, RN[5], Johan Herlitz, MD, PhD[2,6], Araz Rawshani, MD, PhD[1,2]

Affiliations:

[1]Department of Molecular and Clinical Medicine, Institute of Medicine, Sahlgrenska Academy, University of Gothenburg, Sweden

[2]The Swedish Registry for Cardiopulmonary Resuscitation, Centre of Registries, Västra Götaland County, Sweden

[3]Department of Internal Medicine, Division of Cardiology, Kalmar County Hospital, Region Kalmar County, Sweden

[4]Faculty of Health and Life Sciences, Linnaeus University, Kalmar, Sweden

[5]RN, Lead CPR coordinator, Clinicum- Centre for clinical skills, interprofessional education and advanced medical simulation, Danderyd University Hospital, Stockholm, Sweden

[6]Prehospen – Centre for Prehospital Research, University of Borås, Borås, Sweden

[7]Region Västra Götaland, Sahlgrenska University Hospital, Department of Cardiology, Gothenburg, Sweden

Contact information:

Astrid Holm

Email: astrid.holm@gu.se

Phone: +358 400707722

Manuscript word count: 3052

Abstract

Objective: We studied characteristics, survival, causes of cardiac arrest, conditions preceding cardiac arrest, predictors of survival, and trends in the prevalence of COVID-19 among in-hospital cardiac arrest (IHCA) cases.

Design and setting: Registry-based observational study.

Participants: We studied all cases (≥18 years of age) of IHCA receiving cardiopulmonary resuscitation (CPR) in the Swedish Registry for Cardiopulmonary Resuscitation during 15/03/2020 to 31/12/2020. A total of 1613 patients were included and divided into the following groups: ongoing infection (**COVID+;** n=182), no infection (**COVID–;** n=1062) and unknown/not assessed (**UNA**; n=369).

Main outcomes and measures: We studied monthly trends in proportions of COVID-19 associated IHCAs, causes of IHCA in relation to COVID-19 status, clinical conditions preceding the cardiac arrest and predictors of survival.

Results: The rate of COVID+ patients suffering an IHCA increased to 23% during the first pandemic wave (April), then abated to 3% in July, and then increased to 19% during the second wave (December). Among COVID+ cases, 43% had respiratory insufficiency or

infection as the underlying cause of the cardiac arrest, compared to 18% among COVID– cases. The most common clinical sign preceding cardiac arrest was hypoxia (57%) among COVID+ cases. Odds ratio for 30-day survival for COVID+ cases was 0.50 (95% CI 0.33-0.76), compared with COVID– cases.

Conclusion: During pandemic peaks, up to one fourth of all IHCAs are complicated by COVID-19, and these patients have halved chance of survival, with women displaying the worst outcomes.

Article Summary

Strengths and limitations of this study

- This study includes all IHCAs in Sweden reported to the Swedish Registry for Cardiopulmonary Resuscitation. All hospitals throughout Sweden report IHCA cases to the registry.
- This study has detailed data regarding cardiac arrest parameters, including circumstances before arrest, resuscitation efforts, post-resuscitation care and survival. The study only includes cases in whom CPR attempts were deemed clinically justified.

- Despite the nationwide coverage of the registry, we identified only 182 COVID positive patients, and a large number of patients had unknown COVID status.

Introduction

The COVID-19 pandemic has, as of Nov 6st 2021, infected over 249 million individuals and lead to the death of over 5 million individuals (1). COVID-19 is now the third leading cause of death in Sweden (2, 3). Multiple studies have demonstrated that in-hospital cardiac arrest (IHCA) among patients with COVID-19 is associated with poor survival (4-7). A recent study demonstrated that hypoxia was the main cause of cardiac arrest among 40% of patients with COVID-19 and IHCA (6).

We have previously reported on COVID-19 and IHCA in the Swedish Registry for Cardiopulmonary Resuscitation (SRCR), showing a 2.3-fold increase in 30-day mortality among cases with COVID-19, compared to pre-pandemic cases. This was mainly driven by a 9-fold increase in mortality among women with COVID-19. At the time, no case of IHCA with COVID-19 had been discharged alive (8). The current study expands our previous investigation, including more patients, longer follow-up and emphasizes on the causes of cardiac arrest, predictors of survival, coexisting conditions, and trends in the prevalence of COVID-19 among IHCA cases.

Methods

Data sources

This study is a registry-based observational study with data obtained from the SRCR during the time period 15/03/2020 to 31/12/2020. The SRCR is a national quality registry and has included IHCA cases since 2005. The data is collected by trained nurses who report patient data using a web-based protocol. The registry has previously been described in detail (9). Vital status was obtained from the Swedish Population Registry and the last day of follow up was 31/12/2020.

Study population

The study population included all patients ≥18 years of age suffering IHCA and receiving CPR throughout Sweden during the period 15/03/2020 to 31/12/2020. We used 15th of March as the start date of the pandemic as the Swedish Public Health Authority declared on March 16th 2020 that community spread had commenced (3). On 1st of April the SRCR started collecting data regarding COVID-19 status, and retrospectively identified 60 patients with COVID-19 who suffered IHCA during March (they were included in the study). Patients were divided into the following three groups: ongoing infection (COVID+; n=182), no infection (COVID–; n=1062) and unknown/not assessed (UNA; n=369). COVID+ was defined as patients registered with an ongoing COVID-19 infection, suspected ongoing infection or

patients with a recent infection (n=29). The UNA group was included in the study in order to provide a complete picture of cases enrolled in the SRCR during the time period, and to evaluate whether missingness in COVID-19 status could entail selection bias.

Variable definitions

In SRCR a patient with cardiac arrest was defined as an unconscious patient with no or abnormal breathing, in whom resuscitation or defibrillation was attempted. IHCA was defined as cardiac arrest in patients admitted to the hospital.

With regards to previous coexisting conditions, heart failure was defined as any heart failure described before cardiac arrest. Kidney failure was defined as estimated glomerular filtration rate (eGFR) below 60 ml/min/1.73 m^2, calculated using the highest creatinine before cardiac arrest with Chronic Kidney Disease Epidemiology Collaboration (CKD-EPI) formula. The SRCR records data on the highest creatinine levels analyzed up to six months prior to CA. Diabetes was defined as any diabetes diagnosis, regardless of type. Cancer was defined as any previously known cancer. Acute myocardial infarction (MI) was defined as an MI within 72 hours of CA. Previous myocardial infarction was defined as MI occurring earlier than 72 hours preceding the CA.

Regarding clinical conditions one hour prior to CA, arrhythmia was defined as any arrhythmia, hypoxia was defined as an oxygen saturation below 90%, hypotension was defined as systolic blood pressure below 90 mmHg, seizure was defined as any seizure with loss of consciousness, and heart failure was defined as any heart failure with pulmonary edema or severe shortness of breath with rales.

Wards with monitoring included the coronary care unit (CCU), intensive care unit (ICU), operating room (OR), emergency room (ER), high dependency unit (HDU) or the catheterization laboratory.

Statistical analyses

Patient characteristics are reported in means and medians, along with standard deviations and interquartile ranges, respectively. The Kaplan-Meier estimator was used for describing survival distributions; the log rank test was used to test for differences in survival. Trends in rates of COVID-19 were assessed on a monthly basis during the entire study basis.

Logistic regression was used to calculate odds ratios for 30-days survival. These models assessed the association between COVID-19 status and 30-days survival, adjusting for age, sex and initial rhythm (shockable or non-shockable). We performed subgroup analyses in relation to sex, age and coexisting conditions (heart failure, cancer, diabetes, kidney failure

and myocardial infarction). These subgroup analyses served to clarify whether the association between COVID status and survival was modified by age, sex or coexisting conditions.

In order to obtain estimates of overall survival, we used Cox proportional hazards model with hours since CA as the time scale. The proportional hazards assumption was fulfilled for all variables.

We used the MICE (Multiple Imputation By Chained Equations) algorithm to impute missing values (10, 11) (Supplementary Figure 1). The imputed data set was used to calculate odds ratios for 30-days survival in the overall group, as well as in COVID+ and COVID– cases. These models included age, sex, initial rhythm, time to start of cardiopulmonary resuscitation (CPR), time of CA, previous MI, type of ward, heart failure, ECG monitoring, diabetes and acute MI.

Analyses were done in R (v. 4.0.3, R Foundation for Statistical Computing) using RStudio.

Patient and Public Involvement statement:

No patients were involved.

Results

A total of 2,227 patients were enrolled in the SRCR between 01/01/2020 and 31/12/2020.

After excluding patients <18 years (n=68) and pre-pandemic cases (n=546), 1,613 cases

remained from 15/03/2020 to 31/12/2020 and constituted the final study population (Supplementary Figure 2). There was a high rate of information on COVID-19 status during the study period among patients registered in the registry (Supplementary Figure 3).

Baseline characteristics

The overall mean age was 70.8 years, and the proportion of women was 37.6%. At the end of follow-up, 341 (32.7%) patients were alive. The mean age was similar in the three groups: 70.9 years in COVID+, 71.0 years in COVID– cases, and 70.2 years in cases with UNA (Supplementary Figure 4). The proportion of women was also similar; 37.6% in COVID+ and 36.6% in COVID– and 41.0% in UNA cases.

A regular ward was the most common place for cardiac arrest in all 3 groups; 45.1% of COVID+, 44.1% of COVID– and 31.4% of UNA cases occurred in regular wards (Table 1). The emergency room (ER) was the second most common location for COVID+ cases (15.9%).

Regarding comorbidities, acute myocardial infarction was observed in 12.0% of COVID+ and 23.6% of COVID– cases. Previous myocardial infarction was observed in 11.7% of COVID+, 20.8% of COVID– and 11.7% of UNA cases. The prevalence of heart failure, cancer and diabetes was similar across all groups (Table 1).

Fewer cases among COVID+ individuals had a shockable rhythm (17.3%), compared with COVID– (24.9%) cases. Likewise, fewer cases among COVID+ (22.7%) were defibrillated, compared with COVID– cases (31.5%). COVID+ cases were ventilated in 54.8% of cases before rescue team arrival, as compared with 63.2% in COVID– cases.

Follow-up

Return of spontaneous circulation (ROSC) after initial resuscitation, was less common in COVID+ cases, as compared with COVID– cases. Also, angiography, PCI, pacemaker and ICD implantation post cardiac arrest were less common in COVID+ cases.

Sex specific characteristics

Acute myocardial infarction was observed in 21.2% of COVID+ women and 7.6% of COVID+ men. Previous myocardial infarction was observed in 4.7% of COVID+ women and 16.2% of COVID+ men. The prevalence of previous stroke, renal failure, heart failure, cancer and diabetes were similar among men and women, as was location at the time of cardiac arrest. COVID+ men were more likely to have a shockable rhythm (20.8%) compared with COVID+ women (11.5%), and to be defibrillated (26.4% in men vs. 16.9% in women) (Supplementary Table 1).

Monthly trends in COVID-19 associated IHCA

In March, April and May 14%, 23% and 20% of patients suffering IHCA were COVID+ (data from 16th March). The proportion of COVID+ cases diminished rapidly during June to July. From September onwards the COVID+ cases increased again to reach 19% in December. In Figure 1A additional details regarding monthly variations are presented.

Etiology of IHCA

The most common cause of IHCA among COVID+ cases was respiratory insufficiency (24%, n=24), and the second most common cause was sepsis or other infection (19%, n=19). Respiratory insufficiency and sepsis/other infection were less common in the other groups (Figure 1B), which instead displayed higher rates of acute myocardial infarction.

Clinical conditions one hour prior to IHCA

As evident in Figure 1C, which describes the clinical conditions preceding (up to 60 minutes) the cardiac arrest, hypoxia was more common among COVID+ cases (57%), as compared with COVID– cases (34%).

Survival analysis

The Kaplan Meier plots (Figure 2) show that COVID+ cases generally had a lower probability of survival compared to both COVID– and UNA cases. The overall 30-day survival (Figure 2A) was 21% among COVID+, compared with 36% in COVID– cases

(p=0.00086). The subgroup analysis of women (Figure 2B) showed low survival rates in COVID+ cases (16% 30-day survival). The subgroup analysis of men (Figure 2C) showed low survival rates in COVID+ cases (23% 30-day survival). The 30 days survival among COVID+ aged >70 years was 18% (Figure 2D), as compared with 25% of COVID+ cases aged 70 years or younger (Figure 2E). Survival curves for the subgroups of individuals with cancer, heart failure and diabetes, did not display any distinct patterns (Figure 2F-2H), with all p values >0.1. Patients with kidney failure had a 30 days survival of 13% among COVID+ cases (Figure 2I). Patients with acute MI had a 30 days survival of 8% among COVID+ cases (Figure 2J).

Cox adjusted survival curves are presented in Supplementary Figure 5; COVID+ cases displayed the lowest probability of survival, whereas there was no material difference between COVID– and UNA cases.

Odds ratios for 30-days survival

When adjusted for age, sex and initial rhythm the odds ratios for 30-day survival, comparing COVID+ vs. COVID–, were 0.50 (0.33-0.76) overall, 0.53 (0.31-0.88) for men, and 0.44 (0.20-0.88) for women. In the subgroup of patients with heart failure, myocardial infarction and cancer, we found no statistically significant associations, whereas in the subgroup of

COVID+ patients with kidney failure, odds ratio for 30-days survival was 0.43 (0.16–0.99), when compared with COVID– cases (Figure 3).

Predictors of survival

Regarding predictors of 30-days survival among COVID+ we note that confidence intervals were generally wide. Lack of ECG monitoring and delayed start of CPR showed point estimates below 1.0, although non-significant. Odds ratio for patients treated in non-monitored wards was 0.26 (95% CI 0.08-0.78) as compared with monitored wards (Figure 4). No coexisting condition was associated with survival among COVID+ cases.

Among COVID– cases, the factors that were significantly associated with 30-days survival were shockable rhythm (OR 4.18 [95% CI 2.69–6.02]), ECG monitoring (2.67 [95% CI 1.82–3.95]), heart failure (OR 0.58 [95% CI 0.40–0.83]) and diabetes (OR 0.64 [95% CI 0.44–0.92]; Figure 4).

Discussion

This study elucidates characteristics and outcomes in patients with COVID-19 who develop IHCA. We show that the prevalence of COVID-19 among patients suffering an IHCA increased to approximately one in four cardiac arrests during the first pandemic wave, and

one in five cardiac arrests during the second wave. In IHCA the probability of survival to 30-days is halved by the presence of COVID-19.

Regarding location of CA, we note that the most common location for COVID+ patients was regular wards, which are not monitored. This is unfortunate since our analyses showed that type of ward (monitored vs non-monitored) was significantly associated with survival, such that COVID+ cases in non-monitored wards displayed 74% lower probability of survival as compared with COVID+ cases in monitored wards. As compared with COVID– cases, cardiac arrest in the ER was more common in COVID+ cases. The often rapid deterioration of cardiopulmonary function in patients with COVID-19 may be one of the explanations for this finding. Fewer COVID+ cases were located in the CCU, which was an expected finding given that cardiac etiology was less common among these patients.

We note that the most common cause of cardiac arrest in COVID+ cases, as well as the most frequent clinical condition directly preceding the arrest, was respiratory. A total of 57% of cases displayed hypoxia before cardiac arrest. This may highlight an opportunity for improving outcomes; measures to prevent hypoxia and to correct it immediately may reduce the risk of cardiac arrest in patients with COVID-19. The high rate of respiratory etiology was driven by men (Supplementary Figure 6-7).

However, the fact that 43% of cases with COVID-19 did not have hypoxia prior to cardiac arrest suggests that other factors are important as well. Thromboembolism, myocardial infarction, arrhythmias, etc. may all contribute to the development of a cardiac arrest (12).

A previous study from Wuhan showed that 87.5% of COVID+ cases with IHCA had a respiratory etiology and a study from Southwest Georgia that 53% of the patients with IHCA and COVID-19 had ARDS (5, 7).

The survival rates were poor among COVID+ patients with an overall 30-days survival of 21%, compared to 36% among COVID–. The survival rate was, however, not as low as in the study from Wuhan, in which 3% (151 patients studied) survived, or in the study from New York with 31 patients or in the study from Southwest Georgia with 63 patients with none surviving (5, 7, 13). One reason for the poor survival could be the small number of patients found in shockable rhythm (17% vs. 25% for COVID+ and COVID–, respectively) since patients with shockable rhythm have a more favorable outcome. After adjusting for sex, age and shockable rhythm the 30-day survival was still significantly worse among patients with an ongoing infection.

We demonstrate that COVID+ women had halved chance of survival at 30 days, compared with COVID– women. We find it interesting that COVID+ women had acute MI three times

as often as men, despite the fact that men exhibited shockable rhythm – and were defibrillated – twice as often as women.

Strengths and limitations. This study includes all IHCAs in Sweden which were reported to SRCR. The sample recorded in the SRCR is unbiased since all hospitals participate in the registry and all hospitals report data on COVID-19 status. However, we do not know the severity of the COVID-19 infection, and we do not know if COVID-19 was the main reason for admission to hospital. With regards to the classification of COVID-19 status, we have performed a misclassification analysis which demonstrated that odds ratios were not materially affected by misclassification bias. Missingness was prevalent with regards to cause of cardiac arrest, which is due to the difficulties determining this factor. However, we find no reason to believe that missingness differs across COVID status categories, and it should therefore not bias our inferences. Our study only includes IHCAs receiving CPR. This leaves out all other patients with IHCA, e.g with a Do Not Attempt Resuscitation order.

Our regression models that included only COVID-19 cases should be interpreted with caution due to the large number of predictors in the model, with relatively few patients (resulting in wide confidence intervals). Further studies are warranted, using a larger study population, and

For peer review only - http://bmjopen.bmj.com/site/about/guidelines.xhtml

a longer follow up especially regarding subgroup analyses, neurological outcomes and the quality of life for these patients.

Conclusion

During pandemic peaks, up to one fourth of all IHCAs are complicated by COVID-19, and these patients have halved chance of survival, with women displaying the worst outcomes.

Funding

This work was supported by the Swedish Research Council [2019-02019] and the Swedish Heart and Lung Foundation [20200261].

Conflict of interest: none declared.

Author Statement: Astrid Holm and Araz Rawshani designed the study. Astrid Holm wrote the first draft of the manuscript, analyzed all data and made initial interpretations of data. Araz Rawshani has been supervising. Matilda Jerkeman, Pedram Sultanian, Peter Lundgren, Annica Ravn-Fischer, Johan Israelsson, Jasna Giesecke and Johan Herlitz revised the article critically for important intellectual content and approved the version of the article to be published.

Ethics statement: The study was approved by the Swedish Ethical Review Authority (ID 2020-02017). The data was anonymized before the authors accessed it for the purpose of the study.

Data sharing plan: No additional data available

References

1. Johns Hopkins University and Medicine COVID-19 map [internet] Johns Hopkins Coronavirus Resource Centre 2020. [updated 13/5/2021. Available from: https://coronavirus.jhu.edu/map.html.
2. Dödsorsaker första halvåret 2020. Socialstyrelsen. Hälso- och sjukvård.; 2020 17/11/2020.
3. Andersson J. Samhällsspridning av coronaviruset i Sverige. Läkartidningen. 16/03/2020.
4. Hayek SS, Brenner SK, Azam TU, Shadid HR, Anderson E, Berlin H, et al. In-hospital cardiac arrest in critically ill patients with covid-19: multicenter cohort study. Bmj. 2020;371:m3513.
5. Shah P, Smith H, Olarewaju A, Jani Y, Cobb A, Owens J, et al. Is Cardiopulmonary Resuscitation Futile in Coronavirus Disease 2019 Patients Experiencing In-Hospital Cardiac Arrest? Crit Care Med. 2021;49(2):201-8.
6. Mitchell OJL, Yuriditsky E, Johnson NJ, Doran O, Buckler DG, Neefe S, et al. In-hospital cardiac arrest in patients with coronavirus 2019. Resuscitation. 2021;160:72-8.
7. Shao F, Xu S, Ma X, Xu Z, Lyu J, Ng M, et al. In-hospital cardiac arrest outcomes among patients with COVID-19 pneumonia in Wuhan, China. Resuscitation. 2020;151:18-23.
8. Sultanian P, Lundgren P, Strömsöe A, Aune S, Bergström G, Hagberg E, et al. Cardiac arrest in COVID-19: characteristics and outcomes of in- and out-of-hospital cardiac arrest. A report from the Swedish Registry for Cardiopulmonary Resuscitation. European Heart Journal. 2021.
9. Hessulf F, Herlitz J, Rawshani A, Aune S, Israelsson J, Södersved-Källestedt ML, et al. Adherence to guidelines is associated with improved survival following in-hospital cardiac arrest. Resuscitation. 2020;155:13-21.
10. Stef van Buuren, Groothuis-Oudshoorn K. mice: Multivariate Imputation by Chained Equations in R. Journal of Statistical Software. 2011;Vol 45.
11. 3.5 Classification and regression trees [Available from: https://stefvanbuuren.name/fimd/sec-cart.html.
12. Gupta A, Madhavan MV, Sehgal K, Nair N, Mahajan S, Sehrawat TS, et al. Extrapulmonary manifestations of COVID-19. Nat Med. 2020;26(7):1017-32.
13. Sheth V, Chishti I, Rothman A, Redlener M, Liang J, Pan D, et al. Outcomes of in-hospital cardiac arrest in patients with COVID-19 in New York City. Resuscitation. 2020;155:3-5.

Table 1 Characteristics of 1613 patients with IHCA during the COVID-19 pandemic.

Variables	No infection COVID –	Ongoing infection COVID +	Unknown/NA UNA	SMD
n	1062	182	369	
**Demographics:**				
Age - mean (SD)	71.00 (13.32)	70.93 (12.43)	70.22 (13.60)	0.039
Woman - n (%)	388 (36.6)	68 (37.6)	151 (41.0)	0.061
**Location of cardiac arrest - n (%)**				0.527
Coronary care unit - n (%)	155 (14.6)	14 (7.7)	50 (13.6)	
Intensive care unit - n (%)	77 (7.3)	25 (13.7)	19 (5.1)	
Operational room - n (%)	22 (2.1)	0 (0.0)	12 (3.3)	
Emergency room - n (%)	139 (13.1)	29 (15.9)	65 (17.6)	
Outpatient lab, radiology - n (%)	49 (4.6)	7 (3.8)	28 (7.6)	
Cathlab - n (%)	98 (9.2)	8 (4.4)	60 (16.3)	
Intermediate care unit - n (%)	25 (2.4)	15 (8.2)	10 (2.7)	
Regular ward - n (%)	468 (44.1)	82 (45.1)	116 (31.4)	
Other - n (%)	29 (2.7)	2 (1.1)	9 (2.4)	
**Critical times - median (IQR):**				
Time to alert – median (IQR)	1.00 [1.00, 1.00]	1.00 [1.00, 1.00]	1.00 [1.00, 1.00]	0.078
Time to CPR - median (IQR)	0.00 [0.00, 1.00]	0.00 [0.00, 0.00]	0.00 [0.00, 1.00]	0.109
Time to defibrillation - median (IQR)	2.00 [1.00, 5.00]	2.00 [1.00, 4.75]	1.00 [1.00, 4.00]	0.141
**Comorbidities - n (%):**				
MI, ongoing - n (%)	178 (23.6)	12 (12.0)	37 (29.4)	0.292
MI, previous - n (%)	163 (20.8)	13 (11.7)	26 (18.4)	0.165
Stroke, ongoing - n (%)	30 (3.8)	4 (3.7)	4 (3.0)	0.030
Stroke, previous - n (%)	82 (10.3)	7 (6.1)	15 (10.5)	0.105
Cancer, any - n (%)	165 (20.9)	20 (17.7)	28 (20.6)	0.054
Diabetes - n (%)	224 (27.9)	36 (31.0)	38 (27.0)	0.060
Heart failure - n (%)	229 (29.7)	36 (33.0)	36 (27.9)	0.074
Ejection fraction (%) - mean (SD)	46.14 (13.74)	46.44 (11.86)	44.94 (14.82)	0.073
EF <50% - n (%)	167 (46.0)	26 (48.1)	22 (46.8)	0.029
**Kidney function category - n (%)**				0.121
eGFR <30 - n (%)	165 (21.6)	22 (20.0)	26 (20.0)	
eGFR 30–59- n (%)	216 (28.3)	32 (29.1)	44 (33.8)	
eGFR 60–89 - n (%)	198 (25.9)	25 (22.7)	30 (23.1)	
eGFR ≥90 - n (%)	185 (24.2)	31 (28.2)	30 (23.1)	
No kidney failure (eGFR ≥60) - n (%)	383 (50.1)	56 (50.9)	60 (46.2)	0.063
eGFR (ml/min/m2) - mean (SD)	66.89 (49.43)	71.26 (58.96)	63.78 (40.31)	0.099
**Cause of arrest:** - n (%)				0.629
Hemorrhage - n (%)	34 (4.9)	2 (2.0)	10 (8.1)	
Myocardial infarction/ischemia- n (%)	181 (26.2)	15 (14.9)	41 (33.3)	

infarction/ischemia 181 (26.2) 15 (14.9) 4 (33.				
Other - n (%)	213 (30.8)	30 (29.7)	41 (33.3)	
Primary arrhythmia - n (%)	101 (14.6)	8 (7.9)	12 (9.8)	
Respiratory insufficiency - n (%)	73 (10.5)	24 (23.8)	7 (5.7)	
Sepsis/infection - n (%)	45 (6.5)	19 (18.8)	4 (3.3)	
Stroke/thromboembolism - n (%)	45 (6.5)	3 (3.0)	8 (6.5)	
**Early interventions - n (%):**				
Witnessed arrest - n (%)	857 (80.9)	140 (77.8)	306 (85.0)	0.124
ECG monitoring - n (%)	635 (60.5)	89 (50.0)	221 (62.1)	0.163
CPR before AGA - n (%)	845 (91.0)	146 (93.6)	268 (88.2)	0.127
Defibrillated before AGA – n (%)	159 (17.9)	18 (11.9)	53 (19.0)	0.131
Ventilated before AGA - n (%)	503 (63.2)	74 (54.8)	175 (69.2)	0.199
Shockable rhythm - n (%)	247 (24.9)	29 (17.3)	90 (27.0)	0.158
Defibrillated, any - n (%)	323 (31.5)	40 (22.7)	111 (32.8)	0.151
Intubated - n (%)	473 (47.0)	100 (57.8)	177 (53.8)	0.145
Adrenaline given - n (%)	668 (65.6)	125 (72.7)	223 (66.4)	0.102
Antiarrhythmics - n (%)	139 (14.1)	17 (10.1)	48 (15.4)	0.107
Mechanical compressions – n (%)	109 (10.8)	18 (10.4)	66 (20.0)	0.180
Active temperature control – n (%)	54 (11.3)	5 (10.4)	3 (4.4)	0.173
**Status at rescue team arrival - n (%):**				
Consciousness - n (%)	214 (23.1)	18 (11.7)	57 (19.3)	0.204
Breathing - n (%)	288 (31.2)	30 (19.5)	84 (28.7)	0.181
Pulse - n (%)	309 (33.8)	36 (23.4)	89 (30.4)	0.154
**Follow-Up data - n (%):**				
Angiography - n (%)	115 (24.2)	8 (16.7)	15 (20.8)	0.124
PCI - n (%)	87 (18.2)	4 (8.3)	16 (21.9)	0.258
Pacemaker implanted - n (%)	80 (16.7)	2 (4.2)	4 (5.6)	0.281
ICD implanted - n (%)	36 (7.5)	1 (2.1)	2 (2.8)	0.172
ROSC - n (%)	520 (49.0)	64 (35.2)	142 (38.5)	0.188
Death at 30 days - n (%)	666 (62.7)	141 (77.5)	237 (64.2)	0.218
Death overall - n (%)	703 (66.2)	141 (77.5)	241 (65.3)	0.181

SD = standard deviation; IQR = interquartile range; SMD = standardized mean difference (difference between the means for the two groups divided by their mutual standard deviation. Values below 0.1 (10%) are considered inconsequential (i.e., no significant difference between the groups)). CPR = Cardiopulmonary resuscitation, PCI = Percutaneous Coronary Intervention, ICD = implantable cardioverter-defibrillator. ROSC = return of spontaneous circulation. AGA= alarm group arrival

Figure Titles and Legends

Figure 1: Characteristics of IHCA according to COVID-19 status

A: Monthly proportion of COVID-19 status among patients with IHCA, stratified on COVID-19 status. In March only cases after 15/03/2020 were included.

B: Etiology of IHCA, stratified on COVID-19 status. The y-axis shows percentages for each etiology in each group.

C: Clinical conditions 1 hour prior to IHCA, stratified on COVID-19 status. Only patients with data regarding the specific condition was included.

Figure 2: Kaplan Meier survival curves

Kaplan Meier survival curves, separately for (A)Overall, (B)Women, (C)Men, (D)Age ≥70 year, (E)Age <70 year, (F)Cancer, (G)Heart failure, (H)Diabetes, (I)Kidney failure and (J)Myocardial infarction. p= log-rank p-value. The numbers under the graphs are showing the survival in percentages. Regarding myocardial infarction acute MI is presented.

Figure 3: Odds Ratio for 30-day survival

Forest plot with the adjusted odds ratio for 30-day survival among patients with ongoing infection vs. no infection and unknown/NA vs. no infection. Stratified on overall, men, women, age < 70 years, age ≥ 70 years, heart failure, kidney failure, diabetes, myocardial infarction and cancer. Myocardial infarction was defined as acute or previous MI.

Figure 4: Odds Ratio for 30-day survival

Forest plot with odds ratio for 30-day survival, stratified on the groups, no infection, ongoing infection and overall, all in different colors. The 95% Confidence interval is shown between the bars. X-axis has a logarithmic scale. ECG=electrocardiogram, CA=cardiac arrest, MI=myocardial infarction. CI=confidence interval.

Figure 1: Characteristics of IHCA according to COVID-19 status

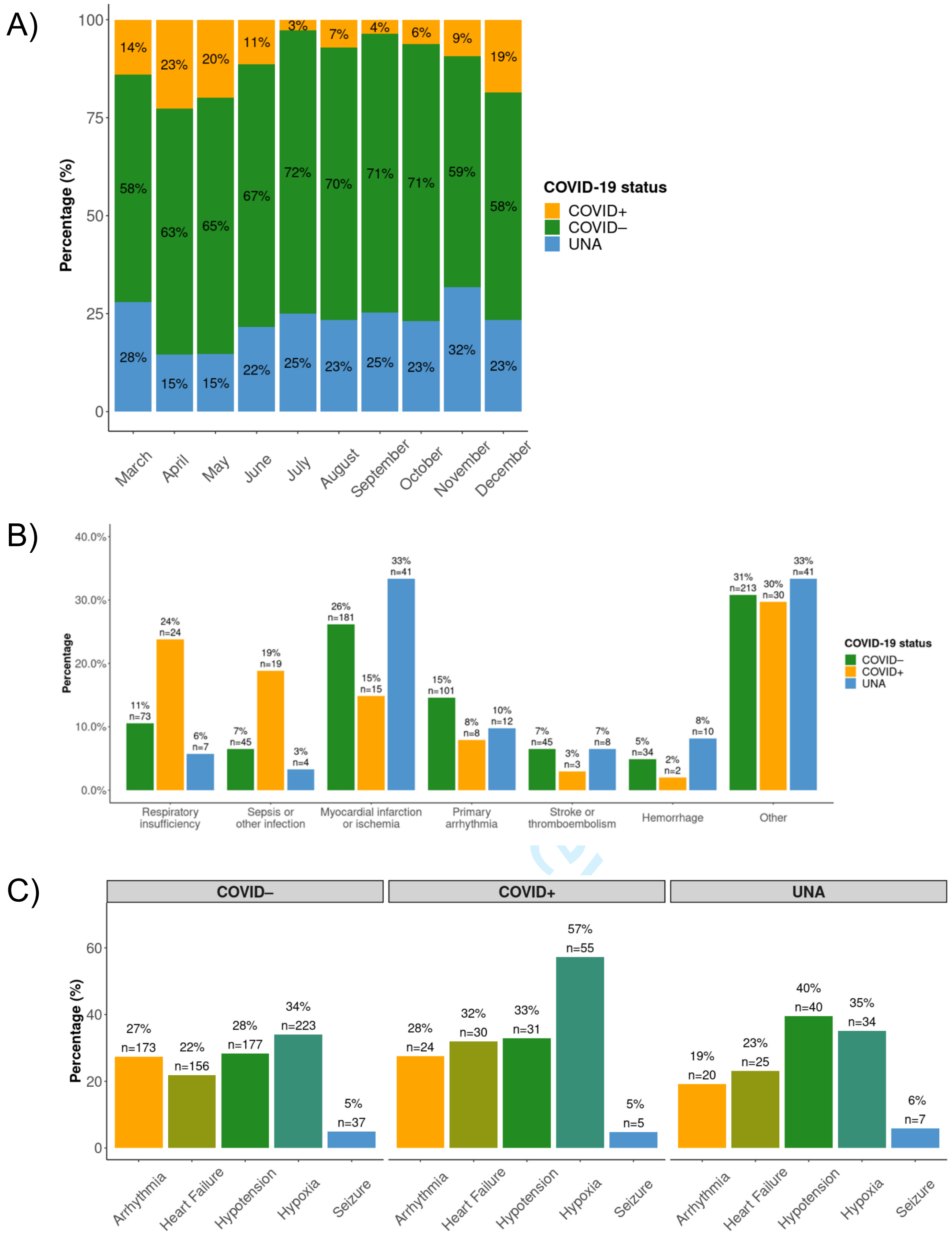

Figure 1A: Monthly proportion of COVID-19 status among patients with IHCA, stratified on COVID-19 status. In March only cases after 15/03/2020 were included.

Figure 1B: Etiology of IHCA, stratified on COVID-19 status. The y-axis shows percentages for each etiology in each group.

Figure 1C: Clinical conditions 1 hour prior to IHCA, stratified on COVID-19 status. Only patients with data regarding the specific condition was included.

Figure 2: Kaplan Meier survival curves

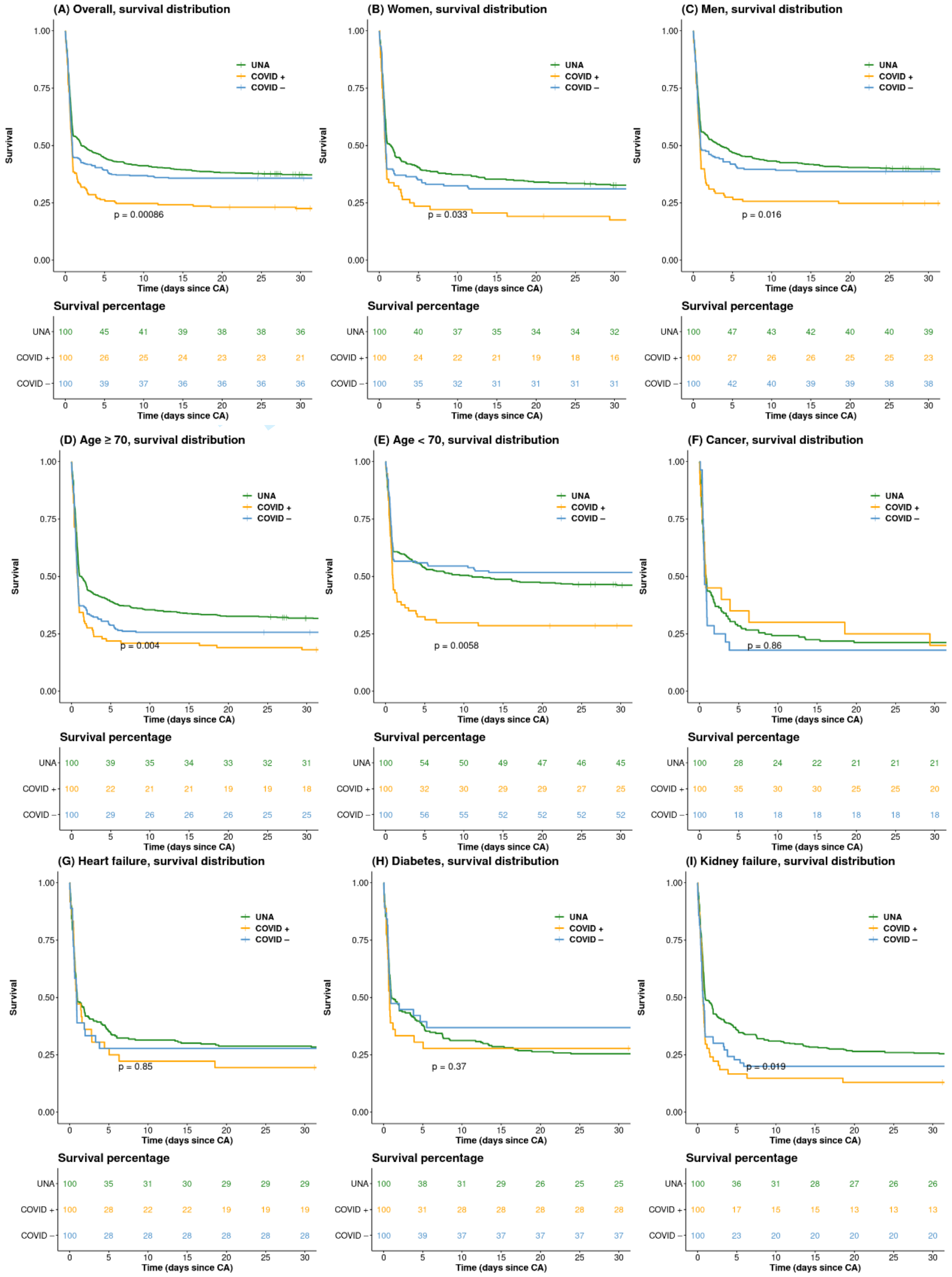

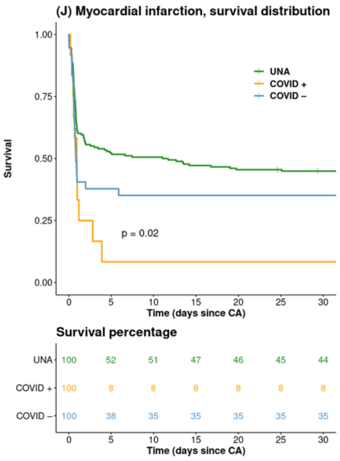

Figure 2: Kaplan Meier survival curves, separately for (A) Overall, (B) Women, (C) Men, (D) Age ≥70 year, (E) Age <70 year, (F) Cancer, (G) Heart failure, (H) Diabetes, (I) Kidney failure and (J) Myocardial infarction. p= log-rank p-value. The numbers under the graphs are showing the survival in percentages. Regarding myocardial infarction acute MI is

Figure 3: Odds Ratio for 30-day survival

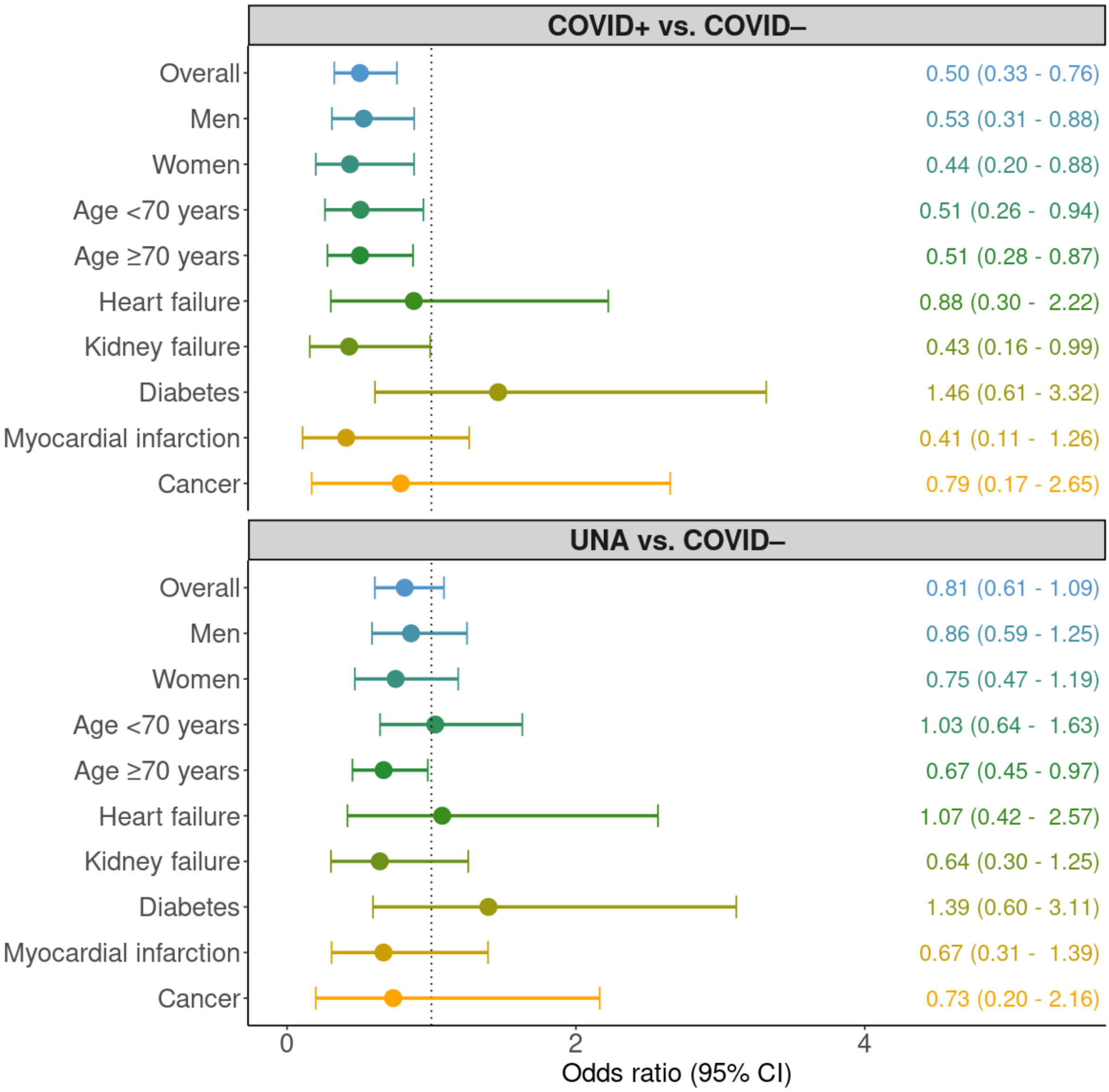

Figure 3: Forest plot with the adjusted odds ratio for 30-day survival among patients with ongoing infection vs. no infection and unknown/NA vs. no infection. Stratified on overall, men, women, age < 70 years, age ≥ 70 years, heart failure, kidney failure, diabetes, myocardial infarction and cancer. Myocardial infarction was defined as acute or previous MI.

Figure 4: Odds Ratio for 30-day survival

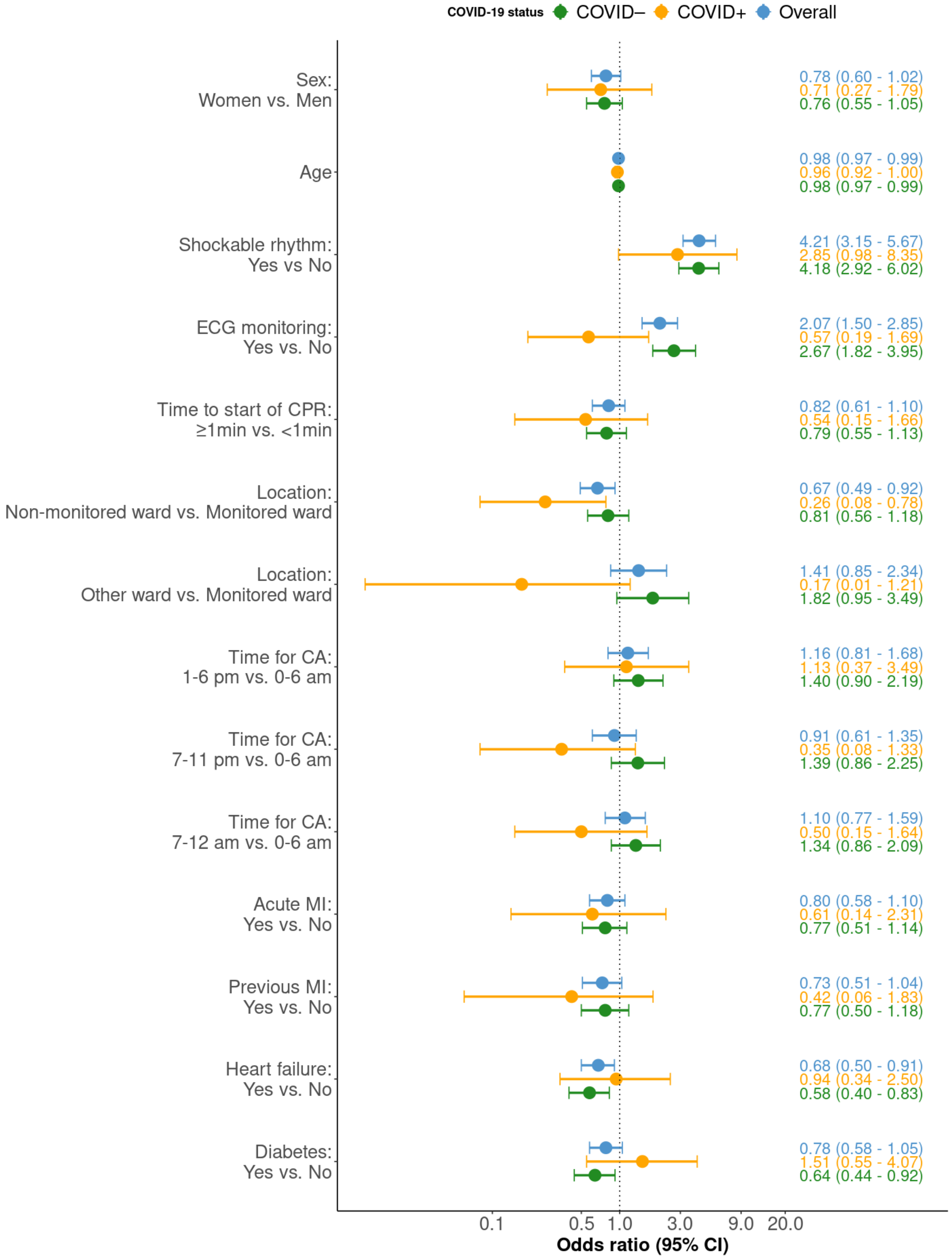

Figure 4: Forest plot with odds ratio for 30-day survival, stratified on the groups, no infection, ongoing infection and overall, all in different colors. The 95% Confidence interval is shown between the bars. X-axis has a logarithmic scale. ECG= electrocardiogram, CA= cardiac arrest, MI= myocardial infarction. CI= confidence interval.

Supplementary figures and tables

Characteristics and Outcomes in Patients with COVID-19 and In-Hospital Cardiac Arrest

For peer review only

Supplementary Table 1: Characteristics of COVID+ patients with IHCA in relation to sex.

Supplementary Table 1: Characteristics of 181 COVID+ patients with IHCA during the COVID-19 pandemic in relation to sex. One COVID+ patient had missing data on sex.

variables	Men	Women	SMD
n	113	68	
**Demographics:**			
Age - mean (SD)	71.39 (10.75)	70.35 (14.87)	0.080
**Location of cardiac arrest - n (%):**			0.249
Coronary care unit	7 (6.2)	7 (10.3)	
Intensive care unit	15 (13.3)	10 (14.7)	
Operational room	0 (0.0)	0 (0.0)	
Emergency room	17 (15.0)	11 (16.2)	
Outpatient lab, radiology	4 (3.5)	3 (4.4)	
Cathlab	6 (5.3)	2 (2.9)	
Intermediate care unit	11 (9.7)	4 (5.9)	
Regular ward	52 (46.0)	30 (44.1)	
Other	1 (0.9)	1 (1.5)	
**Critical times - median (IQR):**			
Time to alert – median (IQR)	1.00 [1.00, 1.00]	1.00 [1.00, 1.00]	0.256
Time to CPR - median (IQR)	0.00 [0.00, 0.00]	0.00 [0.00, 1.00]	0.031
Time to defibrillation - median	2.00 [1.00, 5.00]	2.00 [1.00, 2.00]	0.009
**Comorbidities - n (%):**			
MI, ongoing - n (%)	5 (7.6)	7 (21.2)	0.396
MI, previous - n (%)	11 (16.4)	2 (4.7)	0.391
Stroke, ongoing - n (%)	4 (5.8)	0 (0.0)	0.351
Stroke, previous - n (%)	5 (7.0)	2 (4.7)	0.102
Cancer, any - n (%)	13 (18.8)	6 (14.0)	0.132
Diabetes - n (%)	24 (33.3)	12 (27.9)	0.118
Heart failure - n (%)	23 (33.8)	13 (32.5)	0.028
Ejection fraction (%) - mean (SD)	44.84 (12.22)	49.31 (10.56)	0.392
EF <50% - n (%)	19 (51.4)	7 (43.8)	0.153
**Kidney function category - n (%):**			0.357
eGFR <30	16 (22.9)	6 (15.0)	
eGFR 30–59	17 (24.3)	15 (37.5)	
eGFR 60–89	18 (25.7)	7 (17.5)	
eGFR ≥90	19 (27.1)	12 (30.0)	
No kidney failure (eGFR ≥60)	37 (52.9)	19 (47.5)	0.107
eGFR (ml/min/m2) - mean (SD)	72.72 (65.75)	68.70 (45.34)	0.071
**Cause of arrest - n (%):**			0.920
Hemorrhage	1 (1.5)	1 (2.9)	
Myocardial infarction/ischemia	7 (10.6)	8 (23.5)	
Other	18 (27.3)	12 (35.3)	
Primary arrhythmia	3 (4.5)	5 (14.7)	
Respiratory insufficiency	17 (25.8)	7 (20.6)	

Sepsis / infection	18 (27.3)	1 (2.9)	
Stroke / thromboembolism	2 (3.0)	0 (0.0)	
**Early interventions - n (%):**			
Witnessed arrest - n (%)	86 (76.8)	53 (79.1)	0.056
ECG monitoring - n (%)	56 (50.5)	33 (50.0)	0.009
CPR before AGA - n (%)	90 (92.8)	55 (94.8)	0.085
Defibrillated before AGA - n (%)	13 (13.8)	5 (8.9)	0.155
Ventilated before AGA- n (%)	49 (56.3)	25 (53.2)	0.063
Shockable rhythm - n (%)	22 (20.8)	7 (11.5)	0.254
Defibrillated, any - n (%)	29 (26.4)	11 (16.9)	0.231
Intubated - n (%)	61 (57.0)	38 (58.5)	0.029
Adrenaline given - n (%)	76 (70.4)	48 (76.2)	0.132
Antiarrhythmics - n (%)	11 (10.4)	6 (9.7)	0.023
Mechanical compressions - n (%)	12 (10.9)	5 (8.1)	0.097
Active temperature control - n (%)	2 (6.1)	3 (20.0)	0.423
**Status at rescue team arrival - n**			
Consciousness - n (%)	11 (11.3)	6 (10.7)	0.020
Breathing - n (%)	18 (18.6)	11 (19.6)	0.028
Pulse - n (%)	22 (22.7)	13 (23.2)	0.013
**Follow-Up data - n (%):**			
Angiography - n (%)	4 (12.1)	4 (26.7)	0.374
PCI - n (%)	2 (6.1)	2 (13.3)	0.248
Pacemaker implanted - n (%)	0 (0.0)	2 (13.3)	0.555
ICD implanted - n (%)	0 (0.0)	1 (6.7)	0.378
ROSC - n (%)	40 (35.4)	24 (35.3)	0.002
Death at 30 days - n (%)	85 (75.2)	56 (82.4)	0.175
Death overall - n (%)	85 (75.2)	56 (82.4)	0.175
Discharged alive - n (%)	16 (22.2)	6 (14.0)	0.216

SD = standard deviation; IQR = interquartile range; SMD = standardized mean difference (difference between the means for the two groups divided by their mutual standard deviation. Values below 0.1 (10%) are considered inconsequential (i.e., no significant difference between the groups)). CPR = cardiopulmonary resuscitatio*n*, PCI = percutaneous coronary intervention, ICD = implantable cardioverter-defibrillator. ROSC = return of spontaneous circulation. AGA= alarm group arrival.

Supplementary Figure 1: Missing data before and after imputation with MICE

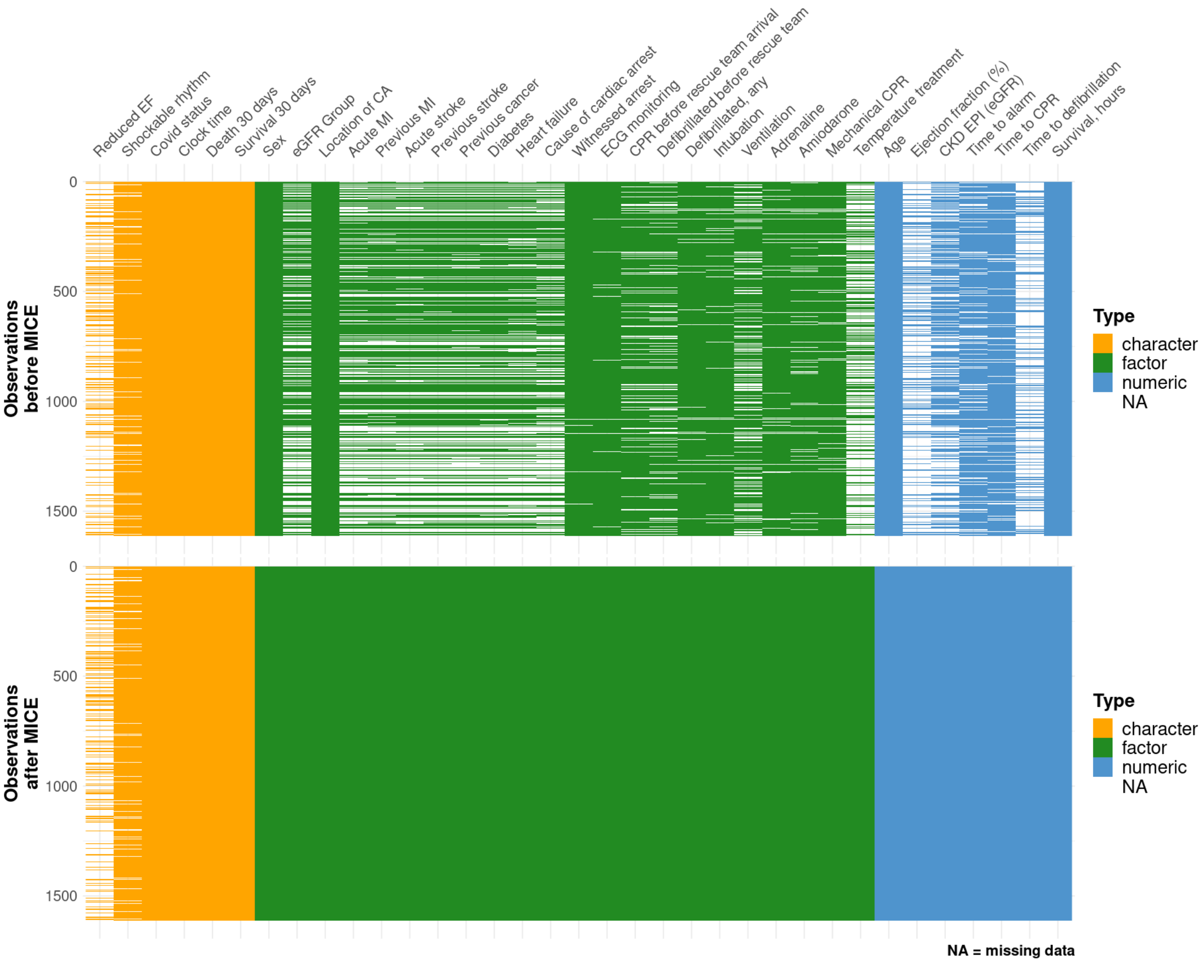

Supplementary Figure 1: Missing data before and after imputation with MICE. A graphical view of the entire dataset is printed. Each column (variable) is depicted at the top and column color depicts type of variable. Each patient represents a row and white gaps indicate a missing data entry.

Supplementary Figure 2: Flow chart

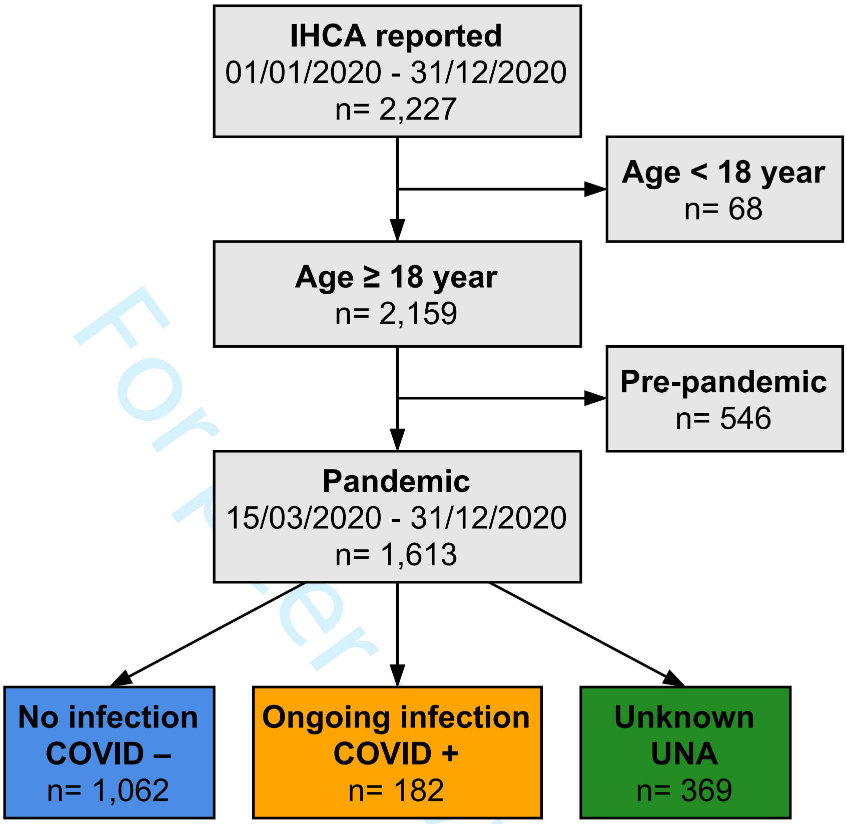

Supplementary Figure 2: Flow chart of the study population. Patients who were less than 18 year of age, and cases occurring in the pre-pandemic period were excluded.

For peer review only

Supplementary Figure 3: Information on COVID-19 status during the study period.

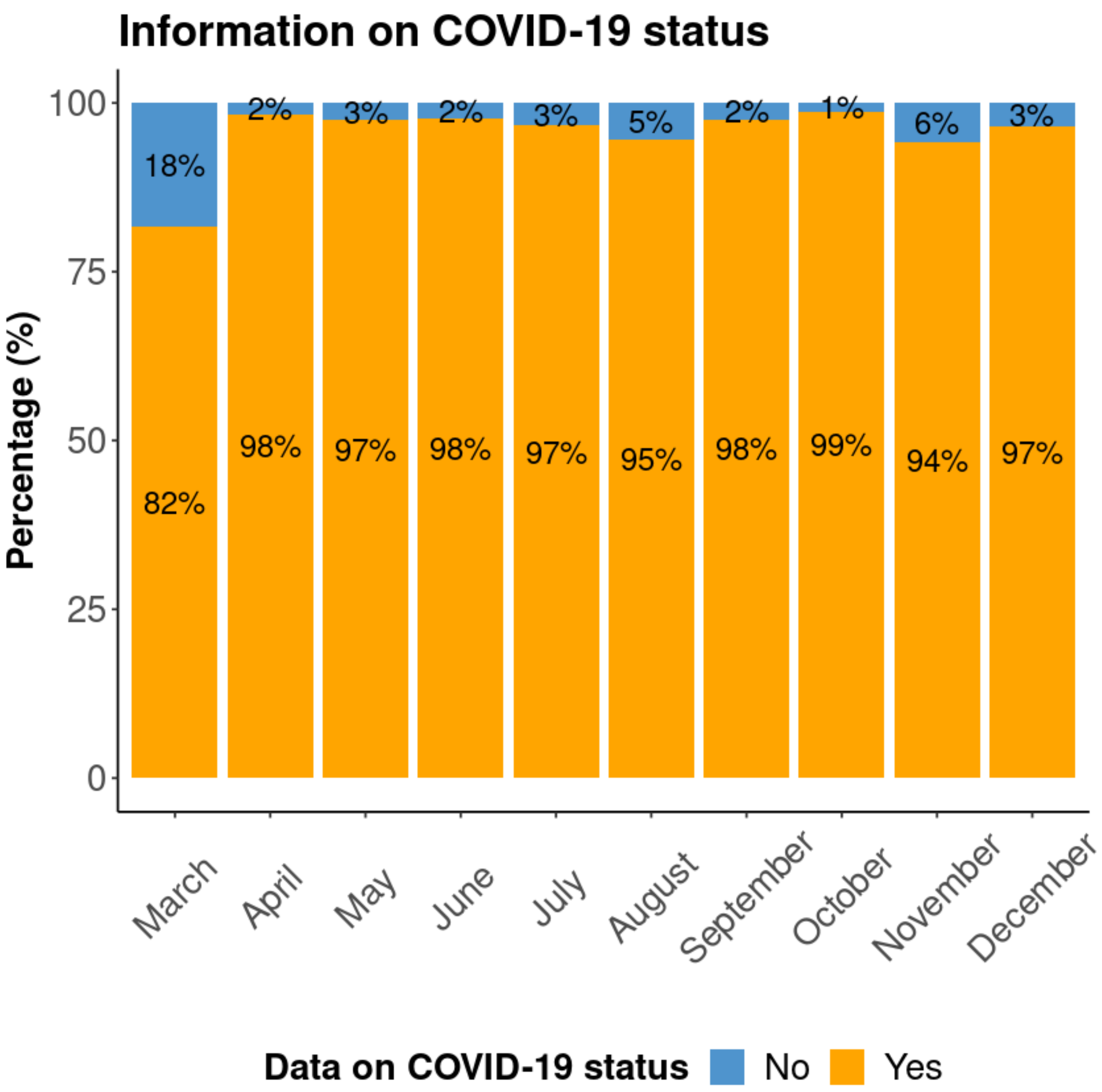

Supplementary Figure 3: Information on COVID-19 status during the study period. No equals missing data, i.e. no information on COVID-19 status available. Yes equals, COVID +, COVID – or Unknown. In March only cases after 15/03/2020 were included.

Supplementary Figure 4: Distribution of age

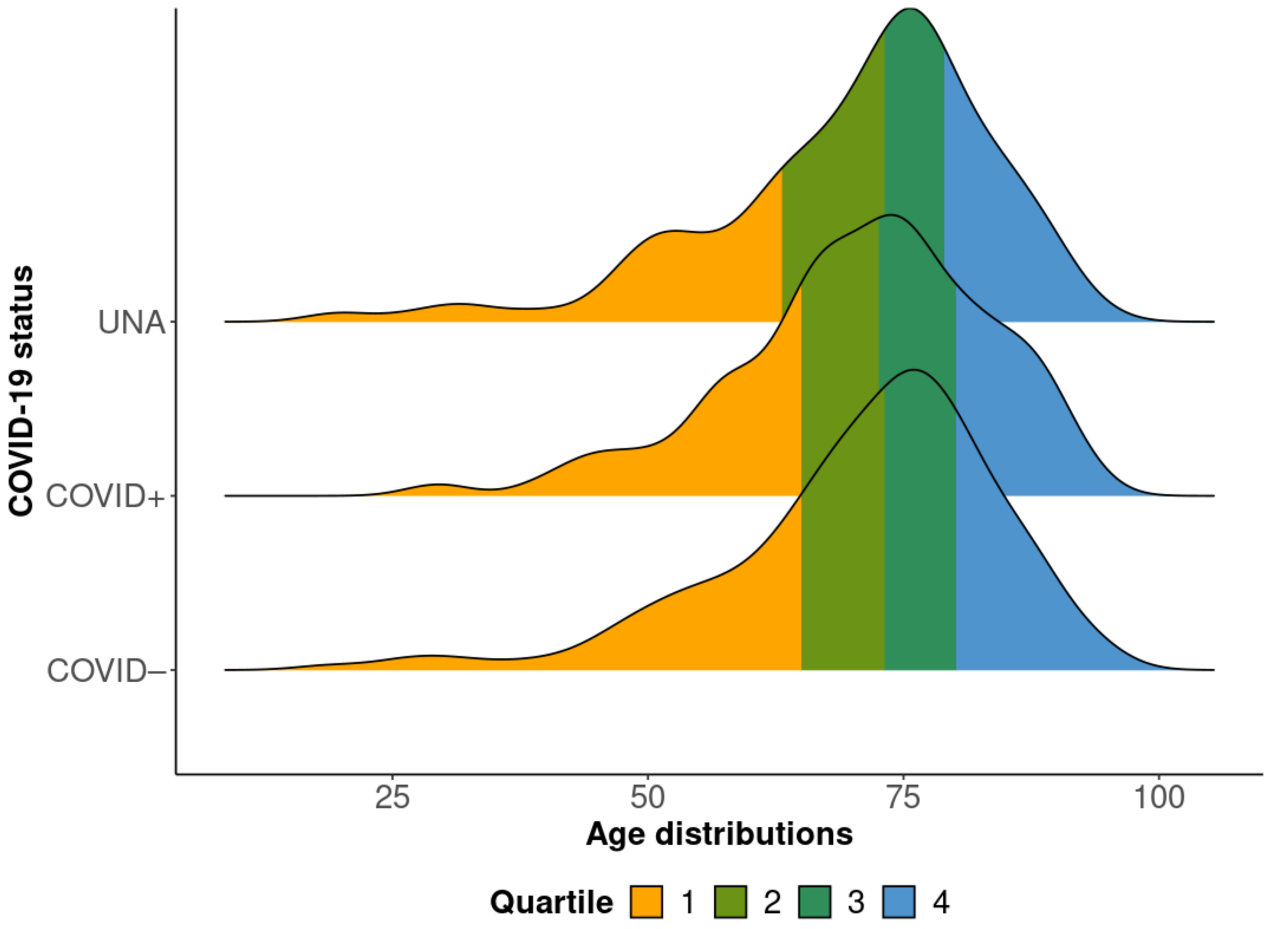

Supplementary Figure 4: Distribution of age in relation to COVID-19 status.

Supplementary Figure 5: Etiology of IHCA, according to sex

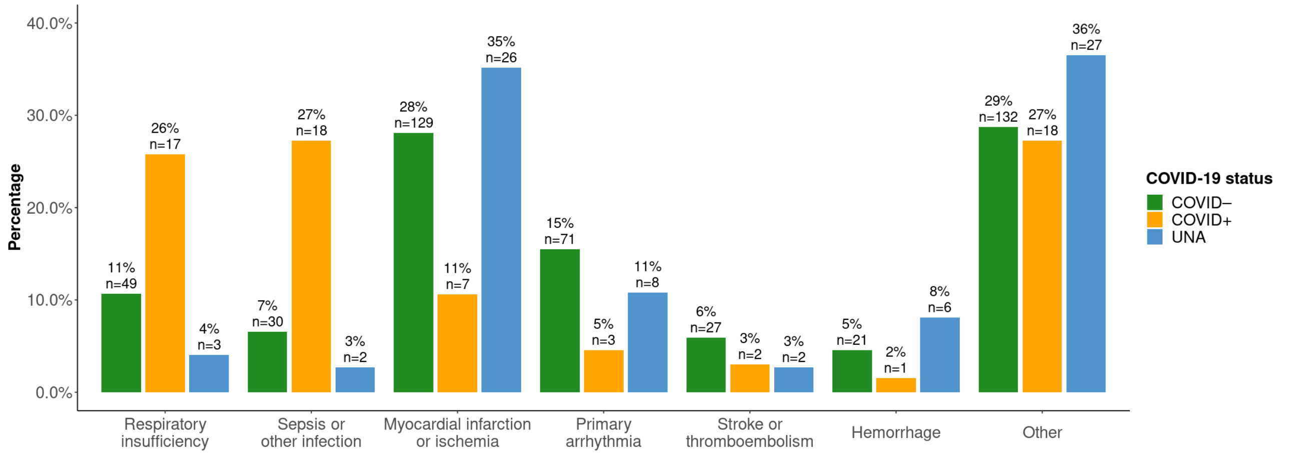

Supplementary Figure 5A: Etiology of IHCA, men only.

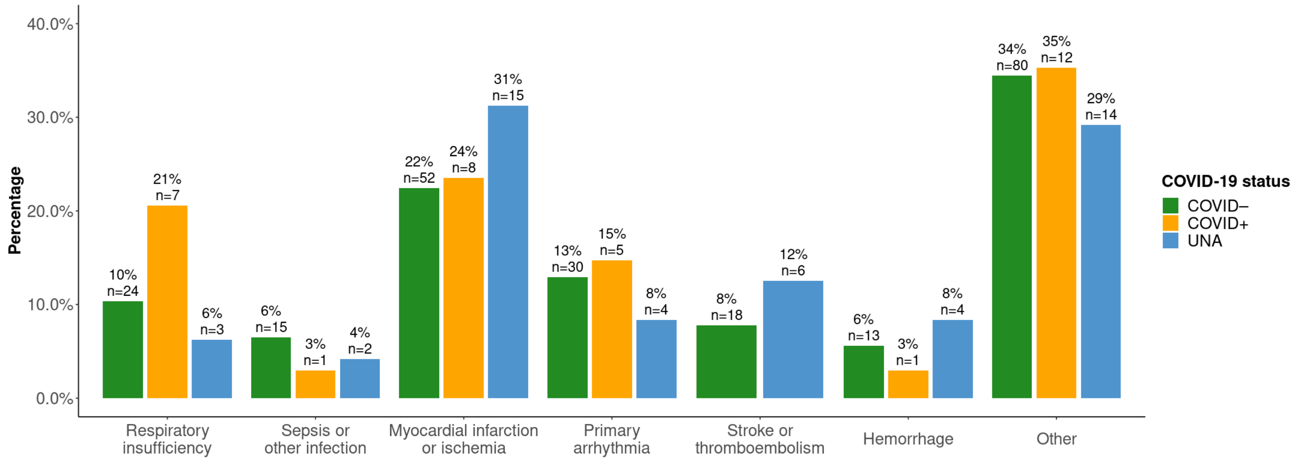

Supplementary Figure 5B: Etiology of IHCA, women only.

Supplementary Figure 6: Conditions preceding IHCA, according to sex

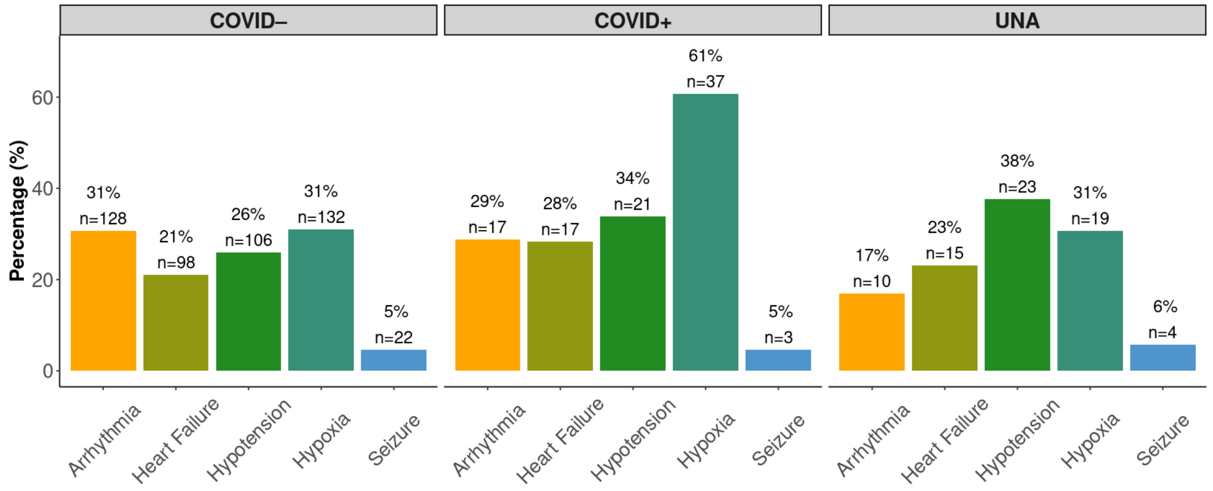

Supplementary Figure 6A: Conditions preceding IHCA, men only.

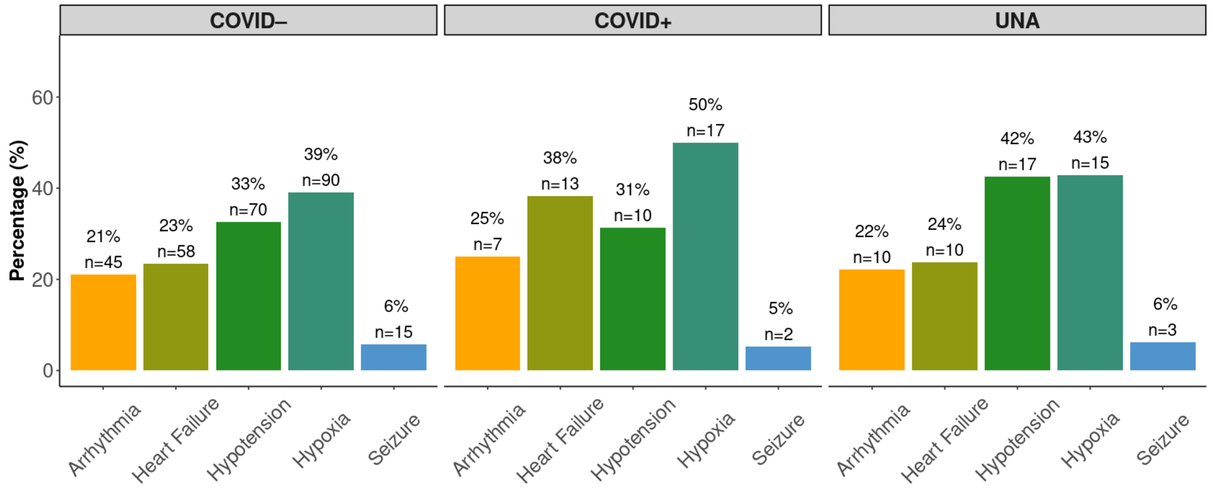

Supplementary Figure 6B: Conditions preceding IHCA, women only.

For peer review only - http://bmjopen.bmj.com/site/about/guidelines.xhtml

Supplementary Figure 7: Cox adjusted survival curve for the overall population

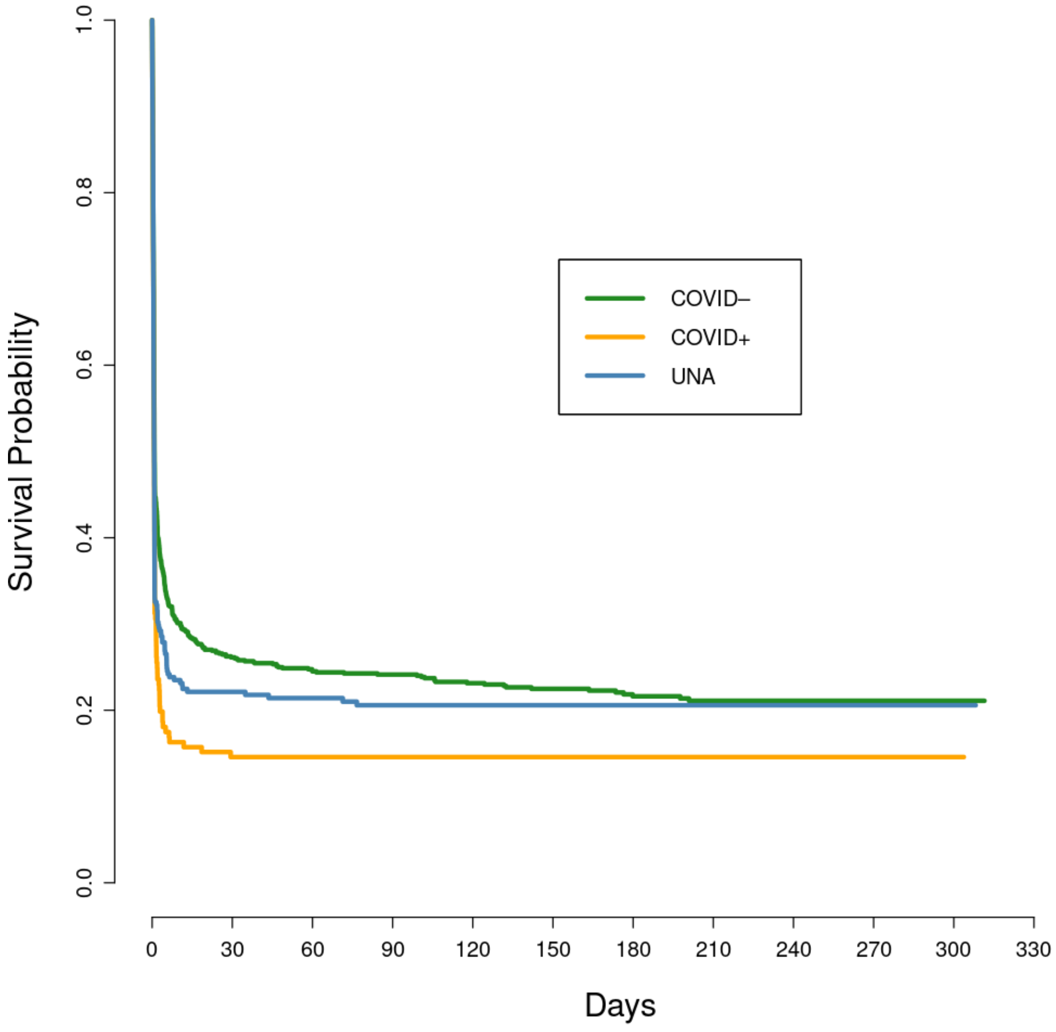

Supplementary Figure 7: Cox adjusted survival curve for the overall population, stratified on COVID-19 status.

only

STROBE Statement—checklist of items that should be included in reports of observational studies

	Item No	Recommendation	Page No
**Title and abstract**	1	(*a*) Indicate the study's design with a commonly used term in the title or the abstract	1
		(*b*) Provide in the abstract an informative and balanced summary of what was done and what was found	2-3
**Introduction**			
Background/rationale	2	Explain the scientific background and rationale for the investigation being reported	2
Objectives	3	State specific objectives, including any prespecified hypotheses	2
**Methods**			
Study design	4	Present key elements of study design early in the paper	2
Setting	5	Describe the setting, locations, and relevant dates, including periods of recruitment, exposure, follow-up, and data collection	2
Participants	6	(*a*) *Cohort study*—Give the eligibility criteria, and the sources and methods of selection of participants. Describe methods of follow-up<br>*Case-control study*—Give the eligibility criteria, and the sources and methods of case ascertainment and control selection. Give the rationale for the choice of cases and controls<br>*Cross-sectional study*—Give the eligibility criteria, and the sources and methods of selection of participants	2
		(*b*) *Cohort study*—For matched studies, give matching criteria and number of exposed and unexposed<br>*Case-control study*—For matched studies, give matching criteria and the number of controls per case	2
Variables	7	Clearly define all outcomes, exposures, predictors, potential confounders, and effect modifiers. Give diagnostic criteria, if applicable	5-6
Data sources/ measurement	8*	For each variable of interest, give sources of data and details of methods of assessment (measurement). Describe comparability of assessment methods if there is more than one group	5
Bias	9	Describe any efforts to address potential sources of bias	3-4
Study size	10	Explain how the study size was arrived at	5
Quantitative variables	11	Explain how quantitative variables were handled in the analyses. If applicable, describe which groupings were chosen and why	5-6
Statistical methods	12	(*a*) Describe all statistical methods, including those used to control for confounding	6-7
		(*b*) Describe any methods used to examine subgroups and interactions	6-7
		(*c*) Explain how missing data were addressed	6-7
		(*d*) *Cohort study*—If applicable, explain how loss to follow-up was addressed<br>*Case-control study*—If applicable, explain how matching of cases and controls was addressed<br>*Cross-sectional study*—If applicable, describe analytical methods taking account of sampling strategy	6-7
		(*e*) Describe any sensitivity analyses	6-7

Continued on next page

For peer review only

**Results**			
Participants	13*	(a) Report numbers of individuals at each stage of study—eg numbers potentially eligible, examined for eligibility, confirmed eligible, included in the study, completing follow-up, and analysed	7
		(b) Give reasons for non-participation at each stage	7
		(c) Consider use of a flow diagram	Supplementary material
Descriptive data	14*	(a) Give characteristics of study participants (eg demographic, clinical, social) and information on exposures and potential confounders	7-8
		(b) Indicate number of participants with missing data for each variable of interest	7-8
		(c) *Cohort study*—Summarise follow-up time (eg, average and total amount)	7
Outcome data	15*	*Cohort study*—Report numbers of outcome events or summary measures over time	*8-9*
		Case-control study—Report numbers in each exposure category, or summary measures of exposure	
		Cross-sectional study—Report numbers of outcome events or summary measures	
Main results	16	(*a*) Give unadjusted estimates and, if applicable, confounder-adjusted estimates and their precision (eg, 95% confidence interval). Make clear which confounders were adjusted for and why they were included	7-10
		(*b*) Report category boundaries when continuous variables were categorized	
		(*c*) If relevant, consider translating estimates of relative risk into absolute risk for a meaningful time period	
Other analyses	17	Report other analyses done—eg analyses of subgroups and interactions, and sensitivity analyses	Supplementary material
**Discussion**			
Key results	18	Summarise key results with reference to study objectives	11
Limitations	19	Discuss limitations of the study, taking into account sources of potential bias or imprecision. Discuss both direction and magnitude of any potential bias	13-14
Interpretation	20	Give a cautious overall interpretation of results considering objectives, limitations, multiplicity of analyses, results from similar studies, and other relevant evidence	11-14
Generalisability	21	Discuss the generalisability (external validity) of the study results	14
**Other information**			
Funding	22	Give the source of funding and the role of the funders for the present study and, if applicable, for the original study on which the present article is based	14

*Give information separately for cases and controls in case-control studies and, if applicable, for exposed and unexposed groups in cohort and cross-sectional studies.

Note: An Explanation and Elaboration article discusses each checklist item and gives methodological background and published examples of transparent reporting. The STROBE checklist is best used in conjunction with this article (freely

For peer review only - http://bmjopen.bmj.com/site/about/guidelines.xhtml

available on the Web sites of PLoS Medicine at http://www.plosmedicine.org/, Annals of Internal Medicine at http://www.annals.org/, and Epidemiology at http://www.epidem.com/). Information on the STROBE Initiative is available at www.strobe-statement.org.

For peer review only

For peer review only - http://bmjopen.bmj.com/site/about/guidelines.xhtml

BMJ Open

A Cohort Study of the Characteristics and Outcomes in Patients with COVID-19 and In-Hospital Cardiac Arrest

Journal:	*BMJ Open*
Manuscript ID	bmjopen-2021-054943.R3
Article Type:	Original research
Date Submitted by the Author:	13-Nov-2021
Complete List of Authors:	Holm, Astrid; University of Gothenburg, Department of Molecular and Clinical Medicine<br>Jerkeman, Matilda; University of Gothenburg, Department of Molecular and Clinical Medicine<br>Sultanian, Pedram; University of Gothenburg, Department of Molecular and Clinical Medicine<br>Lundgren, Peter; University of Gothenburg, Department of Molecular and Clinical Medicine; Kalmar County Hospital, Department of Internal Medicine<br>Ravn-Fischer, Annica; University of Gothenburg, Department of Molecular and Clinical Medicine<br>Israelsson, Johan; Kalmar County Hospital, Department of Internal Medicine; Linnaeus University, Faculty of Health and Life Sciences<br>Giesecke, Jasna; Danderyd University Hospital<br>Herlitz, Johan; University of Borås, Centre for Prehospital Research<br>Rawshani, Araz; University of Gothenburg, Department of Molecular and Clinical Medicine
**Primary Subject Heading**:	Cardiovascular medicine
Secondary Subject Heading:	Cardiovascular medicine, Infectious diseases
Keywords:	COVID-19, CARDIOLOGY, Adult cardiology < CARDIOLOGY, Coronary heart disease < CARDIOLOGY

SCHOLARONE™ Manuscripts

I, the Submitting Author has the right to grant and does grant on behalf of all authors of the Work (as defined in the below author licence), an exclusive licence and/or a non-exclusive licence for contributions from authors who are: i) UK Crown employees; ii) where BMJ has agreed a CC-BY licence shall apply, and/or iii) in accordance with the terms applicable for US Federal Government officers or employees acting as part of their official duties; on a worldwide, perpetual, irrevocable, royalty-free basis to BMJ Publishing Group Ltd ("BMJ") its licensees and where the relevant Journal is co-owned by BMJ to the co-owners of the Journal, to publish the Work in this journal and any other BMJ products and to exploit all rights, as set out in our licence.

The Submitting Author accepts and understands that any supply made under these terms is made by BMJ to the Submitting Author unless you are acting as an employee on behalf of your employer or a postgraduate student of an affiliated institution which is paying any applicable article publishing charge ("APC") for Open Access articles. Where the Submitting Author wishes to make the Work available on an Open Access basis (and intends to pay the relevant APC), the terms of reuse of such Open Access shall be governed by a Creative Commons licence – details of these licences and which Creative Commons licence will apply to this Work are set out in our licence referred to above.

Other than as permitted in any relevant BMJ Author's Self Archiving Policies, I confirm this Work has not been accepted for publication elsewhere, is not being considered for publication elsewhere and does not duplicate material already published. I confirm all authors consent to publication of this Work and authorise the granting of this licence.

For peer review only

A Cohort Study of the Characteristics and Outcomes in Patients with COVID-19 and In-Hospital Cardiac Arrest

Astrid Holm, MD[1], Matilda Jerkeman, MD[1], Pedram Sultanian, MD[1], Peter Lundgren, MD, PhD[1,3,7], Annica Ravn-Fischer, MD, PhD, Docent[1], Johan Israelsson, PhD[3,4], Jasna Giesecke, RN[5], Johan Herlitz, MD, PhD[2,6], Araz Rawshani, MD, PhD[1,2]

Affiliations:

[1]Department of Molecular and Clinical Medicine, Institute of Medicine, Sahlgrenska Academy, University of Gothenburg, Sweden

[2]The Swedish Registry for Cardiopulmonary Resuscitation, Centre of Registries, Västra Götaland County, Sweden

[3]Department of Internal Medicine, Division of Cardiology, Kalmar County Hospital, Region Kalmar County, Sweden

[4]Faculty of Health and Life Sciences, Linnaeus University, Kalmar, Sweden

[5]RN, Lead CPR coordinator, Clinicum- Centre for clinical skills, interprofessional education and advanced medical simulation, Danderyd University Hospital, Stockholm, Sweden

[6]Prehospen – Centre for Prehospital Research, University of Borås, Borås, Sweden

[7]Region Västra Götaland, Sahlgrenska University Hospital, Department of Cardiology, Gothenburg, Sweden

Contact information:

Astrid Holm

Email: astrid.holm@gu.se

Phone: +358 400707722

Manuscript word count: 3088

Abstract

Objective: We studied characteristics, survival, causes of cardiac arrest, conditions preceding cardiac arrest, predictors of survival, and trends in the prevalence of COVID-19 among in-hospital cardiac arrest (IHCA) cases.

Design and setting: Registry-based observational study.

Participants: We studied all cases (≥18 years of age) of IHCA receiving cardiopulmonary resuscitation (CPR) in the Swedish Registry for Cardiopulmonary Resuscitation during 15/03/2020 to 31/12/2020. A total of 1613 patients were included and divided into the following groups: ongoing infection (**COVID+;** n=182), no infection (**COVID–;** n=1062) and unknown/not assessed (**UNA**; n=369).

Main outcomes and measures: We studied monthly trends in proportions of COVID-19 associated IHCAs, causes of IHCA in relation to COVID-19 status, clinical conditions preceding the cardiac arrest and predictors of survival.

Results: The rate of COVID+ patients suffering an IHCA increased to 23% during the first pandemic wave (April), then abated to 3% in July, and then increased to 19% during the second wave (December). Among COVID+ cases, 43% had respiratory insufficiency or

infection as the underlying cause of the cardiac arrest, compared to 18% among COVID– cases. The most common clinical sign preceding cardiac arrest was hypoxia (57%) among COVID+ cases. Odds ratio for 30-day survival for COVID+ cases was 0.50 (95% CI 0.33-0.76), compared with COVID– cases.

Conclusion: During pandemic peaks, up to one fourth of all IHCAs are complicated by COVID-19, and these patients have halved chance of survival, with women displaying the worst outcomes.

Article Summary

Strengths and limitations of this study

- A major strength of our study is that it includes all IHCAs in Sweden which were reported to the Swedish Registry for Cardiopulmonary Resuscitation.
- The sample recorded in the Swedish Registry for Cardiopulmonary Resuscitation is unbiased since all hospitals participate in the registry and all hospitals report data on COVID-19 status

- A limitation is that we do not know the severity of the COVID-19 infection, and we do not know if COVID-19 was the main reason for admission to hospital.
- Our study only includes IHCAs receiving CPR which leaves out all other patients with IHCA, e.g with a Do Not Attempt Resuscitation order.

Introduction

The COVID-19 pandemic has, as of Nov 6st 2021, infected over 249 million individuals and lead to the death of over 5 million individuals (1). COVID-19 is now the third leading cause of death in Sweden (2, 3). Multiple studies have demonstrated that in-hospital cardiac arrest (IHCA) among patients with COVID-19 is associated with poor survival (4-7). A recent study demonstrated that hypoxia was the main cause of cardiac arrest among 40% of patients with COVID-19 and IHCA (6).

We have previously reported on COVID-19 and IHCA in the Swedish Registry for Cardiopulmonary Resuscitation (SRCR), showing a 2.3-fold increase in 30-day mortality among cases with COVID-19, compared to pre-pandemic cases. This was mainly driven by a 9-fold increase in mortality among women with COVID-19. At the time, no case of IHCA with COVID-19 had been discharged alive (8). The current study expands our previous investigation, including more patients, longer follow-up and emphasizes on the causes of

cardiac arrest, predictors of survival, coexisting conditions, and trends in the prevalence of COVID-19 among IHCA cases.

Methods

Data sources

This study is a registry-based observational study with data obtained from the SRCR during the time period 15/03/2020 to 31/12/2020. The SRCR is a national quality registry and has included IHCA cases since 2005. The data is collected by trained nurses who report patient data using a web-based protocol. The registry has previously been described in detail (9). Vital status was obtained from the Swedish Population Registry and the last day of follow up was 31/12/2020.

Study population

The study population included all patients ≥18 years of age suffering IHCA and receiving CPR throughout Sweden during the period 15/03/2020 to 31/12/2020. We used 15th of March as the start date of the pandemic as the Swedish Public Health Authority declared on March 16th 2020 that community spread had commenced (3). On 1st of April the SRCR started collecting data regarding COVID-19 status, and retrospectively identified 60 patients with COVID-19 who suffered IHCA during March (they were included in the study). Patients were

divided into the following three groups: ongoing infection (COVID+; n=182), no infection (COVID–; n=1062) and unknown/not assessed (UNA; n=369). COVID+ was defined as patients registered with an ongoing COVID-19 infection, suspected ongoing infection or patients with a recent infection (n=29). The UNA group was included in the study in order to provide a complete picture of cases enrolled in the SRCR during the time period, and to evaluate whether missingness in COVID-19 status could entail selection bias.

Variable definitions

In SRCR a patient with cardiac arrest was defined as an unconscious patient with no or abnormal breathing, in whom resuscitation or defibrillation was attempted. IHCA was defined as cardiac arrest in patients admitted to the hospital.

With regards to previous coexisting conditions, heart failure was defined as any heart failure described before cardiac arrest. Kidney failure was defined as estimated glomerular filtration rate (eGFR) below 60 ml/min/1.73 m^2, calculated using the highest creatinine before cardiac arrest with Chronic Kidney Disease Epidemiology Collaboration (CKD-EPI) formula. The SRCR records data on the highest creatinine levels analyzed up to six months prior to CA. Diabetes was defined as any diabetes diagnosis, regardless of type. Cancer was defined as any previously known cancer. Acute myocardial infarction (MI) was defined as an MI within 72

hours of CA. Previous myocardial infarction was defined as MI occurring earlier than 72 hours preceding the CA.

Regarding clinical conditions one hour prior to CA, arrhythmia was defined as any arrhythmia, hypoxia was defined as an oxygen saturation below 90%, hypotension was defined as systolic blood pressure below 90 mmHg, seizure was defined as any seizure with loss of consciousness, and heart failure was defined as any heart failure with pulmonary edema or severe shortness of breath with rales.

Wards with monitoring included the coronary care unit (CCU), intensive care unit (ICU), operating room (OR), emergency room (ER), high dependency unit (HDU) or the catheterization laboratory.

Statistical analyses

Patient characteristics are reported in means and medians, along with standard deviations and interquartile ranges, respectively. The Kaplan-Meier estimator was used for describing survival distributions; the log rank test was used to test for differences in survival. Trends in rates of COVID-19 were assessed on a monthly basis during the entire study basis.

Logistic regression was used to calculate odds ratios for 30-days survival. These models assessed the association between COVID-19 status and 30-days survival, adjusting for age,

sex and initial rhythm (shockable or non-shockable). We performed subgroup analyses in relation to sex, age and coexisting conditions (heart failure, cancer, diabetes, kidney failure and myocardial infarction). These subgroup analyses served to clarify whether the association between COVID status and survival was modified by age, sex or coexisting conditions.

In order to obtain estimates of overall survival, we used Cox proportional hazards model with hours since CA as the time scale. The proportional hazards assumption was fulfilled for all variables.

We used the MICE (Multiple Imputation By Chained Equations) algorithm to impute missing values (10, 11) (Supplementary Figure 1). The imputed data set was used to calculate odds ratios for 30-days survival in the overall group, as well as in COVID+ and COVID– cases. These models included age, sex, initial rhythm, time to start of cardiopulmonary resuscitation (CPR), time of CA, previous MI, type of ward, heart failure, ECG monitoring, diabetes and acute MI.

Analyses were done in R (v. 4.0.3, R Foundation for Statistical Computing) using RStudio.

Patient and Public Involvement statement:

No patients were involved.

Results

A total of 2,227 patients were enrolled in the SRCR between 01/01/2020 and 31/12/2020.

After excluding patients <18 years (n=68) and pre-pandemic cases (n=546), 1,613 cases remained from 15/03/2020 to 31/12/2020 and constituted the final study population (Supplementary Figure 2). There was a high rate of information on COVID-19 status during the study period among patients registered in the registry (Supplementary Figure 3).

Baseline characteristics

The overall mean age was 70.8 years, and the proportion of women was 37.6%. At the end of follow-up, 341 (32.7%) patients were alive. The mean age was similar in the three groups: 70.9 years in COVID+, 71.0 years in COVID– cases, and 70.2 years in cases with UNA (Supplementary Figure 4). The proportion of women was also similar; 37.6% in COVID+ and 36.6% in COVID– and 41.0% in UNA cases.

A regular ward was the most common place for cardiac arrest in all 3 groups; 45.1% of COVID+, 44.1% of COVID– and 31.4% of UNA cases occurred in regular wards (Table 1). The emergency room (ER) was the second most common location for COVID+ cases (15.9%).

Regarding comorbidities, acute myocardial infarction was observed in 12.0% of COVID+ and 23.6% of COVID– cases. Previous myocardial infarction was observed in 11.7% of COVID+, 20.8% of COVID– and 11.7% of UNA cases. The prevalence of heart failure, cancer and diabetes was similar across all groups (Table 1).

Fewer cases among COVID+ individuals had a shockable rhythm (17.3%), compared with COVID– (24.9%) cases. Likewise, fewer cases among COVID+ (22.7%) were defibrillated, compared with COVID– cases (31.5%). COVID+ cases were ventilated in 54.8% of cases before rescue team arrival, as compared with 63.2% in COVID– cases.

Follow-up

Return of spontaneous circulation (ROSC) after initial resuscitation, was less common in COVID+ cases, as compared with COVID– cases. Also, angiography, PCI, pacemaker and ICD implantation post cardiac arrest were less common in COVID+ cases.

Sex specific characteristics

Acute myocardial infarction was observed in 21.2% of COVID+ women and 7.6% of COVID+ men. Previous myocardial infarction was observed in 4.7% of COVID+ women and 16.2% of COVID+ men. The prevalence of previous stroke, renal failure, heart failure, cancer

and diabetes were similar among men and women, as was location at the time of cardiac arrest. COVID+ men were more likely to have a shockable rhythm (20.8%) compared with COVID+ women (11.5%), and to be defibrillated (26.4% in men vs. 16.9% in women) (Supplementary Table 1).

Monthly trends in COVID-19 associated IHCA

In March, April and May 14%, 23% and 20% of patients suffering IHCA were COVID+ (data from 16th March). The proportion of COVID+ cases diminished rapidly during June to July. From September onwards the COVID+ cases increased again to reach 19% in December. In Figure 1A additional details regarding monthly variations are presented.

Etiology of IHCA

The most common cause of IHCA among COVID+ cases was respiratory insufficiency (24%, n=24), and the second most common cause was sepsis or other infection (19%, n=19). Respiratory insufficiency and sepsis/other infection were less common in the other groups (Figure 1B), which instead displayed higher rates of acute myocardial infarction.

Clinical conditions one hour prior to IHCA

As evident in Figure 1C, which describes the clinical conditions preceding (up to 60 minutes) the cardiac arrest, hypoxia was more common among COVID+ cases (57%), as compared with COVID– cases (34%).

Survival analysis

The Kaplan Meier plots (Figure 2) show that COVID+ cases generally had a lower probability of survival compared to both COVID– and UNA cases. The overall 30-day survival (Figure 2A) was 21% among COVID+, compared with 36% in COVID– cases (p=0.00086). The subgroup analysis of women (Figure 2B) showed low survival rates in COVID+ cases (16% 30-day survival). The subgroup analysis of men (Figure 2C) showed low survival rates in COVID+ cases (23% 30-day survival). The 30 days survival among COVID+ aged >70 years was 18% (Figure 2D), as compared with 25% of COVID+ cases aged 70 years or younger (Figure 2E). Survival curves for the subgroups of individuals with

cancer, heart failure and diabetes, did not display any distinct patterns (Figure 2F-2H), with all p values >0.1. Patients with kidney failure had a 30 days survival of 13% among COVID+ cases (Figure 2I). Patients with acute MI had a 30 days survival of 8% among COVID+ cases (Figure 2J).

Cox adjusted survival curves are presented in Supplementary Figure 5; COVID+ cases displayed the lowest probability of survival, whereas there was no material difference between COVID– and UNA cases.

Odds ratios for 30-days survival

When adjusted for age, sex and initial rhythm the odds ratios for 30-day survival, comparing COVID+ vs. COVID–, were 0.50 (0.33-0.76) overall, 0.53 (0.31-0.88) for men, and 0.44 (0.20-0.88) for women. In the subgroup of patients with heart failure, myocardial infarction and cancer, we found no statistically significant associations, whereas in the subgroup of COVID+ patients with kidney failure, odds ratio for 30-days survival was 0.43 (0.16–0.99), when compared with COVID– cases (Figure 3).

Predictors of survival

Regarding predictors of 30-days survival among COVID+ we note that confidence intervals were generally wide. Lack of ECG monitoring and delayed start of CPR showed point

estimates below 1.0, although non-significant. Odds ratio for patients treated in non-monitored wards was 0.26 (95% CI 0.08-0.78) as compared with monitored wards (Figure 4). No coexisting condition was associated with survival among COVID+ cases.

Among COVID– cases, the factors that were significantly associated with 30-days survival were shockable rhythm (OR 4.18 [95% CI 2.69–6.02]), ECG monitoring (2.67 [95% CI 1.82–3.95]), heart failure (OR 0.58 [95% CI 0.40–0.83]) and diabetes (OR 0.64 [95% CI 0.44–0.92]; Figure 4).

Discussion

This study elucidates characteristics and outcomes in patients with COVID-19 who develop IHCA. We show that the prevalence of COVID-19 among patients suffering an IHCA increased to approximately one in four cardiac arrests during the first pandemic wave, and one in five cardiac arrests during the second wave. In IHCA the probability of survival to 30-days is halved by the presence of COVID-19.

Regarding location of CA, we note that the most common location for COVID+ patients was regular wards, which are not monitored. This is unfortunate since our analyses showed that type of ward (monitored vs non-monitored) was significantly associated with survival, such that COVID+ cases in non-monitored wards displayed 74% lower probability of survival as

compared with COVID+ cases in monitored wards. As compared with COVID– cases, cardiac arrest in the ER was more common in COVID+ cases. The often rapid deterioration of cardiopulmonary function in patients with COVID-19 may be one of the explanations for this finding. Fewer COVID+ cases were located in the CCU, which was an expected finding given that cardiac etiology was less common among these patients.

We note that the most common cause of cardiac arrest in COVID+ cases, as well as the most frequent clinical condition directly preceding the arrest, was respiratory. A total of 57% of cases displayed hypoxia before cardiac arrest. This may highlight an opportunity for improving outcomes; measures to prevent hypoxia and to correct it immediately may reduce the risk of cardiac arrest in patients with COVID-19. The high rate of respiratory etiology was driven by men (Supplementary Figure 6-7).

However, the fact that 43% of cases with COVID-19 did not have hypoxia prior to cardiac arrest suggests that other factors are important as well. Thromboembolism, myocardial infarction, arrhythmias, etc. may all contribute to the development of a cardiac arrest (12).

A previous study from Wuhan showed that 87.5% of COVID+ cases with IHCA had a respiratory etiology and a study from Southwest Georgia that 53% of the patients with IHCA and COVID-19 had ARDS (5, 7).

For peer review only - http://bmjopen.bmj.com/site/about/guidelines.xhtml

The survival rates were poor among COVID+ patients with an overall 30-days survival of 21%, compared to 36% among COVID–. The survival rate was, however, not as low as in the study from Wuhan, in which 3% (151 patients studied) survived, or in the study from New York with 31 patients or in the study from Southwest Georgia with 63 patients with none surviving (5, 7, 13). One reason for the poor survival could be the small number of patients found in shockable rhythm (17% vs. 25% for COVID+ and COVID–, respectively) since patients with shockable rhythm have a more favorable outcome. After adjusting for sex, age and shockable rhythm the 30-day survival was still significantly worse among patients with an ongoing infection.

We demonstrate that COVID+ women had halved chance of survival at 30 days, compared with COVID– women. We find it interesting that COVID+ women had acute MI three times as often as men, despite the fact that men exhibited shockable rhythm – and were defibrillated – twice as often as women.

Strengths and limitations. This study includes all IHCAs in Sweden which were reported to SRCR. The sample recorded in the SRCR is unbiased since all hospitals participate in the registry and all hospitals report data on COVID-19 status. However, we do not know the severity of the COVID-19 infection, and we do not know if COVID-19 was the main reason

for admission to hospital. With regards to the classification of COVID-19 status, we have performed a misclassification analysis which demonstrated that odds ratios were not materially affected by misclassification bias. Missingness was prevalent with regards to cause of cardiac arrest, which is due to the difficulties determining this factor. However, we find no reason to believe that missingness differs across COVID status categories, and it should therefore not bias our inferences. Our study only includes IHCAs receiving CPR. This leaves out all other patients with IHCA, e.g with a Do Not Attempt Resuscitation order.

Our regression models that included only COVID-19 cases should be interpreted with caution due to the large number of predictors in the model, with relatively few patients (resulting in wide confidence intervals). Further studies are warranted, using a larger study population, and a longer follow up especially regarding subgroup analyses, neurological outcomes and the quality of life for these patients.

Conclusion

During pandemic peaks, up to one fourth of all IHCAs are complicated by COVID-19, and these patients have halved chance of survival, with women displaying the worst outcomes.

Funding

This work was supported by the Swedish Research Council [2019-02019] and the Swedish Heart and Lung Foundation [20200261].

Conflict of interest: none declared.

Author Statement: Astrid Holm and Araz Rawshani designed the study. Astrid Holm wrote the first draft of the manuscript, analyzed all data and made initial interpretations of data. Araz Rawshani has been supervising. Matilda Jerkeman, Pedram Sultanian, Peter Lundgren, Annica Ravn-Fischer, Johan Israelsson, Jasna Giesecke and Johan Herlitz revised the article critically for important intellectual content and approved the version of the article to be published.

Ethics statement: The study was approved by the Swedish Ethical Review Authority (ID 2020-02017). The data was anonymized before the authors accessed it for the purpose of the study.

Data sharing plan: No additional data available

References

1. Johns Hopkins University and Medicine COVID-19 map [internet] Johns Hopkins Coronavirus Resource Centre 2020. [updated 13/5/2021. Available from: https://coronavirus.jhu.edu/map.html.
2. Dödsorsaker första halvåret 2020. Socialstyrelsen. Hälso- och sjukvård.; 2020 17/11/2020.
3. Andersson J. Samhällsspridning av coronaviruset i Sverige. Läkartidningen. 16/03/2020.
4. Hayek SS, Brenner SK, Azam TU, Shadid HR, Anderson E, Berlin H, et al. In-hospital cardiac arrest in critically ill patients with covid-19: multicenter cohort study. Bmj. 2020;371:m3513.
5. Shah P, Smith H, Olarewaju A, Jani Y, Cobb A, Owens J, et al. Is Cardiopulmonary Resuscitation Futile in Coronavirus Disease 2019 Patients Experiencing In-Hospital Cardiac Arrest? Crit Care Med. 2021;49(2):201-8.
6. Mitchell OJL, Yuriditsky E, Johnson NJ, Doran O, Buckler DG, Neefe S, et al. In-hospital cardiac arrest in patients with coronavirus 2019. Resuscitation. 2021;160:72-8.
7. Shao F, Xu S, Ma X, Xu Z, Lyu J, Ng M, et al. In-hospital cardiac arrest outcomes among patients with COVID-19 pneumonia in Wuhan, China. Resuscitation. 2020;151:18-23.
8. Sultanian P, Lundgren P, Strömsöe A, Aune S, Bergström G, Hagberg E, et al. Cardiac arrest in COVID-19: characteristics and outcomes of in- and out-of-hospital cardiac arrest. A report from the Swedish Registry for Cardiopulmonary Resuscitation. European Heart Journal. 2021.
9. Hessulf F, Herlitz J, Rawshani A, Aune S, Israelsson J, Södersved-Källestedt ML, et al. Adherence to guidelines is associated with improved survival following in-hospital cardiac arrest. Resuscitation. 2020;155:13-21.
10. Stef van Buuren, Groothuis-Oudshoorn K. mice: Multivariate Imputation by Chained Equations in R. Journal of Statistical Software. 2011;Vol 45.
11. 3.5 Classification and regression trees [Available from: https://stefvanbuuren.name/fimd/sec-cart.html.
12. Gupta A, Madhavan MV, Sehgal K, Nair N, Mahajan S, Sehrawat TS, et al. Extrapulmonary manifestations of COVID-19. Nat Med. 2020;26(7):1017-32.
13. Sheth V, Chishti I, Rothman A, Redlener M, Liang J, Pan D, et al. Outcomes of in-hospital cardiac arrest in patients with COVID-19 in New York City. Resuscitation. 2020;155:3-5.

Table 1 Characteristics of 1613 patients with IHCA during the COVID-19 pandemic.

Variables	No infection COVID –	Ongoing infection COVID +	Unknown/NA UNA	SMD
n	1062	182	369	
**Demographics:**				
Age - mean (SD)	71.00 (13.32)	70.93 (12.43)	70.22 (13.60)	0.039
Woman - n (%)	388 (36.6)	68 (37.6)	151 (41.0)	0.061
**Location of cardiac arrest - n (%)**				0.527
Coronary care unit - n (%)	155 (14.6)	14 (7.7)	50 (13.6)	
Intensive care unit - n (%)	77 (7.3)	25 (13.7)	19 (5.1)	
Operational room - n (%)	22 (2.1)	0 (0.0)	12 (3.3)	
Emergency room - n (%)	139 (13.1)	29 (15.9)	65 (17.6)	
Outpatient lab, radiology - n (%)	49 (4.6)	7 (3.8)	28 (7.6)	
Cathlab - n (%)	98 (9.2)	8 (4.4)	60 (16.3)	
Intermediate care unit - n (%)	25 (2.4)	15 (8.2)	10 (2.7)	
Regular ward - n (%)	468 (44.1)	82 (45.1)	116 (31.4)	
Other - n (%)	29 (2.7)	2 (1.1)	9 (2.4)	
**Critical times - median (IQR):**				
Time to alert – median (IQR)	1.00 [1.00, 1.00]	1.00 [1.00, 1.00]	1.00 [1.00, 1.00]	0.078
Time to CPR - median (IQR)	0.00 [0.00, 1.00]	0.00 [0.00, 0.00]	0.00 [0.00, 1.00]	0.109
Time to defibrillation - median (IQR)	2.00 [1.00, 5.00]	2.00 [1.00, 4.75]	1.00 [1.00, 4.00]	0.141
**Comorbidities - n (%):**				
MI, ongoing - n (%)	178 (23.6)	12 (12.0)	37 (29.4)	0.292
MI, previous - n (%)	163 (20.8)	13 (11.7)	26 (18.4)	0.165
Stroke, ongoing - n (%)	30 (3.8)	4 (3.7)	4 (3.0)	0.030
Stroke, previous - n (%)	82 (10.3)	7 (6.1)	15 (10.5)	0.105
Cancer, any - n (%)	165 (20.9)	20 (17.7)	28 (20.6)	0.054
Diabetes - n (%)	224 (27.9)	36 (31.0)	38 (27.0)	0.060
Heart failure - n (%)	229 (29.7)	36 (33.0)	36 (27.9)	0.074
Ejection fraction (%) - mean (SD)	46.14 (13.74)	46.44 (11.86)	44.94 (14.82)	0.073
EF <50% - n (%)	167 (46.0)	26 (48.1)	22 (46.8)	0.029
**Kidney function category - n (%)**				0.121
eGFR <30 - n (%)	165 (21.6)	22 (20.0)	26 (20.0)	
eGFR 30–59- n (%)	216 (28.3)	32 (29.1)	44 (33.8)	
eGFR 60–89 - n (%)	198 (25.9)	25 (22.7)	30 (23.1)	
eGFR ≥90 - n (%)	185 (24.2)	31 (28.2)	30 (23.1)	
No kidney failure (eGFR ≥60) - n (%)	383 (50.1)	56 (50.9)	60 (46.2)	0.063
eGFR (ml/min/m2) - mean (SD)	66.89 (49.43)	71.26 (58.96)	63.78 (40.31)	0.099
**Cause of arrest:** - n (%)				0.629
Hemorrhage - n (%)	34 (4.9)	2 (2.0)	10 (8.1)	
Myocardial infarction/ischemia- n (%)	181 (26.2)	15 (14.9)	41 (33.3)	

infarction/ischemia 181 (26.2) 15 (14.9) 4 (33.				
Other - n (%)	213 (30.8)	30 (29.7)	41 (33.3)	
Primary arrhythmia - n (%)	101 (14.6)	8 (7.9)	12 (9.8)	
Respiratory insufficiency - n (%)	73 (10.5)	24 (23.8)	7 (5.7)	
Sepsis/infection - n (%)	45 (6.5)	19 (18.8)	4 (3.3)	
Stroke/thromboembolism - n (%)	45 (6.5)	3 (3.0)	8 (6.5)	
**Early interventions - n (%):**				
Witnessed arrest - n (%)	857 (80.9)	140 (77.8)	306 (85.0)	0.124
ECG monitoring - n (%)	635 (60.5)	89 (50.0)	221 (62.1)	0.163
CPR before AGA - n (%)	845 (91.0)	146 (93.6)	268 (88.2)	0.127
Defibrillated before AGA – n (%)	159 (17.9)	18 (11.9)	53 (19.0)	0.131
Ventilated before AGA - n (%)	503 (63.2)	74 (54.8)	175 (69.2)	0.199
Shockable rhythm - n (%)	247 (24.9)	29 (17.3)	90 (27.0)	0.158
Defibrillated, any - n (%)	323 (31.5)	40 (22.7)	111 (32.8)	0.151
Intubated - n (%)	473 (47.0)	100 (57.8)	177 (53.8)	0.145
Adrenaline given - n (%)	668 (65.6)	125 (72.7)	223 (66.4)	0.102
Antiarrhythmics - n (%)	139 (14.1)	17 (10.1)	48 (15.4)	0.107
Mechanical compressions – n (%)	109 (10.8)	18 (10.4)	66 (20.0)	0.180
Active temperature control – n (%)	54 (11.3)	5 (10.4)	3 (4.4)	0.173
**Status at rescue team arrival - n (%):**				
Consciousness - n (%)	214 (23.1)	18 (11.7)	57 (19.3)	0.204
Breathing - n (%)	288 (31.2)	30 (19.5)	84 (28.7)	0.181
Pulse - n (%)	309 (33.8)	36 (23.4)	89 (30.4)	0.154
**Follow-Up data - n (%):**				
Angiography - n (%)	115 (24.2)	8 (16.7)	15 (20.8)	0.124
PCI - n (%)	87 (18.2)	4 (8.3)	16 (21.9)	0.258
Pacemaker implanted - n (%)	80 (16.7)	2 (4.2)	4 (5.6)	0.281
ICD implanted - n (%)	36 (7.5)	1 (2.1)	2 (2.8)	0.172
ROSC - n (%)	520 (49.0)	64 (35.2)	142 (38.5)	0.188
Death at 30 days - n (%)	666 (62.7)	141 (77.5)	237 (64.2)	0.218
Death overall - n (%)	703 (66.2)	141 (77.5)	241 (65.3)	0.181

SD = standard deviation; IQR = interquartile range; SMD = standardized mean difference (difference between the means for the two groups divided by their mutual standard deviation. Values below 0.1 (10%) are considered inconsequential (i.e., no significant difference between the groups)). CPR = Cardiopulmonary resuscitation, PCI = Percutaneous Coronary Intervention, ICD = implantable cardioverter-defibrillator. ROSC = return of spontaneous circulation. AGA= alarm group arrival

Figure Titles and Legends

Figure 1: Characteristics of IHCA according to COVID-19 status

Figure 1A: Monthly proportion of COVID-19 status among patients with IHCA, stratified on COVID-19 status. In March only cases after 15/03/2020 were included.

Figure 1B: Etiology of IHCA, stratified on COVID-19 status. The y-axis shows percentages for each etiology in each group.

Figure 1C: Clinical conditions 1 hour prior to IHCA, stratified on COVID-19 status. Only patients with data regarding the specific condition was included.

Figure 2: Kaplan Meier survival curves

Figure 2: Kaplan Meier survival curves, separately for (A) Overall, (B) Women, (C) Men, (D) Age ≥70 year, (E) Age <70 year, (F) Cancer, (G) Heart failure, (H) Diabetes, (I) Kidney failure and (J) Myocardial infarction. p= log-rank p-value. The numbers under the graphs are showing the survival in percentages. Regarding myocardial infarction acute MI is presented.

Figure 3: Odds Ratio for 30-day survival

Figure 3: Forest plot with the adjusted odds ratio for 30-day survival among patients with ongoing infection vs. no infection and unknown/NA vs. no infection. Stratified on overall, men, women, age < 70 years, age ≥ 70 years, heart failure, kidney failure, diabetes, myocardial infarction and cancer. Myocardial infarction was defined as acute or previous MI.

Figure 4: Odds Ratio for 30-day survival

Figure 4: Forest plot with odds ratio for 30-day survival, stratified on the groups, no infection, ongoing infection and overall, all in different colors. The 95% Confidence interval is shown between the bars. X-axis has a logarithmic scale. ECG= electrocardiogram, CA= cardiac arrest, MI= myocardial infarction. CI= confidence interval.

For peer review only - http://bmjopen.bmj.com/site/about/guidelines.xhtml

Figure 1: Characteristics of IHCA according to COVID-19 status

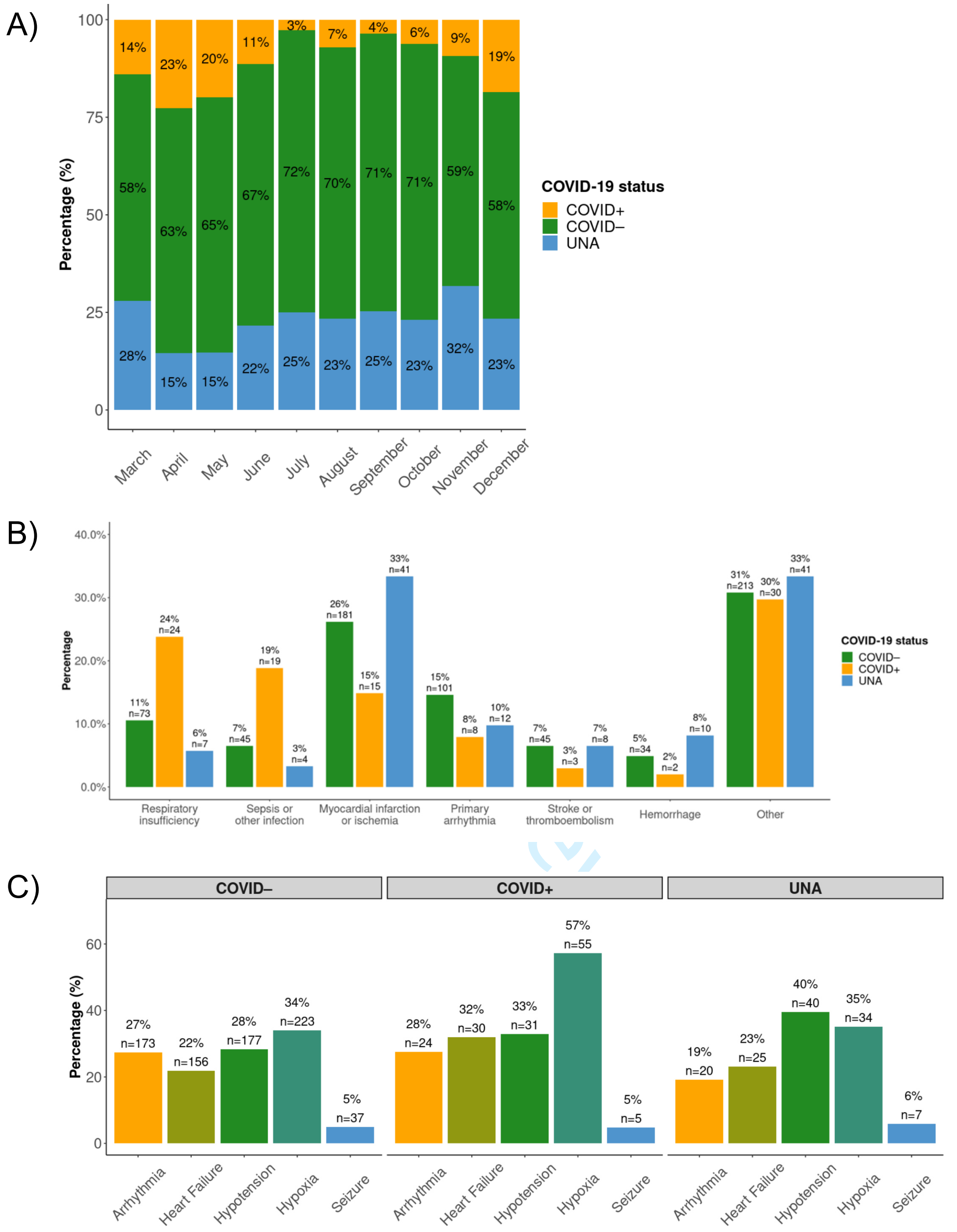

Figure 1A: Monthly proportion of COVID-19 status among patients with IHCA, stratified on COVID-19 status. In March only cases after 15/03/2020 were included.

Figure 1B: Etiology of IHCA, stratified on COVID-19 status. The y-axis shows percentages for each etiology in each group.

Figure 1C: Clinical conditions 1 hour prior to IHCA, stratified on COVID-19 status. Only patients with data regarding the specific condition was included.

For peer review only - http://bmjopen.bmj.com/site/about/guidelines.xhtml

Figure 2: Kaplan Meier survival curves

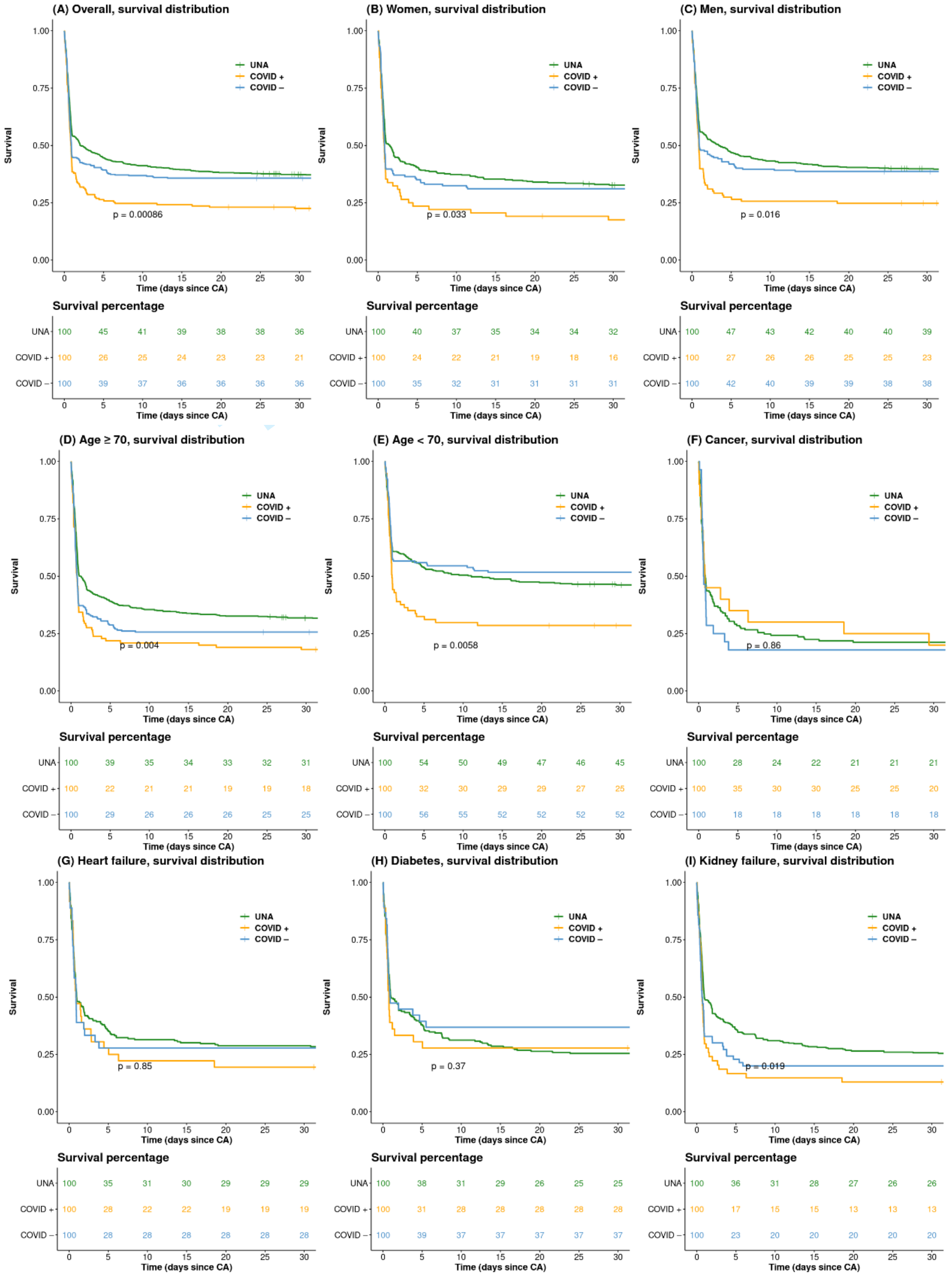

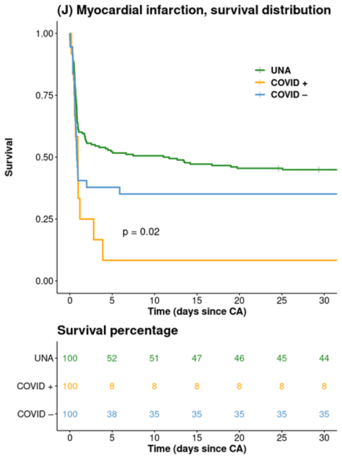

Figure 2: Kaplan Meier survival curves, separately for (A) Overall, (B) Women, (C) Men, (D) Age ≥70 year, (E) Age <70 year, (F) Cancer, (G) Heart failure, (H) Diabetes, (I) Kidney failure and (J) Myocardial infarction. p= log-rank p-value. The numbers under the graphs are showing the survival in percentages. Regarding myocardial infarction acute MI is presented.

Figure 3: Odds Ratio for 30-day survival

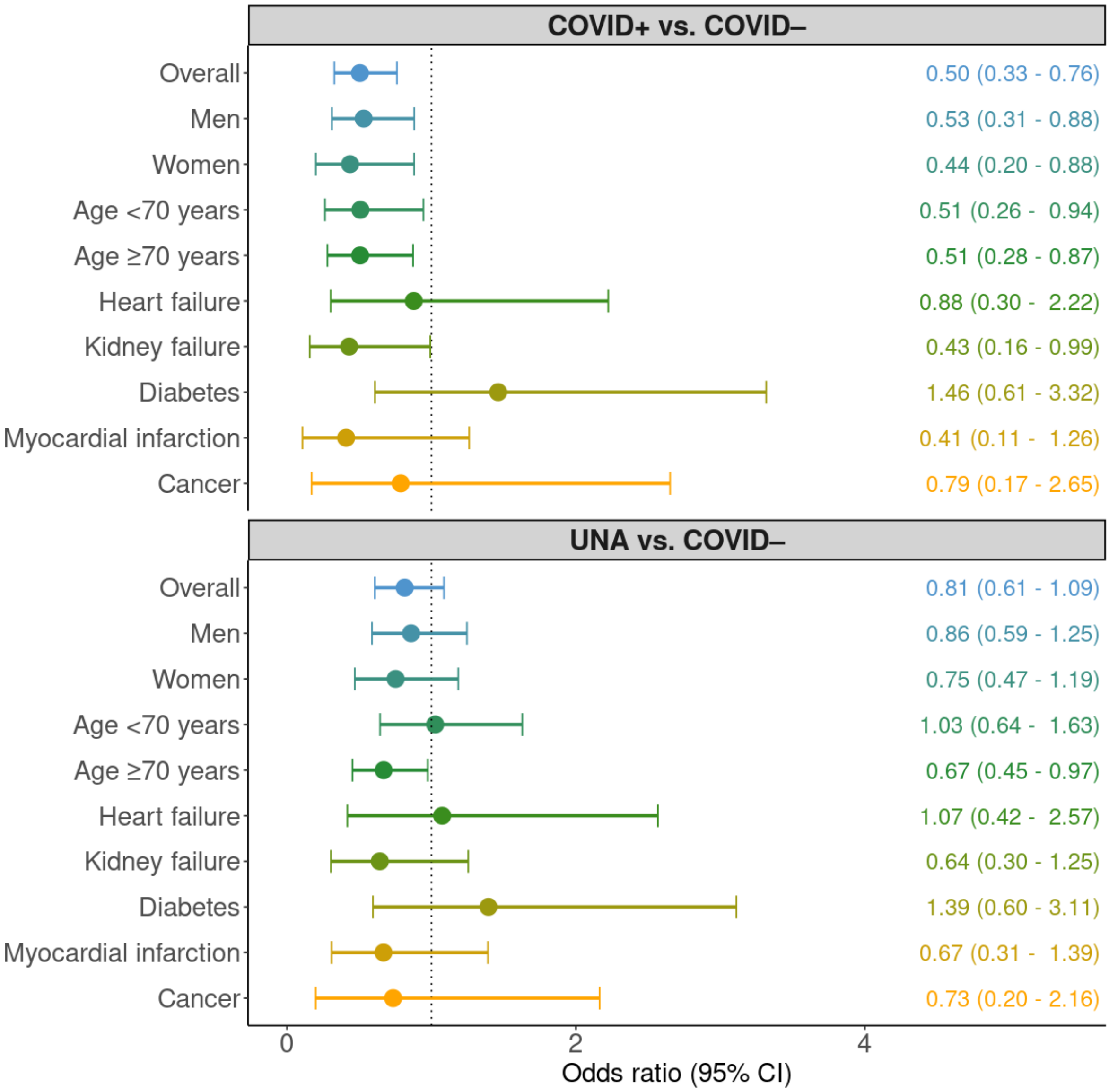

Figure 3: Forest plot with the adjusted odds ratio for 30-day survival among patients with ongoing infection vs. no infection and unknown/NA vs. no infection. Stratified on overall, men, women, age < 70 years, age ≥ 70 years, heart failure, kidney failure, diabetes, myocardial infarction and cancer. Myocardial infarction was defined as acute or previous MI.

Figure 4: Odds Ratio for 30-day survival

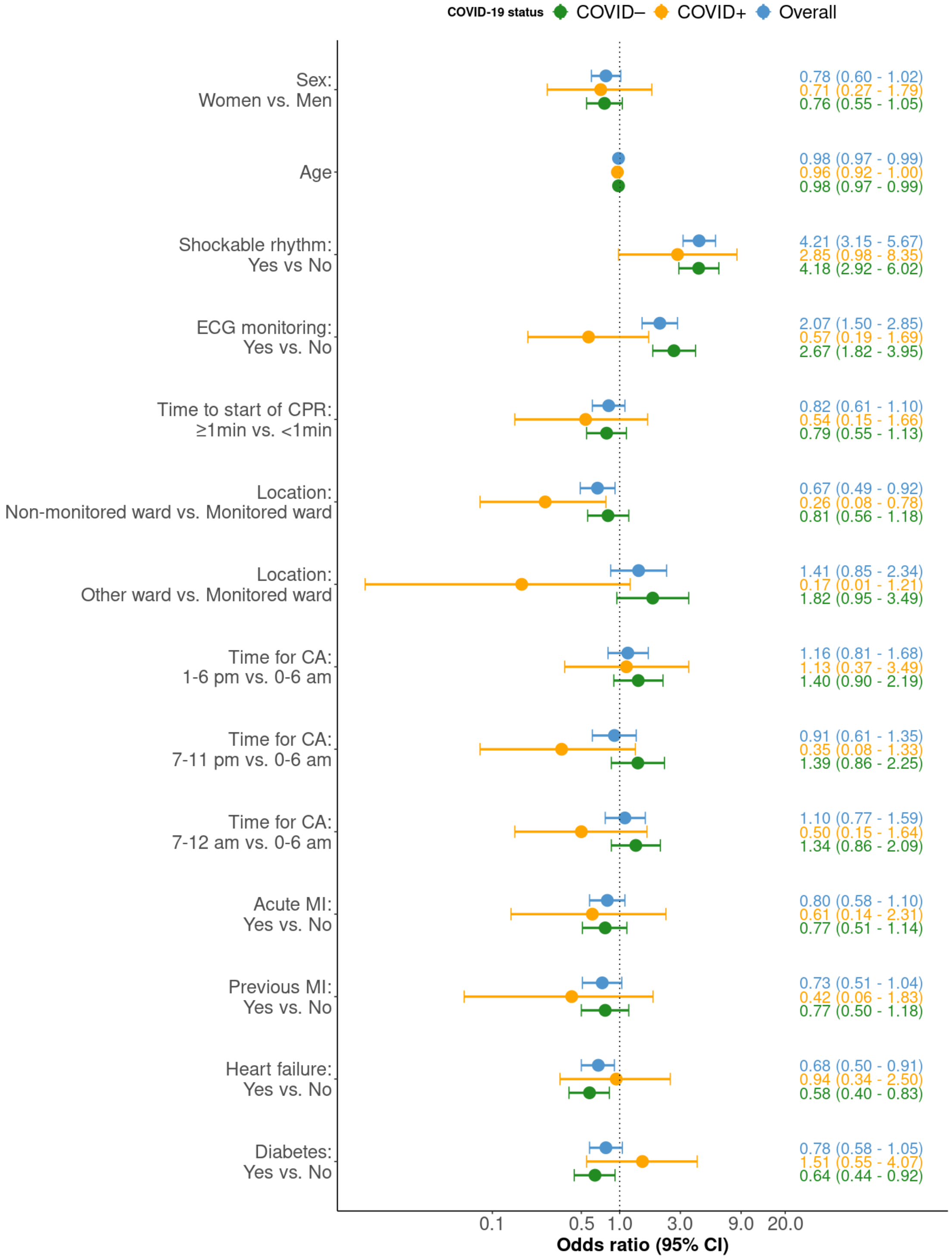

Figure 4: Forest plot with odds ratio for 30-day survival, stratified on the groups, no infection, ongoing infection and overall, all in different colors. The 95% Confidence interval is shown between the bars. X-axis has a logarithmic scale. ECG= electrocardiogram, CA= cardiac arrest, MI= myocardial infarction. CI= confidence interval.

Supplementary figures and tables

Characteristics and Outcomes in Patients with COVID-19 and In-Hospital Cardiac Arrest

For peer review only

Supplementary Table 1: Characteristics of COVID+ patients with IHCA in relation to sex.

Supplementary Table 1: Characteristics of 181 COVID+ patients with IHCA during the COVID-19 pandemic in relation to sex. One COVID+ patient had missing data on sex.

variables	Men	Women	SMD
**n**	113	68	
**Demographics:**			
Age - mean (SD)	71.39 (10.75)	70.35 (14.87)	0.080
**Location of cardiac arrest - n (%):**			0.249
Coronary care unit	7 (6.2)	7 (10.3)	
Intensive care unit	15 (13.3)	10 (14.7)	
Operational room	0 (0.0)	0 (0.0)	
Emergency room	17 (15.0)	11 (16.2)	
Outpatient lab, radiology	4 (3.5)	3 (4.4)	
Cathlab	6 (5.3)	2 (2.9)	
Intermediate care unit	11 (9.7)	4 (5.9)	
Regular ward	52 (46.0)	30 (44.1)	
Other	1 (0.9)	1 (1.5)	
**Critical times - median (IQR):**			
Time to alert – median (IQR)	1.00 [1.00, 1.00]	1.00 [1.00, 1.00]	0.256
Time to CPR - median (IQR)	0.00 [0.00, 0.00]	0.00 [0.00, 1.00]	0.031
Time to defibrillation - median	2.00 [1.00, 5.00]	2.00 [1.00, 2.00]	0.009
**Comorbidities - n (%):**			
MI, ongoing - n (%)	5 (7.6)	7 (21.2)	0.396
MI, previous - n (%)	11 (16.4)	2 (4.7)	0.391
Stroke, ongoing - n (%)	4 (5.8)	0 (0.0)	0.351
Stroke, previous - n (%)	5 (7.0)	2 (4.7)	0.102
Cancer, any - n (%)	13 (18.8)	6 (14.0)	0.132
Diabetes - n (%)	24 (33.3)	12 (27.9)	0.118
Heart failure - n (%)	23 (33.8)	13 (32.5)	0.028
Ejection fraction (%) - mean (SD)	44.84 (12.22)	49.31 (10.56)	0.392
EF <50% - n (%)	19 (51.4)	7 (43.8)	0.153
**Kidney function category - n (%):**			0.357
eGFR <30	16 (22.9)	6 (15.0)	
eGFR 30–59	17 (24.3)	15 (37.5)	
eGFR 60–89	18 (25.7)	7 (17.5)	
eGFR ≥90	19 (27.1)	12 (30.0)	
No kidney failure (eGFR ≥60)	37 (52.9)	19 (47.5)	0.107
eGFR (ml/min/m2) - mean (SD)	72.72 (65.75)	68.70 (45.34)	0.071
**Cause of arrest - n (%):**			0.920
Hemorrhage	1 (1.5)	1 (2.9)	
Myocardial infarction/ischemia	7 (10.6)	8 (23.5)	
Other	18 (27.3)	12 (35.3)	
Primary arrhythmia	3 (4.5)	5 (14.7)	
Respiratory insufficiency	17 (25.8)	7 (20.6)	

For peer review only - http://bmjopen.bmj.com/site/about/guidelines.xhtml

Sepsis / infection	18 (27.3)	1 (2.9)	
Stroke / thromboembolism	2 (3.0)	0 (0.0)	
**Early interventions - n (%):**			
Witnessed arrest - n (%)	86 (76.8)	53 (79.1)	0.056
ECG monitoring - n (%)	56 (50.5)	33 (50.0)	0.009
CPR before AGA - n (%)	90 (92.8)	55 (94.8)	0.085
Defibrillated before AGA - n (%)	13 (13.8)	5 (8.9)	0.155
Ventilated before AGA- n (%)	49 (56.3)	25 (53.2)	0.063
Shockable rhythm - n (%)	22 (20.8)	7 (11.5)	0.254
Defibrillated, any - n (%)	29 (26.4)	11 (16.9)	0.231
Intubated - n (%)	61 (57.0)	38 (58.5)	0.029
Adrenaline given - n (%)	76 (70.4)	48 (76.2)	0.132
Antiarrhythmics - n (%)	11 (10.4)	6 (9.7)	0.023
Mechanical compressions - n (%)	12 (10.9)	5 (8.1)	0.097
Active temperature control - n (%)	2 (6.1)	3 (20.0)	0.423
**Status at rescue team arrival - n**			
Consciousness - n (%)	11 (11.3)	6 (10.7)	0.020
Breathing - n (%)	18 (18.6)	11 (19.6)	0.028
Pulse - n (%)	22 (22.7)	13 (23.2)	0.013
**Follow-Up data - n (%):**			
Angiography - n (%)	4 (12.1)	4 (26.7)	0.374
PCI - n (%)	2 (6.1)	2 (13.3)	0.248
Pacemaker implanted - n (%)	0 (0.0)	2 (13.3)	0.555
ICD implanted - n (%)	0 (0.0)	1 (6.7)	0.378
ROSC - n (%)	40 (35.4)	24 (35.3)	0.002
Death at 30 days - n (%)	85 (75.2)	56 (82.4)	0.175
Death overall - n (%)	85 (75.2)	56 (82.4)	0.175
Discharged alive - n (%)	16 (22.2)	6 (14.0)	0.216

SD = standard deviation; IQR = interquartile range; SMD = standardized mean difference (difference between the means for the two groups divided by their mutual standard deviation. Values below 0.1 (10%) are considered inconsequential (i.e., no significant difference between the groups)). CPR = cardiopulmonary resuscitatio*n*, PCI = percutaneous coronary intervention, ICD = implantable cardioverter-defibrillator. ROSC = return of spontaneous circulation. AGA= alarm group arrival.

Supplementary Figure 1: Missing data before and after imputation with MICE

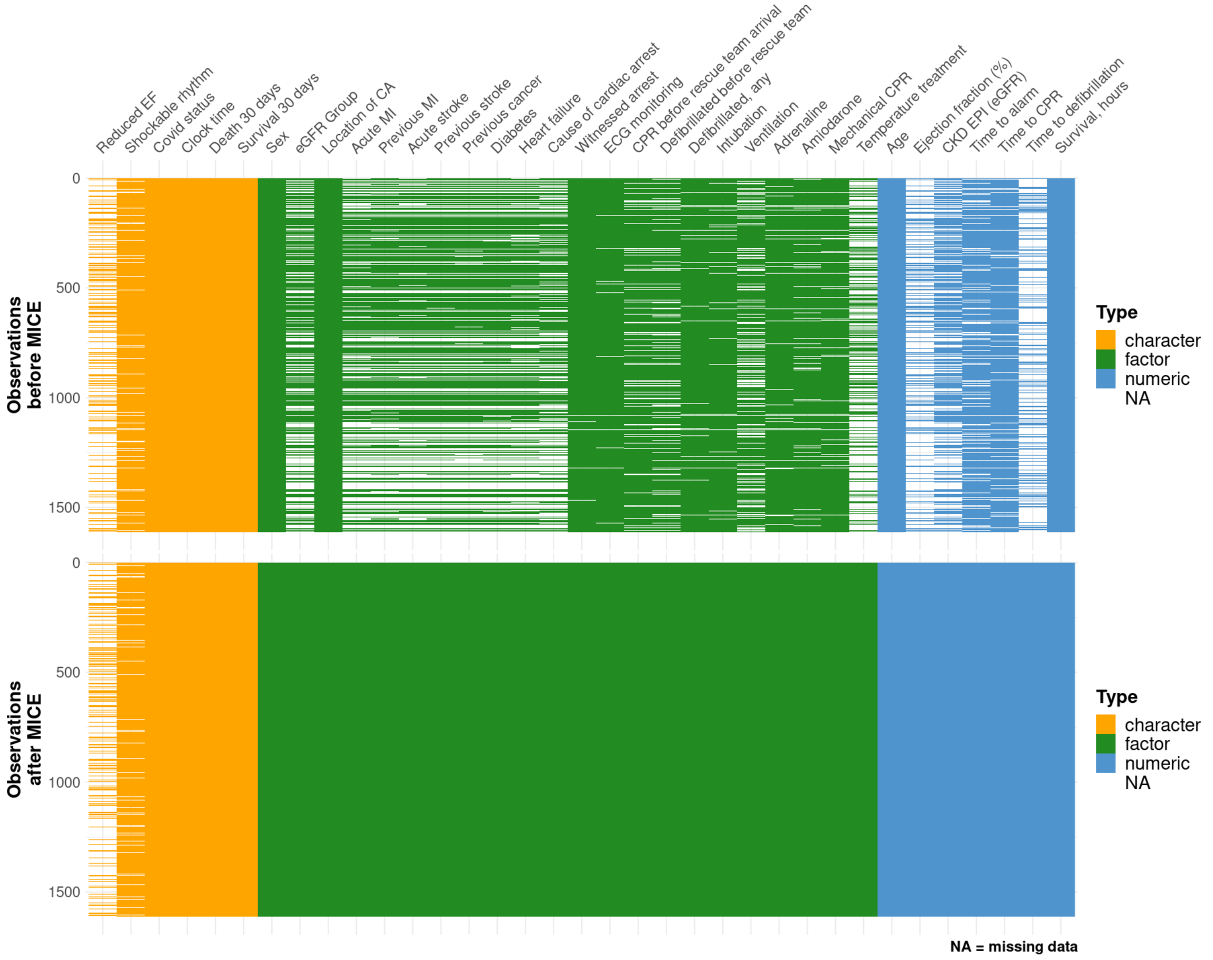

Supplementary Figure 1: Missing data before and after imputation with MICE. A graphical view of the entire dataset is printed. Each column (variable) is depicted at the top and column color depicts type of variable. Each patient represents a row and white gaps indicate a missing data entry.

Supplementary Figure 2: Flow chart

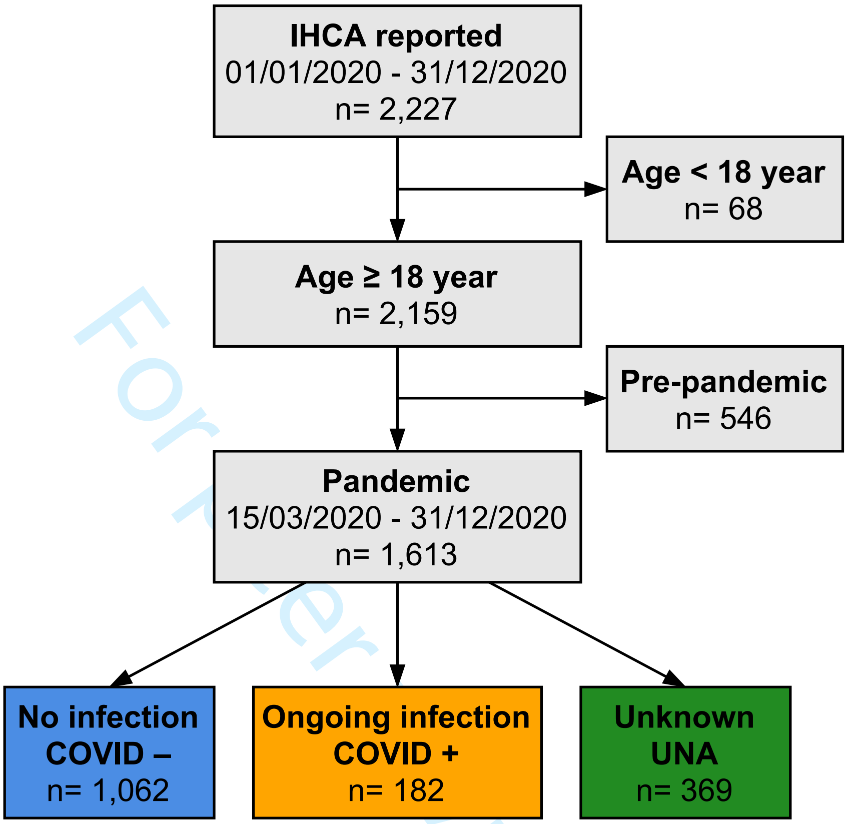

Supplementary Figure 2: Flow chart of the study population. Patients who were less than 18 year of age, and cases occurring in the pre-pandemic period were excluded.

For peer review only

Supplementary Figure 3: Information on COVID-19 status during the study period.

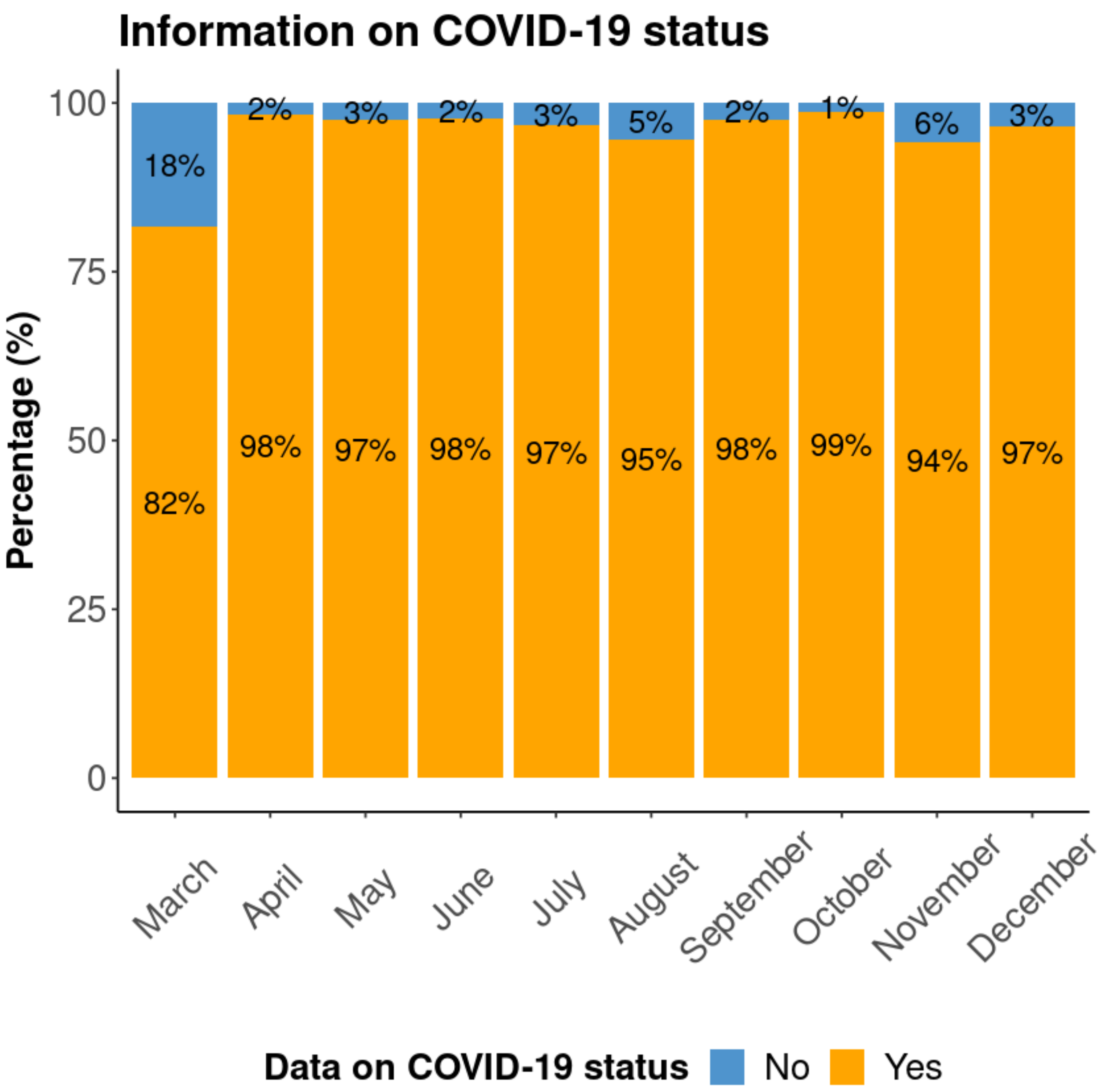

Supplementary Figure 3: Information on COVID-19 status during the study period. No equals missing data, i.e. no information on COVID-19 status available. Yes equals, COVID +, COVID – or Unknown. In March only cases after 15/03/2020 were included.

Supplementary Figure 4: Distribution of age

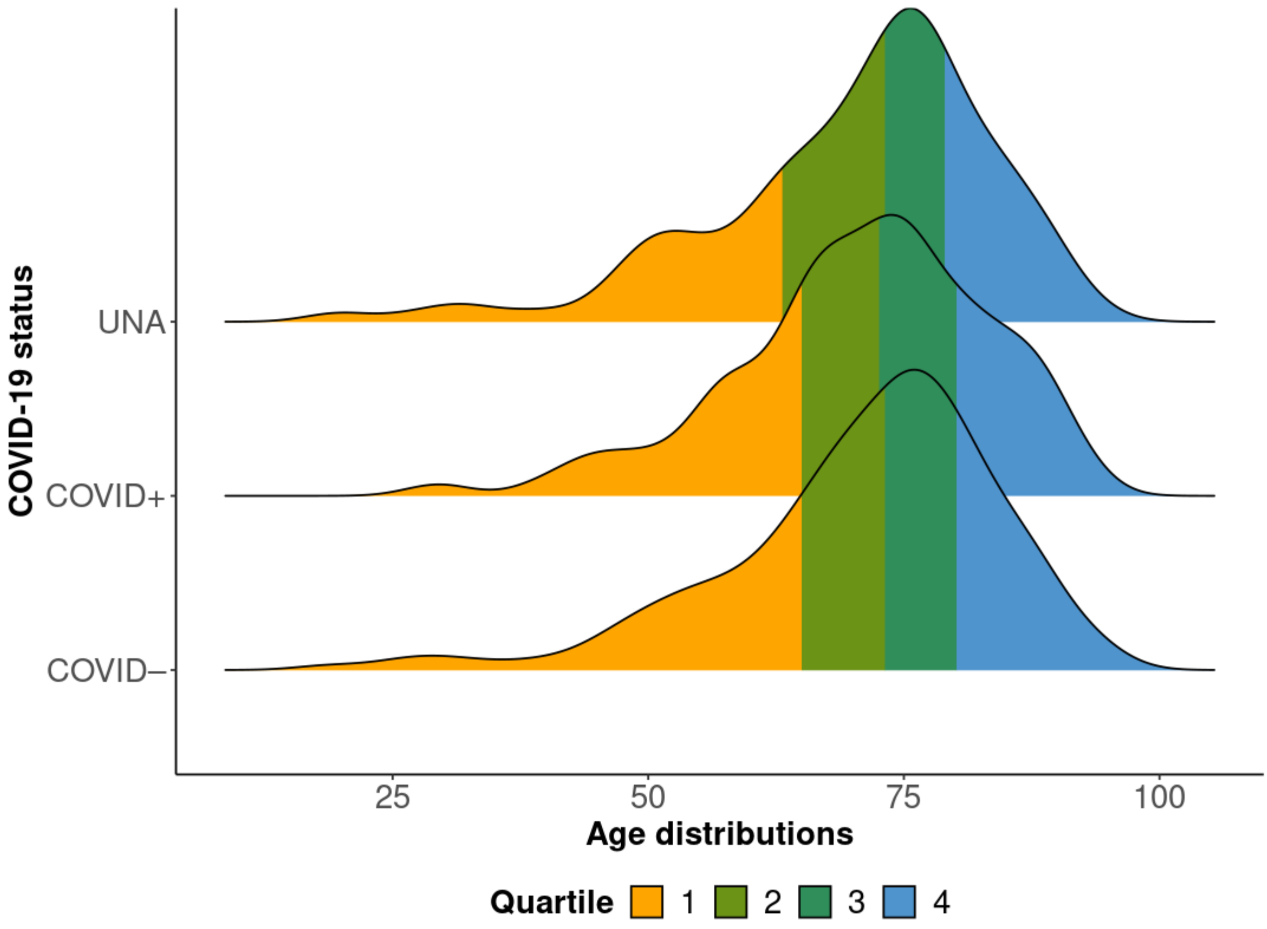

Supplementary Figure 4: Distribution of age in relation to COVID-19 status.

Supplementary Figure 5: Etiology of IHCA, according to sex

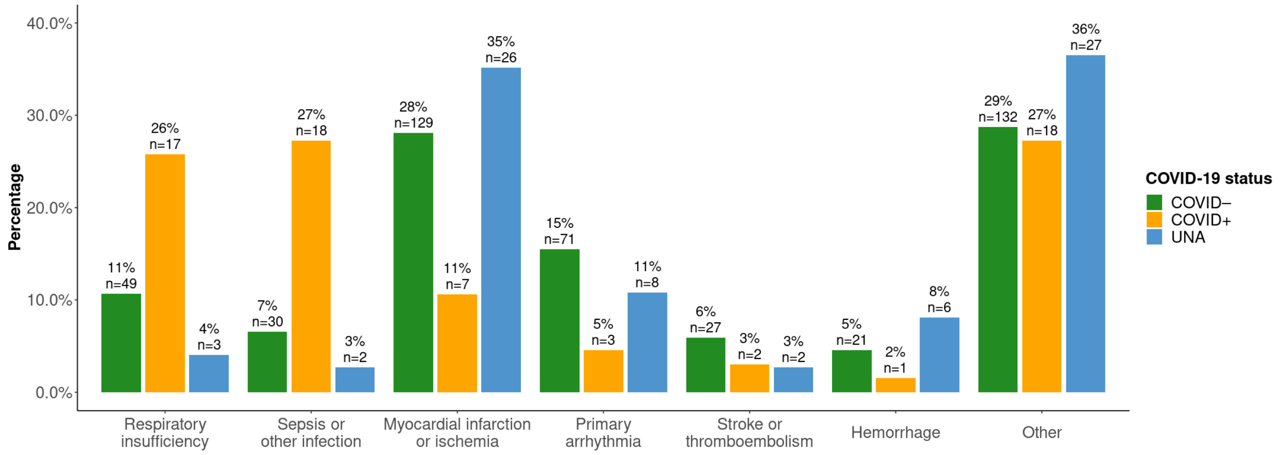

Supplementary Figure 5A: Etiology of IHCA, men only.

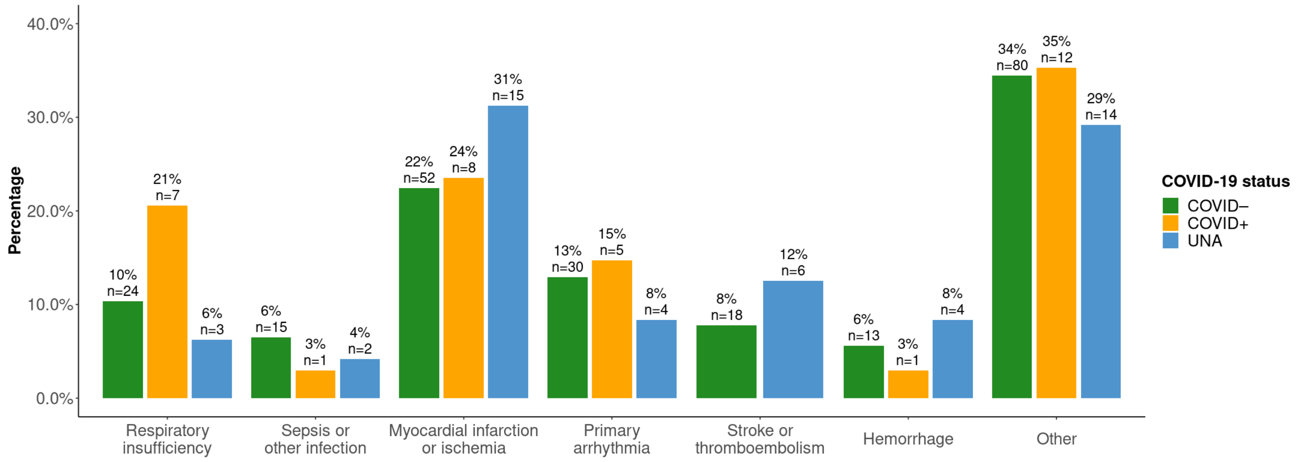

Supplementary Figure 5B: Etiology of IHCA, women only.

Supplementary Figure 6: Conditions preceding IHCA, according to sex

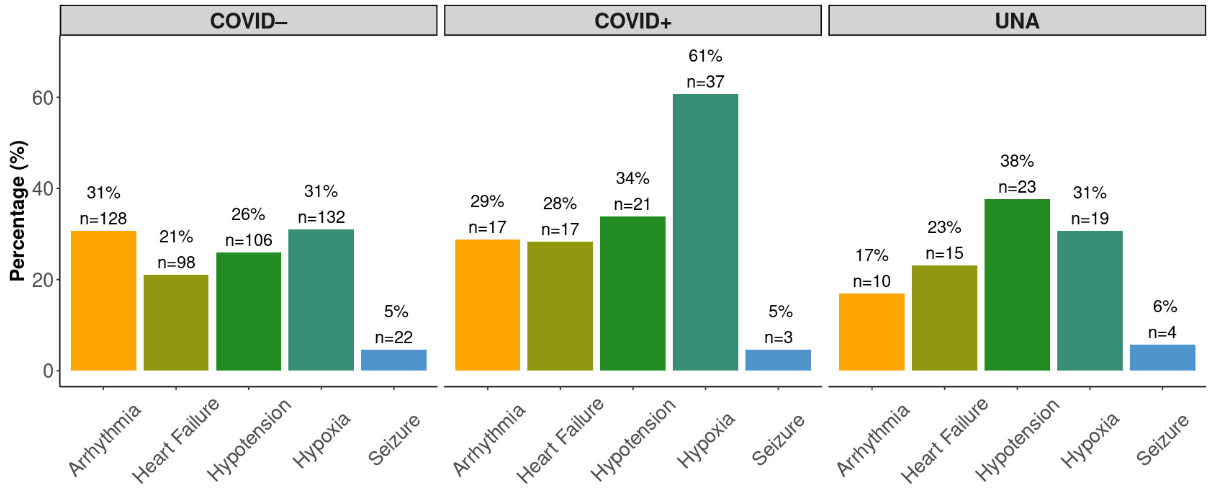

Supplementary Figure 6A: Conditions preceding IHCA, men only.

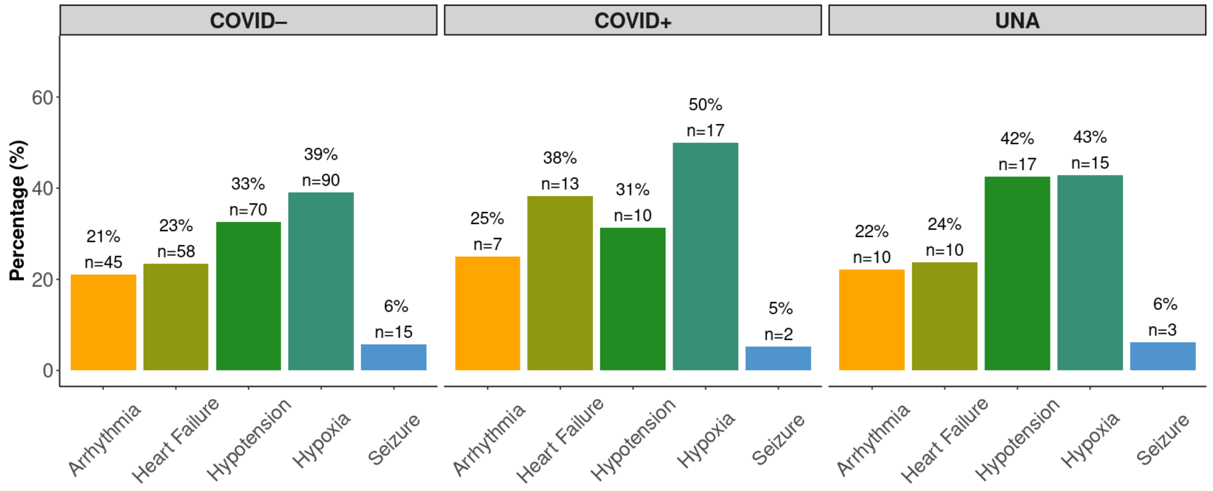

Supplementary Figure 6B: Conditions preceding IHCA, women only.

Supplementary Figure 7: Cox adjusted survival curve for the overall population

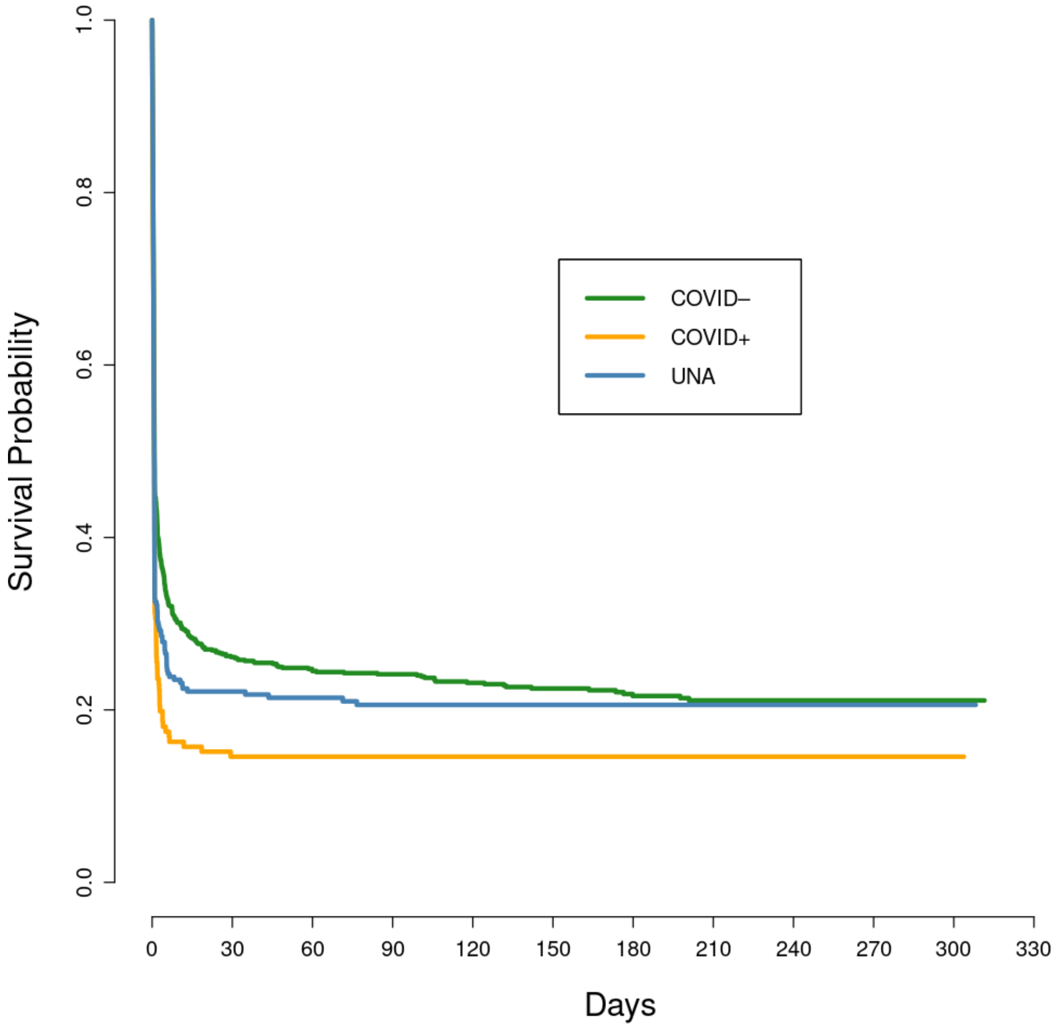

Supplementary Figure 7: Cox adjusted survival curve for the overall population, stratified on COVID-19 status.

STROBE Statement—checklist of items that should be included in reports of observational studies

	Item No	Recommendation	Page No
**Title and abstract**	1	(*a*) Indicate the study's design with a commonly used term in the title or the abstract	1
		(*b*) Provide in the abstract an informative and balanced summary of what was done and what was found	2-3
**Introduction**			
Background/rationale	2	Explain the scientific background and rationale for the investigation being reported	2
Objectives	3	State specific objectives, including any prespecified hypotheses	2
**Methods**			
Study design	4	Present key elements of study design early in the paper	2
Setting	5	Describe the setting, locations, and relevant dates, including periods of recruitment, exposure, follow-up, and data collection	2
Participants	6	(*a*) *Cohort study*—Give the eligibility criteria, and the sources and methods of selection of participants. Describe methods of follow-up<br>*Case-control study*—Give the eligibility criteria, and the sources and methods of case ascertainment and control selection. Give the rationale for the choice of cases and controls<br>*Cross-sectional study*—Give the eligibility criteria, and the sources and methods of selection of participants	2
		(*b*) *Cohort study*—For matched studies, give matching criteria and number of exposed and unexposed<br>*Case-control study*—For matched studies, give matching criteria and the number of controls per case	2
Variables	7	Clearly define all outcomes, exposures, predictors, potential confounders, and effect modifiers. Give diagnostic criteria, if applicable	5-6
Data sources/ measurement	8*	For each variable of interest, give sources of data and details of methods of assessment (measurement). Describe comparability of assessment methods if there is more than one group	5
Bias	9	Describe any efforts to address potential sources of bias	3-4
Study size	10	Explain how the study size was arrived at	5
Quantitative variables	11	Explain how quantitative variables were handled in the analyses. If applicable, describe which groupings were chosen and why	5-6
Statistical methods	12	(*a*) Describe all statistical methods, including those used to control for confounding	6-7
		(*b*) Describe any methods used to examine subgroups and interactions	6-7
		(*c*) Explain how missing data were addressed	6-7
		(*d*) *Cohort study*—If applicable, explain how loss to follow-up was addressed<br>*Case-control study*—If applicable, explain how matching of cases and controls was addressed<br>*Cross-sectional study*—If applicable, describe analytical methods taking account of sampling strategy	6-7
		(*e*) Describe any sensitivity analyses	6-7

Continued on next page

For peer review only - http://bmjopen.bmj.com/site/about/guidelines.xhtml

**Results**			
Participants	13*	(a) Report numbers of individuals at each stage of study—eg numbers potentially eligible, examined for eligibility, confirmed eligible, included in the study, completing follow-up, and analysed	7
		(b) Give reasons for non-participation at each stage	7
		(c) Consider use of a flow diagram	Supplementary material
Descriptive data	14*	(a) Give characteristics of study participants (eg demographic, clinical, social) and information on exposures and potential confounders	7-8
		(b) Indicate number of participants with missing data for each variable of interest	7-8
		(c) *Cohort study*—Summarise follow-up time (eg, average and total amount)	7
Outcome data	15*	*Cohort study*—Report numbers of outcome events or summary measures over time	*8-9*
		Case-control study—Report numbers in each exposure category, or summary measures of exposure	
		Cross-sectional study—Report numbers of outcome events or summary measures	
Main results	16	(*a*) Give unadjusted estimates and, if applicable, confounder-adjusted estimates and their precision (eg, 95% confidence interval). Make clear which confounders were adjusted for and why they were included	7-10
		(*b*) Report category boundaries when continuous variables were categorized	
		(*c*) If relevant, consider translating estimates of relative risk into absolute risk for a meaningful time period	
Other analyses	17	Report other analyses done—eg analyses of subgroups and interactions, and sensitivity analyses	Supplementary material
**Discussion**			
Key results	18	Summarise key results with reference to study objectives	11
Limitations	19	Discuss limitations of the study, taking into account sources of potential bias or imprecision. Discuss both direction and magnitude of any potential bias	13-14
Interpretation	20	Give a cautious overall interpretation of results considering objectives, limitations, multiplicity of analyses, results from similar studies, and other relevant evidence	11-14
Generalisability	21	Discuss the generalisability (external validity) of the study results	14
**Other information**			
Funding	22	Give the source of funding and the role of the funders for the present study and, if applicable, for the original study on which the present article is based	14

*Give information separately for cases and controls in case-control studies and, if applicable, for exposed and unexposed groups in cohort and cross-sectional studies.

Note: An Explanation and Elaboration article discusses each checklist item and gives methodological background and published examples of transparent reporting. The STROBE checklist is best used in conjunction with this article (freely

For peer review only - http://bmjopen.bmj.com/site/about/guidelines.xhtml

available on the Web sites of PLoS Medicine at http://www.plosmedicine.org/, Annals of Internal Medicine at http://www.annals.org/, and Epidemiology at http://www.epidem.com/). Information on the STROBE Initiative is available at www.strobe-statement.org.

For peer review only
